# The spatiotemporal pattern of surface ozone and its impact on agricultural productivity in China

**DOI:** 10.1093/pnasnexus/pgad435

**Published:** 2023-12-14

**Authors:** Xiaoguang Chen, Jing Gao, Luoye Chen, Madhu Khanna, Binlei Gong, Maximilian Auffhammer

**Affiliations:** Research Institute of Economics and Management, Southwestern University of Finance and Economics, 610074 Chengdu, China; Research Institute of Economics and Management, Southwestern University of Finance and Economics, 610074 Chengdu, China; Carbon Neutrality and Climate Change Thrust, Society Hub, Hong Kong University of Science and Technology (Guangzhou), 511453 Guangzhou, China; Department of Agricultural and Consumer Economics, University of Illinois at Urbana-Champaign, Urbana, IL 61801, USA; China Academy for Rural Development (CARD) and School of Public Affairs, Zhejiang University, 310025 Hangzhou, China; Department of Agricultural and Resource Economics, University of California, Berkeley, CA 94720, USA; National Bureau of Economic Research, Cambridge, MA 02138, USA

**Keywords:** air pollution, satellite-based O_3_ estimation, agricultural productivity, China

## Abstract

The slowing of agricultural productivity growth globally over the past two decades has brought a new urgency to detect its drivers and potential solutions. We show that air pollution, particularly surface ozone (O_3_), is strongly associated with declining agricultural total factor productivity (TFP) in China. We employ machine learning algorithms to generate estimates of high-resolution surface O_3_ concentrations from 2002 to 2019. Results indicate that China's O_3_ pollution has intensified over this 18-year period. We coupled these O_3_ estimates with a statistical model to show that rising O_3_ pollution during nonwinter seasons has reduced agricultural TFP by 18% over the 2002–2015 period. Agricultural TFP is projected to increase by 60% if surface O_3_ concentrations were reduced to meet the WHO air quality standards. This productivity gain has the potential to counter expected productivity losses from 2°C warming.

**Significance Statement**

Understanding the drivers of the slowdown in global agricultural productivity in recent years is critical for effective agricultural policy design. We develop high-performance machine learning models to estimate surface ozone (O_3_) concentrations and find a strong, robust negative association between O_3_ and agricultural productivity in China. In particular, we estimate that O_3_ has reduced China's agricultural total factor productivity (TFP) by 18% over the 2002–2015 period, greatly exceeding the combined productivity losses from PM_2.5_ and temperature extremes. If China's surface O_3_ concentrations can meet the WHO air quality standards, the country's agricultural TFP is projected to increase by 60%. Our results suggest that reducing air pollution, especially O_3_, can significantly enhance agricultural productivity in China.

## Introduction

Sustaining productivity growth in agriculture is vital to meeting the world's growing demand for food, feed, fiber, timber, and fuel (1–6). Continuous investments in agricultural research, coupled with improved policies, have greatly boosted agricultural productivity growth in many countries around the world (7). However, this growth in productivity has begun to level off in recent years (3) and shown great sensitivity to air pollution and temperature extremes (8–12), even in the United States (13–15). This is of particular concern as global demand for agricultural products is projected to increase with growing population, rising incomes, and rapid urbanization (16).

Current understanding of the impacts of air pollution and temperature extremes on agricultural productivity is lacking in two major aspects. First, to date, these efforts have overwhelmingly focused on partial productivity measures such as yields of a few staple crops, or profitability in the cropping sector (12, 15, 17, 18). Other sectors largely ignored by this literature, including livestock, forestry, and fisheries, jointly account for nearly 40% of global agricultural output by value (19). Thus, recent studies in this area are inadequate to assess how pollution and temperature extremes affect the overall productivity in the agricultural sector. Second, total factor productivity (TFP) that measures aggregate output per unit of aggregate input has been proven to better reflect production efficiency and technological progress than partial productivity measures (1, 20). Yet, prior studies assessing the sensitivity of agricultural TFP to environmental factors have exclusively focused on climate factors, neglecting the influence of air pollution on TFP (11, 13, 14, 20).

This study examines the impacts of surface ozone (O_3_), fine particulate matter (PM_2.5_), and temperature on China's agricultural productivity. China provides an ideal setting for evaluating the impacts of pollution and temperature extremes on agricultural productivity. As the world's largest agricultural economy, China is a dominant producer of rice, wheat, and vegetables globally, and has been the world's largest livestock producer since overtaking the United States and Europe in the early 1990s (21). China's agricultural productivity has experienced remarkable growth since the introduction of the Household Responsibility System in 1978 that reallocated collectively owned land to individual households, endowing them with autonomy in production and management decisions (22, 23). However, there are signs that this growth has plateaued since the early 2000s (24).

In this article, we focus on O_3_ and PM_2.5_, as they are the two primary air pollutants in China and have been shown to adversely impact crop yields (12, 15, 17, 18) (although it is worth noting that PM_2.5_ may indirectly enhance crop productivity by increasing diffuse radiation). China's national air quality action plan implemented in 2013, which set targets for particulate pollution reductions, has lowered the nation's annual population-weighted average PM_2.5_ concentrations by 32% between 2013 and 2017 (25). However, during this same period, warmer-season surface O_3_ pollution has grown significantly. Ground-level pollution data show that the mean maximum daily average 8-h (MDA8) O_3_ concentrations during nonwinter seasons, especially in summer, have frequently and significantly exceeded the WHO global air quality guidelines in the North China Plain (26), a major agricultural production region. These guidelines set a threshold of 60 μg/m^3^ for O_3_ in the peak season, equivalent to 31 parts per billion (ppb) at 298 K and 1,013 hPa. Severe O_3_ pollution has also been observed in other seasons and regions (26, 27). In addition, over the past 70 years, China's annual mean temperature has increased by an average of 0.26°C/decade, outpacing the global average of 0.15°C/decade (28).

There are several ways in which O_3_ and PM_2.5_ are expected to damage agricultural productivity. A large body of observational and experimental studies demonstrates that the two pollutants cause damage to terrestrial vegetation, by adversely affecting crop yields, forests, and grasslands (12, 15, 17, 18, 29). As a strong oxidant, O_3_ harms crops by entering leaves via stomata and reacting with compounds in the exposed wet cell-wall surfaces, generating harmful radicals that accelerate plant aging (15, 30). On the other hand, PM_2.5_ hinders crop growth by reducing solar radiation reaching the earth's surface (12, 17). Notably, aerosols like PM_2.5_ may increase crop productivity by scattering solar radiation, thus increasing the efficiency of photosynthesis (31). High O_3_ and PM_2.5_ concentrations may reduce the productivity in the livestock sector directly by damaging respiratory systems of livestock animals, similar to their effects on human health, and indirectly by causing damage to vegetation, food supplies, and ecosystem for livestock species that rely upon grasslands (32). Furthermore, the medical literature finds that exposure to high levels of O_3_ and PM_2.5_ is strongly associated with increased health and mortality risks in humans (33–35). Research has found robust evidence supporting negative impacts of elevated O_3_ and PM_2.5_ levels on worker productivity (36, 37).

In this article, we first estimate a panel regression model to analyze the sensitivity of agricultural TFP to O_3_, PM_2.5_, and temperature extremes at the county level for the years 2002–2015, controlling for other weather variables, technological change, and unobserved time-invariant location-specific factors (e.g. soils, geography). This sample period is determined by the availability of historical data on both agricultural TFP and O_3_ concentrations. Over this period, China has also transitioned from a modest food exporter to the world's largest importer. We next use the model to predict TFP under two conditions: (i) using historical, observed O_3_, PM_2.5_, and days with high temperatures exceeding 35°C for each year between 2002 and 2015, and (ii) hypothetical scenarios assuming that each of these factors had been kept at their 2002 levels. The percentage differences in predicted TFPs between the two conditions were subsequently used to estimate the relative importance of O_3_, PM_2.5_, and high temperatures in driving TFP variation over the sample period.

Our analysis addresses two significant challenges. First, nationwide ground-based O_3_ and PM_2.5_ monitoring data before 2013 are not available in China. While several studies have used satellite-driven models to generate high-resolution and long-term PM_2.5_ estimates, the corresponding estimates for surface O_3_ concentrations are very limited. Thus, national-scale studies focusing on the health effects of O_3_ exposure are mostly restricted to the post-2013 period (38). Second, air pollution concentrations are not randomly distributed across regions and the agricultural sector is a major source of air pollution. Agricultural operations such as cultivation, planting, weeding, mowing, and harvesting, which rely heavily on machinery and fuels, significantly contribute to particulate emissions (39). Emissions from livestock production also have the potential to form O_3_ and PM_2.5_ (40). Thus, the estimates based on ordinary least square (OLS) regressions are biased because of the reverse causality between agricultural production and pollution concentrations. We deal with this head on using an instrumental variable (IV) strategy.

We tackle the data challenge by employing machine learning techniques to generate the estimates of surface O_3_ concentrations for the period 2002–2019. This approach involves utilizing satellite-based pollution data at a spatial resolution of 45 km × 55 km, combined with recorded surface O_3_ concentrations, meteorological, geographical, and socioeconomic factors in the post-2013 period, to build relationships between surface O_3_ concentrations and these predictors. Assuming that these relationships remain stable across a given time period and utilizing the long-term satellite-based pollution data, we predict surface O_3_ concentrations before 2013. We then aggregate gridded O_3_ data to the county level to match up with the county-level agricultural TFP estimates for the regression analysis. We address the endogeneity of O_3_ and PM_2.5_ by using an IV approach that relies on changes in local wind direction as exogenous shocks to local pollution levels (41). Prior research has demonstrated that wind can affect pollution concentrations either by reallocating pollution produced from local sources (e.g. power plants or traffic) or by transporting external pollution generated in upwind regions into the county (41, 42). This approach generates a large number of instruments and therefore allows us to separately identify the causal effects of each pollution variable.

## Results

### Performance of machine learning models

**Fig. 1.**
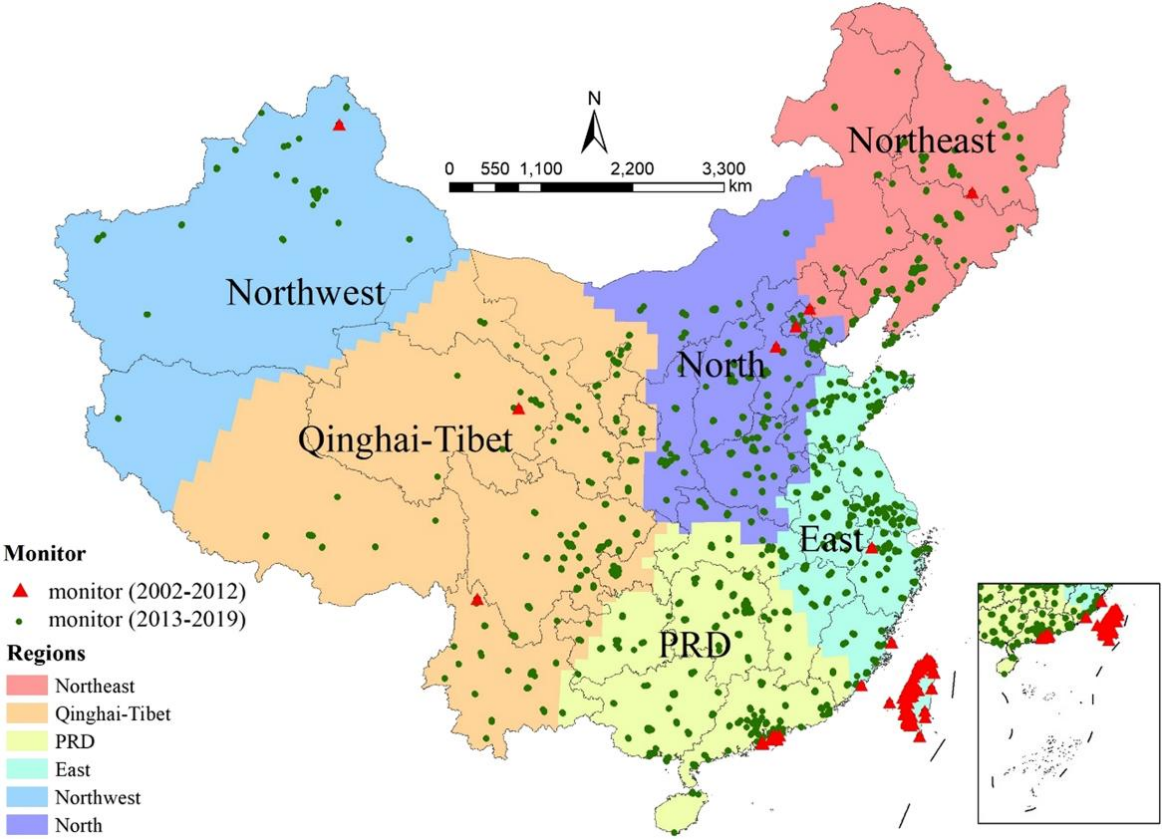
Spatial distribution of ground ozone monitoring stations. The solid dots are ground monitoring stations from the CNEMC network during the 2013–2019 period, while the triangles show observation sites for historical O_3_ measurements during the 2002–2012 period. PRD, Pearl River Delta region.

Figure 1 shows our study domain. We employed three machine learning algorithms, including Light Gradient Boosted Machine (LightGBM), eXtreme Gradient Boosting (XGBoost), and Super Learner, to generate monthly mean surface O_3_ concentrations. The details of these machine learning models and the predictor variables incorporated are described in Materials and methods.

**Fig. 2.**
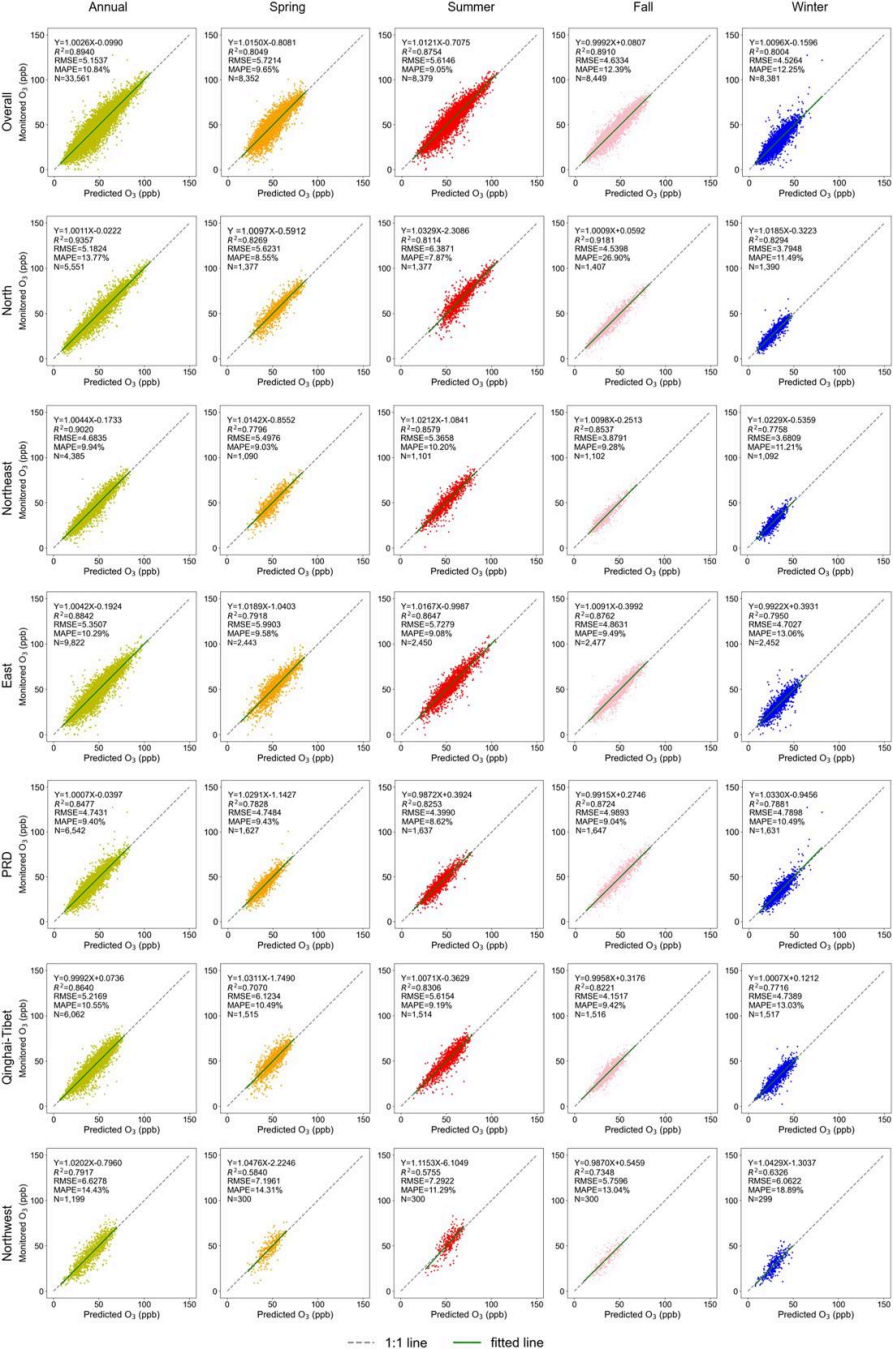
Cross-validation performance of the Super Leaner model across seasons in six subregions of China at the monthly level. These figures show density scatter plots of the monthly predicted MDA8 O_3_ levels vs. monitored levels from 2013 to 2019. RMSE, root-mean-squared prediction error; MAPE, mean absolute percentage error; PRD, Pearl River Delta region. Figures S1 and S2 show the performance of the LightGBM and XGBoost models.

Figure 2 shows the cross-validation performance of the Super Learner model across different seasons in six regions of China, which were created using a *k*-means cluster algorithm (Materials and methods). The key parameters characterizing model performance include cross-validated (CV) *R*^2^, the root-mean-squared-error (RMSE), and mean absolute percentage error (MAPE), which were obtained by training these machine learning models separately for each of the six regions and across seasons using historical data in 2013–2019. Our models exhibit high fidelity in predicting the surface O_3_ concentrations in all regions, indicated by the fitted relationship between the predicted monthly mean MDA8 O_3_ and the corresponding ground measurements being nearly coincident with the 1:1 line. Nationally, the random 10-fold CV *R*^2^ is 0.89, with an RMSE of 5.2 ppb and a MAPE of only 10.8%. Across seasons, the model performed best in summer and fall, with a CV *R*^2^ = 0.88–0.89, an RMSE of 4.6–5.6 ppb, and a MAPE of 9.1–12.4%. Performance in other seasons is slightly lower (*R*^2^ = 0.80, RMSE = 4.5–5.7 ppb, and MAPE = 9.7–12.3%). Regionally, the Super Learner model performed best in the North China region, with a CV *R*^2^ = 0.94, an RMSE of 5.2 ppb and a MAPE of 13.8% when trained using year-around observations, and relatively poorly in the northwest region (*R*^2^ = 0.79, RMSE = 6.6 ppb, and MAPE = 14.4%). The slightly poor performance in northwest is primarily due to the sparse meteorological and air monitoring stations in the area, resulting in insufficient observations for model training. The LightGBM and XGBoost models also performed well across all regions and exhibited similar predictive accuracy (Figs. S1 and S2).

To evaluate the predictive capability of our models prior to 2013, we collected historical ground-based O_3_ measurements in 2002–2012 from 100 ozone observation sites located in mainland China, Hong Kong, Macao, and Taiwan (depicted as red triangles in Fig. 1, Table S1). We used these trained machine learning models to predict monthly mean MDA8 O_3_ concentrations for these observation sites. We found that, at the national level, the predicted O_3_ concentrations are in moderate agreement with recorded historical O_3_ concentrations at the monthly level, with a CV *R*^2^ of 0.60, an RMSE of 8.9 ppb, and a MAPE of 16.6–16.9%. The predictive accuracy is significantly higher in major agricultural production regions (i.e. East China with a CV *R*^2^ = 0.70, an RMSE = 6.5–6.6 ppb, and a MAPE = 11.0–11.3%, Table S2).

### Spatiotemporal trends of surface O_3_ concentrations

**Fig. 3.**
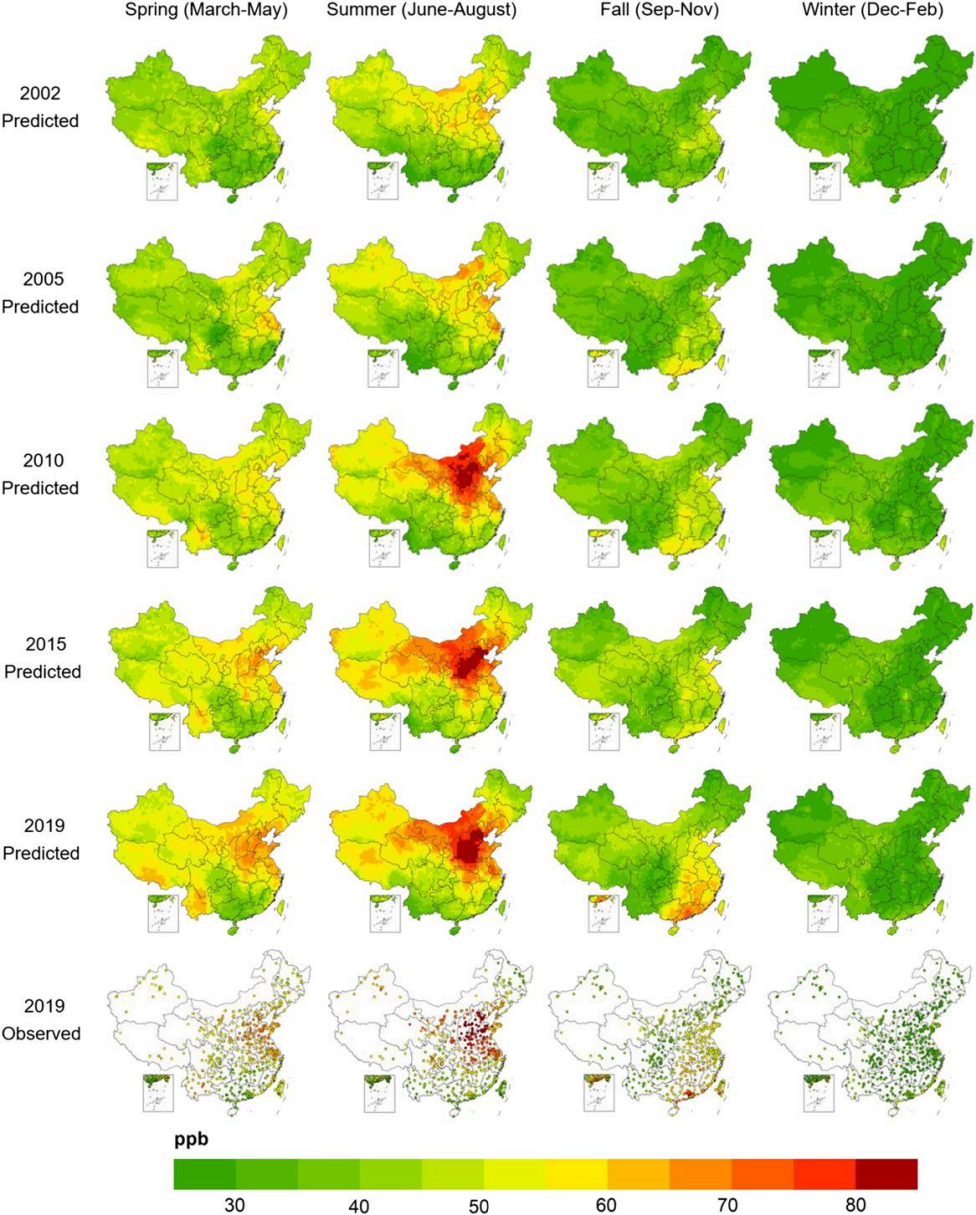
Spatial distribution of monthly mean MDA8 O_3_ concentrations in China from 2002 to 2019 at 45 km × 55 km spatial resolution. Maps in top five rows show O_3_ estimates based on the Super Learner model. Maps in the bottom row show observed O_3_ levels in 2019.

While all three machine learning models demonstrated similar performance (Figs. S3 and S4), the Super Learner model exhibited a slight advantage. Hence, we used predictions from the Super Learner model as our preferred O_3_ estimates. Figure 3 shows the spatial and temporal distributions of monthly mean MDA8 O_3_ concentrations estimated by the Super Learner model at a spatial resolution of 45 km × 55 km in 5 years, 2002, 2005, 2010, 2015, and 2019. Temporally, annual mean MDA8 O_3_ concentrations across China increased from 38.1 ppb in 2002 to 47.8 ppb in 2019, with substantial variations across seasons and regions (Fig. S5). Surface O_3_ levels peaked during the summer, with the highest concentrations occurring in the North China Plain. Winter was the only season with the lowest O_3_ levels (Table S3).

China's summer O_3_ pollution intensified over the 18-year period. In 2002, only a few regions in northern China had summer mean MDA8 O_3_ concentrations above 60 ppb. However, O_3_ pollution grew increasingly severe in other regions. Since 2010, most of the areas in northern China have experienced severe summer O_3_ pollution. In Beijing–Tianjin–Hebei (BTH), Henan, and Shanxi, monthly mean summer MDA8 O_3_ concentrations have been higher than 70 ppb (Fig. S6), substantially exceeding the WHO air quality guidelines for the peak season O_3_ level of 31 ppb (43).

Intense O_3_ pollution has also been observed in the spring and fall seasons during this time span. In 2019, the BTH and Shandong province recorded monthly mean MDA8 O_3_ concentrations in spring above 64 ppb. Meanwhile, a few counties in Yunnan province, located in Southwest China, saw a spring mean MDA8 O_3_ level exceeding 66 ppb. High O_3_ pollution also occurred during the fall in eastern China and the Pearl River Delta (PRD) region, with average monthly mean MDA8 O_3_ concentrations in both regions reaching 56 ppb in 2019.

### Responses of agricultural TFP to pollution and temperature extremes

We employed several approaches to estimating county-level agricultural TFP, which represents the growth in aggregated agricultural output from all subsectors (cropping, livestock, forestry, and fisheries) that is not accounted for by changes in primary inputs (such as land, labor, fertilizer, and agricultural machinery). More technical details can be found in Materials and methods. Consistent with prior studies (20, 24), we observed a leveling-off of agricultural TFP in China between 2002 and 2015, with considerable variation across counties and years (Fig. S7).

Because agricultural TFP measures the efficiency of all agricultural production activities over the year, our baseline analysis used annual mean MDA8 O_3_ and PM_2.5_ concentrations as pollution controls. Given the sensitivity of agricultural TFP to weather and the strong correlation between air pollutant concentrations and weather conditions, our regression analyses also control for a flexible set of weather variables, including the number of days with daily temperatures falling into specific ranges, linear and quadratic terms of cumulative precipitation, sunshine duration, average relative humidity, air pressure, and wind speed, as well as technological changes, geographical, and other location-specific unobserved factors. We tested the robustness of our results using alternative pollution and temperature measures.

Table 1 reports the estimated impacts of pollution and temperature on agricultural TFP. The OLS estimates reported in column 1 in panel A suggest that the increases in the annual mean MDA8 O_3_ and PM_2.5_ concentrations and high temperatures above 35°C were negatively correlated with agricultural TFP derived from the Translog conventional production function without constant returns to scale (TL-CPF) model (Materials and methods). However, these estimates are subject to a range of biases, because (i) pollution was not randomly distributed across regions; (ii) pollution data might be subject to measurement error; and (iii) there may exist reverse causality between agricultural production and pollution concentrations. We deal with these sources of endogeneity head on by adopting a classic IV strategy, as discussed below.

**Table 1.** The effects of pollution and temperature extremes on agricultural productivity.

Dependent variable	Log (Agricultural productivity)					
	(1)	(2)	(3)	(4)	(5)	(6)
	OLS	IV	IV	IV	IV	IV
	TL-CPF	TL-CPF	TL-CPF-w/CRS	CD-CPF	CD-SFA-w/CRS	Labor productivity
Panel A: Productivity responses to annual mean MDA8 O_3_						
Annual MDA8 O_3_	−0.0008	−0.0224^a^	−0.0219^a^	−0.0199^a^	−0.0210^a^	−0.0250^a^
	(0.0027)	(0.0070)	(0.0070)	(0.0069)	(0.0069)	(0.0084)
PM_2.5_	−0.0039^a^	−0.0092^a^	−0.0102^a^	−0.0087^a^	−0.0087^a^	−0.0058
	(0.0008)	(0.0033)	(0.0032)	(0.0031)	(0.0030)	(0.0038)
≥35°C	−0.0032^c^	−0.0050^b^	−0.0053^a^	−0.0053^a^	−0.0055^a^	−0.0054^b^
	(0.0018)	(0.0020)	(0.0020)	(0.0020)	(0.0020)	(0.0026)
F-test (KP statistics)	—	12.4088	12.4088	12.4088	12.4088	12.4088
Observations	26,788	26,788	26,788	26,788	26,788	26,788
Panel B: Productivity responses to winter and nonwinter mean MDA8 O_3_						
Winter MDA8 O_3_	−0.0027^c^	0.0051	0.0016	0.0028	0.0043	0.0127
	(0.0014)	(0.0088)	(0.0087)	(0.0086)	(0.0087)	(0.0103)
Nonwinter MDA8 O_3_	0.0005	−0.0208^a^	−0.0191^a^	−0.0178^a^	−0.0194^a^	−0.0259^a^
	(0.0023)	(0.0055)	(0.0055)	(0.0054)	(0.0053)	(0.0068)
PM_2.5_	−0.0032^c^	−0.0092^a^	−0.0102^a^	−0.0087^a^	−0.0087^a^	−0.0060
	(0.0018)	(0.0033)	(0.0032)	(0.0031)	(0.0030)	(0.0038)
≥35°C	−0.0032^c^	−0.0050^b^	−0.0054^a^	−0.0054^a^	−0.0056^a^	−0.0055^b^
	(0.0018)	(0.0020)	(0.0020)	(0.0020)	(0.0019)	(0.0026)
F-test (KP statistics)	—	10.9139	10.9139	10.9139	10.9139	10.9139
Observations	26,788	26,788	26,788	26,788	26,788	26,788

This table shows estimated coefficients of pollution and high temperatures on agricultural productivity. The dependent variables are the natural log of agricultural TFP derived from the TL-CPF model (columns 1 and 2), the TL-CPF-w/CRS model (column 3), the CD-CPF model (column 4), the CD-SFA-w/CRS model (column 5), and labor productivity (defined as the output per agricultural worker in column 6). Column 1 reports the OLS estimates. Columns 2–6 report the estimated coefficients from the IV design. All regressions include the number of days with daily temperatures falling into specific bins at a width of 5°C, as well as linear and quadratic terms of cumulative precipitation, sunshine duration, average relative humidity, air pressure, and wind speed as weather controls. The symbol “≥35°C” denotes the number of days with daily temperatures exceeding 35°C. All regressions include county fixed effects and year fixed effects. Standard errors (in parentheses) are clustered at county level. Significance: ^a^*P* < 0.01, ^b^*P* < 0.05, ^c^*P* < 0.1.

Hence, column 2 presents the IV estimates of the causal effects of O_3_ and PM_2.5_ on agricultural TFP, with the two pollution variables instrumented by wind direction (Eq. 2 in Materials and methods). The first stage Kleibergen–Paap *F*-statistic is >10. The estimated O_3_ and PM_2.5_ coefficients are negative and statistically significant (*P* < 0.01). The IV estimate implies that each 1 ppb increase in the annual mean MDA8 O_3_ concentrations was associated with a 2.24% reduction in agricultural TFP. In comparison, the estimated PM_2.5_ impact is smaller. Holding all else equal, each 1 μg/m^3^ increase in PM_2.5_ concentrations was correlated with a 0.92% reduction in TFP. It is worth noting that the estimated coefficient for PM_2.5_ may also reflect the effects of aerosols like PM_10_ that are highly correlated with PM_2.5_. This suggests that the interpretation regarding the impact of PM_2.5_ should be made cautiously, as it may represent some of the broader effect of aerosol pollution on agricultural productivity. Each additional day of exposure to temperatures above 35°C during a year is estimated to depress TFP by 0.5%. Columns 3–5 confirm the robustness of these findings when agricultural TFP was estimated with alternative approaches. The difference between OLS and IV estimates underlines the importance of addressing the endogeneity of pollution variables using the IV approach.

Column 6 reports the corresponding impacts on labor productivity, defined as the output value per agricultural worker. Although labor productivity is a partial productivity measure, this exercise helps to identify the underlying mechanisms through which pollution and temperature extremes affect agricultural TFP. Labor productivity was strongly influenced by elevated O_3_ pollution and exposure to high temperatures above 35°C, while the impact from PM_2.5_ was negative but statistically insignificant. These point estimates align with those reported in columns 2–5 using TFP to measure productivity. These results suggest that reduced labor productivity is one of the possible channels by which pollution and extreme temperatures negatively affected TFP. Coefficients for other weather variables are reported in Table S4. For example, holding all else equal, each 1-h increase in total sunshine hours was associated with a 0.04–0.07% increase in agricultural TFP. Other weather variables have weak statistical significance. That said, it is of key importance to include these in the regression to avoid omitted variables bias concerns.

Our findings are robust to alternative measures of exposure to O_3_. The annual mean MDA8 O_3_ measure used in our main specification assigns equal weight to monthly O_3_ observations throughout the year, potentially underestimating the true impact of O_3_ exposure on TFP, given that major agricultural production activities contributing to TFP predominantly occur in summer. To address this, we considered three well-established cumulative O_3_ indices: W126, AOT40, and SUM06. The W126, proposed by the U.S. Environmental Protection Agency (EPA), is the sum of hourly concentrations weighted by a sigmoidal function, placing greater emphasis on higher concentrations. AOT40 and SUM06 aggregate the sum of hourly O_3_ concentrations exceeding 40 ppb and 60 ppb, respectively (Materials and methods). All three metrics give more weight to higher O_3_ values, capturing the specific months–most notably the summer months—that exert a significant detrimental impact on agricultural TFP. These indices have been widely adopted in previous studies estimating O_3_-crop yield relationships (15). We calculated annual values of the three cumulative indices to examine the sensitivity of our results. We found that all three indices were negatively correlated with agricultural TFP (*P* < 0.01) and that estimated impacts from PM_2.5_ and high temperatures are consistent with our baseline results (panel A of Table S5).

### Winter vs. nonwinter O_3_ impacts

Motivated by the fact that winter was the only season without severe O_3_ pollution in China (Fig. 3), we further estimated a model that includes the average MDA8 O_3_ concentrations during winter and nonwinter seasons as two separate O_3_ variables. The IV estimates of the O_3_ impacts in Panel B of Table 1 indicated that each 1 ppb increase in the average MDA8 O_3_ concentrations during the nonwinter seasons was associated with a 1.78–2.08% reduction in agricultural TFP (*P* < 0.01). These estimates are close to the estimated impacts of annual mean MDA8 O_3_ on productivity. In contrast, we found a null effect of winter O_3_ on TFP. This indicates that elevated O_3_ concentrations during the nonwinter seasons were the key driver behind the decline in agricultural TFP. The estimated coefficients of the PM_2.5_ and weather variables are nearly unchanged (Table S6). These findings are also robust when using labor productivity as the dependent variable, or using W126, AOT40, and SUM06 as alternative O_3_ measures (panel B of Table S5).

### Robustness checks

As is standard in the impacts literature, we conducted a series of robustness checks of our findings to alternative specifications, IV, estimation strategies, and data treatments. Specifically, we considered different clustering choices to account for spatial and temporal correlations in error terms (Table S7). We changed specifications by using different types of fixed effects, time trends, and weather controls (Table S8). We allowed instruments to vary with the size of wind angle bins and the number of county groups, and estimated the model using the limited information maximum likelihood estimator to make sure that our estimates do not suffer from weak instrument bias (Table S9). We also re-estimated the model by removing possible outliers (Table S10). Given the limited evidence of pollution affecting fisheries, we excluded coastal counties with a significant dependence on fisheries from our primary sample (Table S11). Moreover, we excluded the PM_2.5_ variable from the regression models to examine whether the estimated impacts of O_3_ on agricultural productivity are sensitive to the removal of this pollution covariate (Table S12). The main conclusions drawn above survive all of these robustness analyses.

Furthermore, we conducted two placebo checks to ensure that the estimated relationship between pollution and TFP did not arise by chance. We first estimated models using 1,000 datasets that were generated by randomly mismatching the county-year TFP and pollution data. We then generated additional 1,000 datasets where TFP and pollution data were randomized within seasons and regions. Our baseline estimate falls outside of the resulting distributions of the estimates derived from these placebo datasets (Fig. S8), demonstrating that the estimated relationship between TFP and pollution is unlikely to be spurious.

### Regional heterogeneity

The sensitivity of TFP to O_3_ pollution may vary across regions due to differences in agricultural production systems and, as a result, TFP composition. To explore this, we divided our sample counties into four agricultural divisions: the Northeast and North China Plain, the Northwest Region, the Southwest Region, and the South and Yangtze River Region, according to the “Sustainable Agricultural Development Planning” released by China's Ministry of Agriculture and Rural Affairs (MARA). These divisions capture the regional heterogeneity in agricultural production patterns across China.

Our findings show that TFP sensitivity to O_3_ is regionally heterogeneous. Specifically, exposure to rising O_3_ levels was associated with lower productivity in the Northeast and North China Plain as well as the South and Yangtze River Region (*P* < 0.05), regions traditionally recognized for substantial grain production. The effects largely remain statistically insignificant in other regions (*P* > 0.1) (Table S13). Moreover, by using MARA's list of regions designated as major grain- or livestock- producing regions, we found that the negative impact of O_3_ on agricultural productivity was statistically significant in major grain-producing regions (*P* < 0.05), yet remains insignificant in major livestock-producing regions (*P* > 0.1).

### Responses of crop and livestock yields to O_3_ pollution

Given the pronounced adverse effects of O_3_ on agricultural productivity, identifying the origins of agricultural TFP's sensitivities to rising O_3_ levels is vital for effective policy design. Ideally, a thorough analysis would entail estimating TFP for each agricultural subsector. However, this is not plausible due to the limited availability of sector-specific input data. As an alternative, we examined the yield responses of major crop and livestock commodities to elevated O_3_ levels. For the crop sector, we focused on the five most widely planted crops in China: maize, soybean, rice, wheat, and tubers. For the livestock sector, the only available productivity measure in our dataset is milk production per cow. We performed separate regressions using cumulative O_3_ indices, constructed during the growing seasons of crop or livestock products. This exercise helps to illuminate whether sensitivities of TFP to O_3_ pollution originate from the crop sector or the livestock sector.

The regression results indicate that O_3_ pollution has negatively affected yields of maize, single-season rice, wheat, and tubers (Tables S14–S16). In contrast, the O_3_ impact on milk yield was statistically insignificant. Taken together with the fact that O_3_ significantly reduced TFP in major grain-producing regions, these findings suggest that the adverse effect of elevated O_3_ pollution on agricultural TFP likely arises mainly from the crop sector's vulnerability to O_3_ concentrations.

### Historical productivity losses due to exposure to pollution and temperature extremes in 2002–2015

To contextualize our regression analysis and determine which factor accounted for significant variation in historical agricultural TFP, we used the baseline estimates from column 2, panel B of Table 1 to predict county-level TFP under two conditions (i) using historical, observed O_3_, PM_2.5_, and days with high temperatures above 35°C for each year between 2002 and 2015, and (ii) hypothetical scenarios with each of these factors held at their 2002 levels. We then calculated the percentage changes in county-level TFP between the two conditions, which were weighted by total agricultural output value and summed to derive national-level TFP impacts of recent pollution and temperature trends. We note that the first stage of our IV models can accurately predict surface O_3_ and PM_2.5_ concentrations (Fig. S9).

**Fig. 4.**
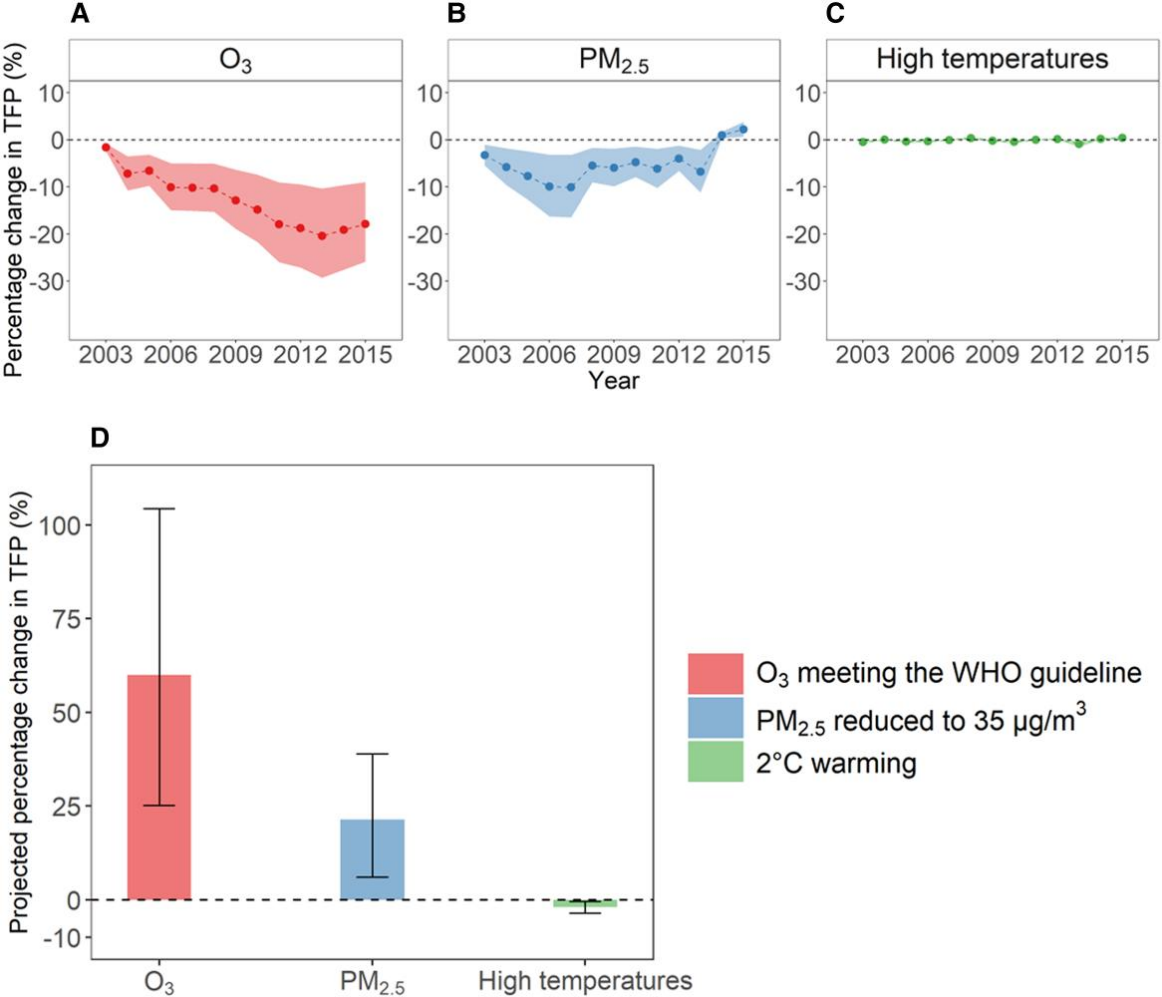
Estimated agricultural productivity changes due to pollution and high temperatures. A–C) Estimated changes in TFP resulting from variations in nonwinter O_3_ concentrations, PM_2.5_, and days with high temperatures above 35°C, respectively, for the years 2003–2015. Productivity changes were calculated by using Eq. (1) to predict TFP under two conditions: (i) using historical, observed values of O_3_, PM_2.5_, and days with high temperatures above 35°C for each year between 2002 and 2015, and (ii) hypothetical scenarios with each of these factors held at their 2002 levels. Each point is a weighted mean of percentage changes in county-level TFP between the two conditions, where the value of a county was weighted by its total output value. The black, dashed, horizontal line marks 0 change for reference. The shallow bands in each panel are 95% confidence intervals. D) The projected changes in agricultural TFP from hypothetical pollution reductions and a scenario of 2°C warming, in which daily temperatures across all counties would uniformly increase by 2°C relative to the 2015 levels. The length of a bar shows the projected percentage change due to a given factor relative to 2015, and the whiskers are 95% confidence intervals of the estimates.

Estimates of historical agricultural TFP loss due to rising O_3_ concentrations over the 2002–2015 period increased rapidly from 1.6% in 2003 to 20.4% in 2013 (Fig. 4A). We found that TFP was 17.9% lower in 2015 than it would have been if O_3_ pollution was kept at the 2002 level. In comparison, TFP loss due to PM_2.5_ was smaller, ranging from 3.3 to 10.1% in 2003–2013 (Fig. 4B). Due to reduced PM_2.5_ pollution since 2013 (25), agricultural TFP increased by ∼1.0% in 2014 and 2.2% in 2015. The percentage changes in TFP due to high temperatures above 35°C fluctuated between −0.9% and +0.4% over the sample period (Fig. 4C). Results remained similar when agricultural TFP was estimated with alternative approaches (Fig. S10).

Regionally, rising ambient O_3_ levels reduced agricultural TFP in nearly all regions of China in 2015, with the largest TFP loss (about 38%) occurring in the north China Plain (Fig. S11). Because PM_2.5_ concentrations have begun to decline since 2013 (25) and the estimated impact of PM_2.5_ on TFP is relatively small, PM_2.5_-induced TFP loss was small, with most significant losses occurring in the northeast regions of China (4.4%). The percentage changes in agricultural TFP due to high temperatures were generally <5% in all regions, which is consistent with estimates found in the literature focusing on the detected impacts of climate change in China (20).

### Projected productivity gains from pollution reductions

The substantial reduction in agricultural productivity since 2002 due to O_3_ implies that more stringent and comprehensive air quality regulation policy that encompasses other pollutants besides PM_2.5_ can produce further benefits for agricultural productivity in China. Figure 4D shows that, holding all else constant, national average agricultural TFP would increase by 60% if surface O_3_ concentrations during the nonwinter seasons met the WHO air quality guidelines for peak-season O_3_ exposure, which requires a 40% reduction in national average O_3_ concentrations compared to the 2015 level. National average TFP would increase by 21% if PM_2.5_ concentrations were to reach the “Beautiful China” strategy that aims to reduce PM_2.5_ levels to 35 μg/m^3^ by 2035. The estimates of productivity gains due to pollution reductions range from 36 to 70% for O_3_ and from 20 to 24% for PM_2.5_, depending on the methods used to compute TFP and generate surface O_3_ estimates (Table S17). Taken together, simultaneously reducing O_3_ and PM_2.5_ would lead to a significant increase in agricultural TFP. These productivity gains have the potential to counter expected productivity losses (∼2%) from a scenario of 2°C warming. In this simple scenario, daily temperatures of all Chinese counties are assumed to uniformly rise by 2°C relative to the 2015 levels. This rightward shift of 2°C in the daily temperature distribution would lead to an increased frequency of temperature extremes.

## Discussion

### Conclusions

Using machine learning methods, this analysis first estimates fine-scale monthly ground-level MDA8 O_3_ concentrations from 2002 to 2019 in China. These estimates were subsequently used in econometric models to analyze the impacts of two major air pollutants, namely O_3_ and PM_2.5_, alongside high temperatures on agricultural productivity. We present four major findings. First, China's surface O_3_ pollution deteriorated spatially and temporally over the 18-year period, with severe O_3_ pollution occurring during summer and in northern China. Heavy O_3_ pollution also occurred in the spring and fall seasons as well as in other regions, such as PRD, Southwest and eastern China. Second, China's agricultural productivity exhibited strong negative responses to rising surface O_3_ levels during the nonwinter seasons, and this negative impact increased with higher levels of O_3_ pollution (Table S18). Third, O_3_ pollution adversely impacted the yields of major crops and was associated with a decline in agricultural labor productivity. Given that China's crop sector is more labor intensive than its livestock sector, this implies that the sensitivity of China's agricultural TFP to O_3_ pollution may have predominantly originated from the crop sector. Lastly, the productivity loss due to elevated O_3_ levels increased nearly linearly over time from 1.6 to 20.4% across the 2002–2015 period, far exceeding the corresponding losses from PM_2.5_ and extreme temperatures.

We further projected the potential gains in agricultural productivity from hypothetical pollution reductions. The results show that, holding all else fixed, national average agricultural productivity would increase by 60% relative to its level in 2015, if surface O_3_ concentrations meet the WHO air quality guidelines for the peak-season O_3_ concentrations, or by 21% if PM_2.5_ concentrations are reduced to 35 μg/m^3^. These productivity gains from pollution reductions can offset the projected productivity loss due to a simulated 2°C rise in temperature in the future. Our findings demonstrate that meeting the WHO air quality guidelines, which are primarily designed to protect human health, would also yield significant cobenefits in terms of enhanced agricultural productivity.

The existing literature mainly examined the direct effect of O_3_ pollution on crop yields, which is just one aspect of agricultural production efficiency. Our research, on the other hand, adopts a broader approach by considering the impacts on overall agricultural production efficiency and labor productivity. Our analysis provides a more comprehensive understanding of how air pollution affects agricultural TFP and identifies reduced labor productivity as an important driving factor. It also highlights the need for strategies to mitigate the adverse impacts of air pollution on agricultural productivity, beyond just addressing crop yield losses.

### Comparisons with existing studies

The absence of reliable pollution monitoring data prior to 2013 has stimulated a rapidly growing body of research employing machine learning models combined with satellite remote sensing data to estimate ground-level pollution concentrations in China. Many studies have predicted spatiotemporal patterns of PM_2.5_ concentrations across China for the historical period before 2013 (see review in Liang et al. (44)). Several recent studies have developed machine learning models to predict MDA8 O_3_ concentrations; however, these studies are limited in terms of their spatial or temporal coverage and machine learning approaches. For example, most of these studies focused only on small sets of Chinese regions (45–48). The literature contains only a few nationwide studies estimating historical MDA8 O_3_ concentrations in China. Liu et al. (49) predicted ambient O_3_ concentrations from 2005 to 2017 using the XGBoost algorithm at a spatial resolution of 0.1° × 0.1° (monthly CV *R*^2^ = 0.90, RMSE = 5.7 ppb). Zhan et al. (50) simulated O_3_ levels in 2015 using the random forest algorithm at a resolution of 0.1° × 0.1° (monthly CV *R*^2^ = 0.71, RMSE = 9.7 ppb). Using an iterative random forest model, Chen et al. (51) estimated surface O_3_ concentrations from 2008 to 2019 at 0.1° × 0.0625° resolution (CV *R*^2^ = 0.79, RMSE = 11.0 ppb). A key limitation of these national studies is that they all applied one single machine learning algorithm without demonstrating the robustness of their estimates to alternative algorithms.

Our research contributes to the literature on estimating surface O_3_ concentrations in China in two major aspects. First, we employed three machine learning algorithms (namely LightGBM, XGBoost, and Super Learner) and provided O_3_ estimates for a relatively longer time span (2002–2019). The three machine learning models that we adopted have demonstrated higher prediction accuracy, computational efficiency, and reduced possibility of over-fitting relative to the random forest algorithm employed by other national studies (52). Second, in contrast to studies considering China as a whole, we trained the machine learning models separately for each of the six subregions and reported model performance, which has greatly enhanced the credibility of our machine learning models. Our models exhibited comparable performance to Liu et al. (49) and outperformed other nationwide studies. Our models also outperformed many chemical transport model simulations (53, 54), whose applications are often constrained due to coarse spatial resolutions and high computational costs (55). Our estimates of spatiotemporal trends of surface O_3_ concentrations were in agreement with existing studies (49).

While the sensitivity of agricultural TFP to pollution remains poorly understood, several studies have examined how temperature shocks affected agricultural TFP (11, 13, 14, 20). Focusing on China's agriculture, Chen and Gong (20) found that one additional day with exposure to temperatures above 33°C was associated with a reduction of 2.6% in agricultural TFP over the 1980–2015 period, which is larger than our estimate (0.5%). In their study, agricultural output per unit of land, defined as a county's aggregated agricultural value of outputs divided by the total acreage of arable land in this county, was used to compute agricultural TFP. To reconcile our estimate with theirs, we replicated their analysis by using the same specification, TFP calculation, and sample from 2002 to 2015. The results showed that TFP declined by only 0.1% (Table S19) for each additional day with temperatures above 33°C, which is broadly consistent with our estimate. The decline in temperature sensitivity is due to the significant improvement in China's agricultural resilience to climate shocks since the 1990s, primarily because of the rapid expansion of irrigation infrastructure in the country (56).

Our findings of large and detrimental impacts of O_3_ pollution on crop yields are in agreement with the estimates reported in the literature, which have investigated the combined impacts of climate change and air pollution on crop yields in other countries. For example, Burney and Ramanathan (17) found that over 90% of the yield changes for wheat and rice in India during the 1980–2010 period could be attributed to air pollution (e.g. black carbon and O_3_). Auffhammer et al. (57) concluded that brown clouds were a key driver reducing Indian rice harvests. Using a global vegetation and crop model, Schauberger et al. (58) estimated that historical yield losses due to O_3_ pollution amounted to ∼6% for soybeans and 34% for wheat in China from 2008 to 2010. Furthermore, estimates based on exposure-response functions indicated that exposure to O_3_ pollution led to relative yield losses of 33, 23, and 9% for wheat, rice, and maize, respectively, in China (59). Our analysis extends these findings by showing that, in addition to maize, wheat, and rice, rising O_3_ pollution correlated with lower tuberous root yields.

### Uncertainty and limitations

We performed several uncertainty analyses to examine the robustness of our predicted O_3_ estimates and their impact on agricultural productivity. The results show that the model performance and the predicted spatial and temporal patterns of O_3_ concentrations remain robust to variations in predictor variables and data (Fig. S12). The estimated impacts of pollution and temperature extremes were also in agreement with the baseline results (Fig. S13).

Several caveats should be applied to our analysis. First, despite our efforts to compile and utilize all available historical observation data to validate O_3_ predictions, our machine learning models did not perform equally well in all regions of China, with slightly poorer performance in northwestern China due to the scarcity of meteorological and air monitoring stations. Second, uncertainties may be introduced when constructing cumulative indices of O_3_. In the absence of estimates of hourly O_3_ concentrations, we made simplifying assumptions when calculating AOT40, SUM06, and W126 indices: (i) the hourly O_3_ concentrations during the peak 8 h (or during the nonpeak hours) each day in a month are the same and the O_3_ concentrations during the peak hours are equal to the predicted monthly mean MDA8 ozone concentrations over the 2002–2015 period; (ii) the ratio of mean O_3_ concentrations during the peak 8 h to that during the nonpeak hours, though differing by month and by region, remained stable over the 2002–2019 period. We computed this ratio using the observed hourly data over the 2013–2019 period and then estimated the mean hourly O_3_ concentrations during the nonpeak hours for 2002–2015. We investigated the validity of these two assumptions by comparing cumulative O_3_ indices computed using the observed and estimated hourly data in 2013–2019. The results showed that the percentage differences in the sample means based on the two data sources were generally <11% (Table S20), suggesting that these assumptions are reasonable in our setting. However, to what extent these assumptions hold in years before 2013 cannot be examined. Third, our analysis may have underestimated O_3_ concentrations in rural China, as most of the ground-level O_3_ monitoring stations used in our analysis are located in urban areas, which typically have lower O_3_ levels than rural regions (59).

Our main analysis did not consider the impacts of other air pollutants. Recent studies found that agricultural production exhibited negative responses to SO_2_ and NO*_x_* (17), which were often emitted from the same pollution source and were thus highly correlated with PM_2.5_ and O_3_ concentrations (Table S21). While it is possible to generate SO_2_ and NO*_x_* estimates using similar machine learning models, the lack of historical data for these pollutants before 2013 restricted our ability to evaluate the predictive capability of these models prior to 2013. Nonetheless, we conducted additional robustness checks by progressively adding predicted values for SO_2_ and NO_2_, which were generated using the three machine learning models, as additional controls, even though these values were not validated against historical data. We found that these machine learning models performed well in predicting surface SO_2_ and NO_2_ concentrations (Tables S22 and S23). The regression results indicated that the estimates for PM_2.5_ and O_3_ were consistent with our main results (Table S24). This robustness check reinforces our main findings.

Our findings highlight the urgency of reducing O_3_ pollution to sustain China's agricultural productivity growth. Environmental policies need to incentivize research and investments to reduce NO*_x_* and volatile organic compounds (VOCs) emissions, the precursors of O_3_ pollution. The rapid rise of summer O_3_ pollution in the North China Plain calls for immediate action in order to reduce the adverse impacts of O_3_ pollution in this region given its important role in China's agriculture. Improved agricultural policies are also needed to guide research toward identifying the origins of sensitivity of agricultural productivity to air pollution and mitigating the associated negative impacts.

## Materials and methods

We used multiple data sources to estimate surface O_3_ concentrations during the period of 2002–2019, including a dataset of ground O_3_ measurements, high-resolution satellite-derived pollution data from the National Aeronautics and Space Administration (NASA), a meteorological dataset, and datasets containing other predictor variables for O_3_ estimation. Combined with the ground O_3_ estimates, we relied on a dataset of ground PM_2.5_ estimates and county-level agricultural TFP estimates to assess the impacts of pollution and temperature extremes on agricultural TFP.

### Ground O_3_ measurements

We obtained hourly O_3_ concentrations from 1,715 ground monitoring stations during the 2013–2019 period from the China National Environmental Monitoring Center (http://www.cnemc.cn/), Hong Kong Environmental Protection Department (https://www.epd.gov.hk/epd/english/top.html), Macao Environmental Protection Agency (https://www.dspa.gov.mo/index.aspx), and Taiwan Environmental Protection Administration (https://www.epa.gov.tw/) (Fig. 1). Based on these hourly O_3_ concentrations, we computed the MDA8 O_3_ concentrations and then aggregated them to the monthly mean, which were used for training of machine learning models and cross-validation.

To assess the predictive capability of the machine learning models for O_3_ concentrations before 2013, we collected historical O_3_ measurements during the 2002–2012 period from 100 O_3_ observation sites. The O_3_ concentrations at these sites were originally recorded at the hourly level, except for sites in Macao, where recordings were made at the daily maximum 8-h level. Initially, the O_3_ concentrations were reported in the unit of μg/m^3^ under the standard temperature and pressure conditions (273 K, 1,013 hPa). We converted these concentrations to parts per billion (ppb), adjusting for conditions at 298 K and 1,013 hPa, following the methodology outlined in Gelaro et al. (60). These recordings were then computed as the MDA8 O_3_ concentrations and aggregated to the monthly mean for validating historical O_3_ predictions from 2002 to 2012.

### Satellite-derived pollution data

We were aware of the availability of several satellite-based reanalysis products, and selected the MERRA-2, the latest version of global atmospheric reanalysis product developed by NASA. This product assimilates space-based observations of meteorological variables, aerosols, and O_3_ and incorporates their interactions with other physical processes in the climate system (60). MERRA-2 has been widely used by previous studies to estimate ground-level PM_2.5_ pollution (44, 61). The variables reported in the MERRA-2 datasets include O_3_ mixing ratio, air density, and surface mass concentrations of major aerosols components across the globe. The O_3_ mixing ratio and air density were extracted from the product MERRA-2 3-hourly Instantaneous Model (M2I3NVASM, https://disc.gsfc.nasa.gov/datasets/M2I3NVASM_5.12.4 and M2I3NVAER, https://disc.gsfc.nasa.gov/datasets/M2I3NVAER_5.12.4, respectively). The surface mass concentrations of major aerosols components were extracted from the product MERRA-2 1-hourly time-averaged model (M2T1NXAER, https://disc.gsfc.nasa.gov/datasets/M2T1NXAER_5.12.4). These satellite-based pollution data are reported at a spatial resolution of 0.5°×0.625° (∼45 km × 55 km). We extracted these grid-level pollution data for China between 2002 and 2019. We calculated the surface O_3_ concentration by multiplying the O_3_ mixing ratio (in kg kg^−1^) with the air density (in kg m^−3^). Major aerosol components reported by MERRA-2 include organic carbon, black carbon, dust, sulfate, and sea salt. We converted these hourly satellite-based pollution concentrations into the corresponding monthly means. The MERRA-2 data come with its own limits, including well-documented regional biases and aerosols components not validated by ground-based observations. To address these issues, we performed one uncertainty analysis using only ground-validated total PM_2.5_ as the pollution predictor variable. Our main findings remain robust to this change.

### Meteorological data

Meteorological data were collected from China Meteorological Data Service Center (http://data.cma.cn/), Hong Kong Observatory (https://www.hko.gov.hk/sc/index.html), Macau Meteorological and Geophysics Bureau (https://www.smg.gov.mo/en), and Taiwan Central Weather Bureau (https://codis.cwa.gov.tw/StationData), which report daily mean temperature, wind speed, wind direction, relative humidity, air pressure, total precipitation, and total sunshine hours, for ∼877 weather stations. The datasets also report coordinates of each weather station. Daily weather data were aggregated to generate monthly averages of these weather variables.

### Other predictor variables for O_3_ estimation

We extracted normalized difference vegetation index (NDVI) and elevation data at 1-km resolution from the Institute of Geographic Sciences and Natural Resources Research of the Chinese Academy of Science for years 2002–2019 (https://www.resdc.cn/Default.aspx). Population density at 1-km resolution was downloaded from the WorldPop datasets (https://www.worldpop.org/).

### Merging datasets

We merged the ground O_3_ data, satellite-derived pollution data, and meteorological data from 2013 to 2019 by grid cell and month to train the machine learning models. The surface O_3_ data and the satellite-derived pollution data were merged by overlaying two maps: one with locations of air quality monitoring stations and another with satellite grid cells. Because air quality monitoring stations in China are not evenly distributed, some grid cells may contain more than one monitoring station. For those grid cells, we took an average of monthly mean MDA8 O_3_ concentrations across monitoring stations within a grid cell. To match up with our pollution data, we employed an inverse distance weighting (IDW) method to impute meteorological data for each of the grid cells covering China. Specifically, we chose a radius of 200 km surrounding the centroid of a grid cell and computed the weighted averages of meteorological variables recorded by all weather stations within the circle, with the distance to the centroid of the grid cell as the weight. The NDVI, elevation and population density data at 1-km spatial resolution were aggregated to the grid level at a spatial resolution of 0.5°×0.625° using ArcGIS.

### Ground PM_2.5_ estimates

We obtained daily PM_2.5_ concentrations with a spatial resolution of 10 km × 10 km from a near real-time air pollutant database in China (http://tapdata.org.cn/) (62). The grid-level PM_2.5_ data were processed to impute county-level PM_2.5_ concentrations using the similar IDW method described above.

### Agricultural TFP estimates

We employed four approaches to estimate county-level agricultural TFP. The baseline model is the specification based on the TL-CPF. We considered alternative specifications based on the Translog conventional production function with constant returns to scale (TL-CPF-w/CRS), the Cobb–Douglas conventional production function without constant returns to scale (CD-CPF), and Cobb–Douglas stochastic frontier model with constant returns to scale (CD-SFA-w/CRS). In all models, the output variable is the aggregate agricultural outputs, which are the sum of the deflated total value of outputs from cropping, livestock, forestry, and fisheries. There are four primary inputs, including cropland, agricultural labor, fertilizer, and machinery. We excluded the Tibetan conservation zone and northwestern counties from our analysis. The former covers most of the Qinghai-Tibet plateau with highly fragmented agricultural production, while the latter was excluded because of poor performance of machine learning models in northwest. Because county-level agricultural data were only available up to 2015, we estimated agricultural TFP for 2,298 counties over the 2002–2015 period.

### Yield data

The National Bureau of Statistics (NBS) provided county-level administrative data on agricultural outputs in mainland China from 2002 to 2015. The dataset contains county-specific total crop production (measured in metric tons) and planted acreage (measured in hectares) for major food/feed crops. These major crops include rice, wheat, maize, soybeans, and tuber crops. Several rice cropping systems are practiced in China, including single-season rice, double-cropped rice (a combination of early and late rice production technology), and multiple-cropped rice. The dataset does not report total production and planted acreages for early and late rice in regions with double or multiple rice cropping systems. To accurately match yield data with pollution and weather data, we focused solely on single-season rice production. We calculated county-average crop yields as the total county-level production divided by their respective planted acreage. Regarding livestock, the NBS dataset reports county-level milk production (measured in metric tons) and the total number of cows (measured in heads). We computed milk production per cow.

### Machine learning model training

We employed three machine learning algorithms, namely LightGBM, XGBoost, and Super Learner, to estimate ground-level monthly mean MDA8 O_3_ concentrations between 2002 and 2019. Originally developed from the gradient boosting framework based on decision tree learning algorithms, LightGBM and XGBoost are considered as powerful machine learning algorithms (63, 64). These two algorithms significantly improve prediction accuracy, have higher computational efficiency, and reduce the possibility of over-fitting compared to other machine learning algorithms such as random forest (52). Both algorithms are also more interpretable than deep learning models such as neural networks (49, 65). Super Learner is an integrated machine learning algorithm, which combines various ensemble learning models, such as LightGBM, XGboost, random forest, to achieve improved prediction accuracy (66). It creates an optimal weighted average of these candidate algorithms and has been proven to perform asymptotically as accurate as the best possible prediction algorithm in its library (67).

### Predictor variables

Data from 2013 to 2019 were used for machine learning model training. We included a comprehensive set of model predictors to ensure best predictive power of these machine learning models. Several previous studies have shown that meteorological factors and anthropogenic emissions can influence O_3_ concentrations (26). Vegetation plays a role in the formation of ground-level O_3_ by (i) emitting VOCs that serve as O_3_ precursors (68), (ii) removing nitrogen oxides from the air (69), (iii) facilitating dry deposition (68), and (iv) affecting weather conditions like temperature and sunlight. We included population density to account for anthropogenic influences on O_3_ levels, which typically include emissions from traffic and industrial activities. The inclusion of this variable can also account for variations in O_3_ levels between rural and urban areas in China (59). Local characteristics, such as elevation and terrain, can affect ground-level O_3_ by influencing the interplay of chemical, physical and meteorological factors. Therefore, in addition to satellite-based O_3_ concentrations, model predictors included satellite-derived aerosols components (i.e. organic carbon, black carbon, dust, sulfate, and sea salt), meteorological variables (average temperature, relative humidity, air pressure, precipitation, wind speed, and sunshine durations), coordinates, elevation, NDVI, and population density. We performed a grid search for hyperparameters to identify the best model configurations, guided by statistical measures of CV *R*^2^, RMSE, and MAPE values.

Given the likely varying correlations between satellite-based and ground-recorded O_3_ across space, we partitioned all the grid cells in China into six subregions, using a *k*-means clustering algorithm, which minimizes within-cluster variances and aims to identify clusters with similar spatial features (latitudes and longitudes in this study). *K*-means is a well-established algorithm, noted for its simplicity and efficiency in solving clustering problems (70). The six subregions that we created are the North, Northeast, East, PRD, Qinghai-Tibet, and Northwest (Fig. 1). We then trained the three machine learning models separately for each of these subregions.

### Model validation

We applied 10-fold cross-validation (CV) to assess model performance. Ten-fold CV is commonly employed in machine learning studies, as it can generate test error rate estimates free from both high bias and large variance (71). In this process, the merged dataset with monthly records from 2013 to 2019 was randomly partitioned into ten equal size subsets. Nine of these subsets were used to train a machine learning model, while the remaining one was reserved as the validation data for testing the model. This cross-validation process was repeated 10 times (the folds) to generate CV O_3_ concentrations corresponding to each monthly mean observation that was used for model training. Using the CV-generated O_3_ estimates and the corresponding observations, simple linear regressions were performed to calculate *R*^2^, RMSE, and MAPE for evaluating model performance.

### Econometric model

We estimated the following model to assess the impacts of pollution and temperature on agricultural TFP:


(1) $$\log\;({\mathrm{TFP}}_{it})=\beta_{\mathrm{Ozone}}{\mathrm{Ozone}}_{it}+\beta_{\mathrm{PM}}\mathrm{PM}2.5_{it}+X_{it}\gamma +\alpha_{i}+\lambda_{t}+u_{it}$$

where $\mathrm{TF}P_{it}$ represents the agricultural TFP in county *i* in year *t*. ${\mathrm{Ozone}}_{it}$ and $\mathrm{PM}2.5_{it}$ denote annual average MDA8 O_3_ and PM_2.5_ concentrations, respectively. Given the sensitivity of agricultural productivity to weather and the correlations between air pollutant concentrations and meteorological factors, we controlled for a flexible set of weather variables, denoted by the vector **X***_it_*, which includes total precipitation, total sunshine duration, average relative humidity, air pressure, and wind speed, all at the annual level. We considered linear and quadratic terms of these variables to allow for potential nonlinear effects. **X***_it_* also contains a set of temperature variables that measure the number of days with daily temperature falling into a specific bin. We conducted a sinusoidal interpolation between daily maximum and minimum temperatures before forming the temperature bins, which allows for a portion of a day to be counted toward a certain temperature bin. We set up 5°C bins, with the first bin being temperatures below 0°C and the last bin accounting for temperatures above 35°C. *α_i_* represents county fixed effects, controlling for time-invariant location-specific unobserved factors, such as geography. *λ_t_* denotes year fixed effects that control flexibly for common time-varying shocks that were experienced by all counties in our sample, such as technological changes. *u_it_* represents the error term. Our coefficients of interest are $\beta_{\mathrm{Ozone}}$ and $\beta_{\mathrm{PM}}$, which are interpreted as the percentage change in TFP induced by each unit increase in O_3_ or PM_2.5_. We clustered standard errors at the county level, but our results are robust to alternative clustering choices (Table S7).

The OLS estimators of $\beta_{\mathrm{Ozone}}$ and $\beta_{\mathrm{PM}}$ are prone to bias. Following prior studies (41, 42, 72), we overcame these econometric challenges by using an IV approach that relies on changes in wind direction as exogenous shocks to local pollution levels. Because wind can transport ambient pollutants hundreds of kilometers away, wind direction is a strong predictor of local pollution levels. More importantly, wind direction is unlikely to directly affect agricultural productivity except through its impacts on air pollution. Specifically, we estimated the following first stage model:


(2) $$\begin{array}{rl} \{{\mathrm{Ozone}}_{it}\;\mathrm{or}\;\mathrm{PM}2.5_{it}\}\;= & \underset{g\in G}{\sum }\;\overset{2}{\underset{a=0}{\sum }}\;\pi_{a}^{g}1[G_{i}=g]\times WD_{it}^{90a,90a+90}+X_{it}\gamma \\ & +\alpha_{i}+\lambda_{t}+\sigma_{i,t}. \end{array}$$

The variable $1[G_{i}=g]$ is an indicator for county *i* being assigned to group *g* from the set of county group *G*. We used the *k*-means cluster algorithm to generate 50 groups for all the sample counties based on their coordinates. The variable $WD_{it}^{90a,90a+90}$ measures the number of days in county *i* in year *t* with the daily average wind direction falling in a specific 90° interval. We chose the range of values from 270° to 360° as the reference category. The interaction term $1[G_{i}=g]\times WD_{i,t}^{90a,90a+90}$ thus contains our excluded instruments. Our results remained robust to variations in the numbers of spatial groups and wind direction bins (Table S9). The coefficient $\pi_{a}^{g}$ captures the influence of wind direction on pollution, and it is allowed to vary across regions. Other control variables and the fixed effects were constructed the same as in Eq. (1).

### Cumulative indices of O_3_

These three cumulative indices were calculated as: $\mathrm{AOT}40=\overset{n}{\underset{h=1}{\sum }}\;(C_{h}-40)$ for *C_h_* > 40 ppb, $\;\;\mathrm{SUM}06=\overset{n}{\underset{h=1}{\sum }}\;C_{h}$ for *C_h_* > 60 ppb and $W126=\overset{n}{\underset{n=1}{\sum }}\;\left(C_{h}\times \frac{1}{\left(1+4403\times e^{-126\times C_{h}}\right)}\right)$, where $C_{h}\;$ is the hourly O_3_ concentration in ppb for hour h, and n is the number of hours. These vegetation indices were calculated for the entire year. Since O_3_ pollution primarily occurs during the nonwinter seasons, coinciding with the growth periods for most crops, the magnitudes of these year-round indices are nearly identical to those computed solely for the nonwinter season (Table S3). We made two simplifying assumptions to compute hourly O_3_ concentrations over the 2002–2015 period. First, we assumed that the hourly O_3_ concentrations during the peak 8 h (or during the nonpeak hours) each day in a month were the same, and that the hourly O_3_ concentrations during the peak hours are equal to the monthly mean MDA8 O_3_ concentrations predicted by machine learning models. Second, we assumed that the ratio of mean O_3_ concentrations during the peak 8 h to that during the nonpeak hours, though differing by month and by region, remained stable. We computed these ratios for each month and each region using the observed hourly data over the 2013–2019 period, and then estimated the mean hourly O_3_ concentrations during the nonpeak hours for years 2002–2015.

### Uncertainty analyses

We conducted a range of analyses to address potential uncertainties. These analyses included the use of validated ground-level PM_2.5_ data for training machine learning models, exclusion of NDVI and population density as predictor variables, and exclusion of weather stations located within either 10 or 20 km of city centers in the IDW interpolation. These analyses were conducted using the Super Learner model. The results show that the model performance (*R*^2^, RMSE, and MAPE) and the predicted spatial and temporal distributions of O_3_ concentrations remain robust across these variations (Fig. S12). Using the O_3_ estimates generated from these scenarios, we then reconstructed the average MDA8 O_3_ concentrations for both winter and nonwinter seasons and estimated Eqs. (1) and (2) to assess the impacts of pollution and temperature extremes on agricultural TFP. The results were consistent with our baseline findings (Fig. S13).

## Supplementary Material

pgad435_Supplementary_Data

## Data Availability

The data and code that support the findings of this study are openly available in a permanent repository on Zenodo (https://doi.org/10.5281/zenodo.10280292).
