# Supplementary material for: The spatiotemporal pattern of surface ozone and its impact on agricultural productivity in China
Source: PNAS Nexus. 2023 Dec 14;3(1):pgad435. doi: 10.1093/pnasnexus/pgad435 (PMC10752353; doi:10.1093/pnasnexus/pgad435)
Supplement: pgad435_Supplementary_Data [file pgad435_supplementary_data.docx]

Supplementary Materials for

**The Spatiotemporal Pattern of Surface Ozone and Its Impact On Agricultural Productivity in China**

Xiaoguang Chen et al.

*Corresponding author. Email: cxg@swufe.edu.cn

**This PDF file includes:**

Supplementary Text

Figs. S1 to S13

Tables S1 to S24

References (1 to 3)

**Supplementary Text**

**Robustness Checks**

We conducted a number of robustness checks to examine the stability of the baseline estimates. First, we considered different clustering choices, including a two-way clustering by county and by year (or by region-year), which accounts for spatial and temporal correlations of the error terms (Table S7). Second, we examined the robustness of our findings to alternative specifications (including different types of fixed effects and time trends) and weather controls (Table S8). Third, to investigate the validity of the monotonicity assumption of the instrumental variables, we estimated alternative specifications by allowing instruments to vary with the size of wind angle bins and the number of county groups (Table S9). We obtained estimates that were similar to our baseline estimates, indicating that our estimates unlikely suffered from the violations of the monotonicity assumption. Fourth, our main specifications included over 150 instruments, which might raise concern about weak instrument bias. To confirm the reliability of our instrument variables, we then estimated our main specification using the limited information maximum likelihood (LIML) estimator (last column of Table S9), which is approximately median unbiased even with many weak instruments. Fifth, to ensure that the baseline estimates were not driven by potential outliers, we replaced the TFP estimates that are larger (or smaller) than the 99.5th (or the 0.5th) percentile by the 99.5th (or the 0.5th) percentile estimates. We also removed the observations if the TFP estimates are larger (or smaller) than the 99.5th (or the 0.5th) percentile (Table S10). Sixth, we excluded regions that heavily depend on fisheries from the sample due to the limited evidence that fisheries are affected by pollution (Table S11). Seventh, we excluded the PM_2.5_ variable from the regression model to examine whether the estimated impacts of O_3_ on agricultural productivity are sensitive to the removal of this pollution covariate (Table S12). Lastly, we conducted two sets of placebo checks to ensure that the estimated relationship between pollution and TFP did not arise by chance. We first estimated the relationship between pollution and agricultural TFP using 1,000 datasets that were generated by randomly mismatching the county-year TFP and pollution data. We subsequently estimated the models with another 1,000 datasets generated by randomizing TFP and pollution data within seasons and regions. Fig. S8 plots the resulting distributions of the estimates based on these placebo datasets. We found that the sample estimate falls outside these distributions. All these results provide strong support to the conclusion that elevated O_3_ pollution during the non-winter seasons reduce agricultural TFP.

|  | Annual Spring Summer Fall Winter |
| --- | --- |
| Overall | 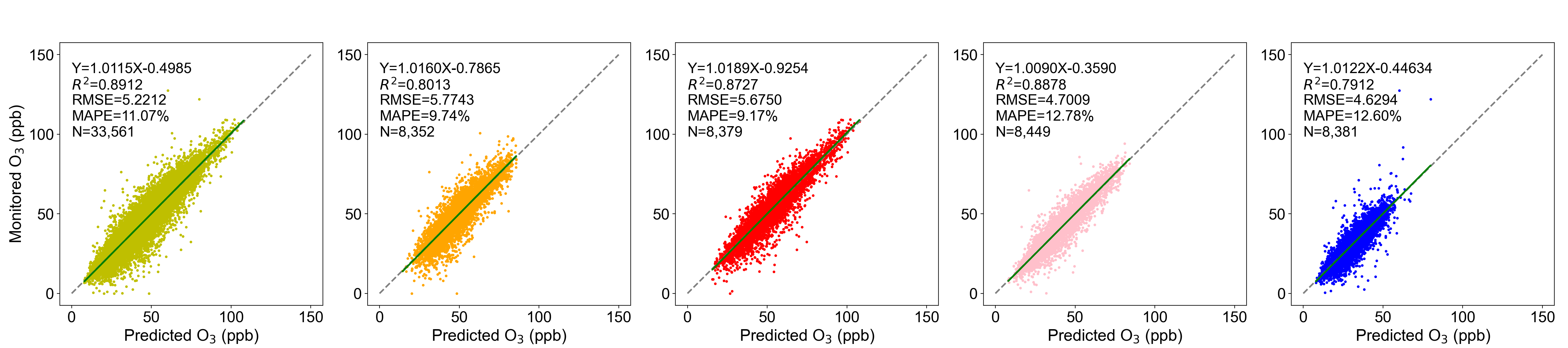 |
| North | 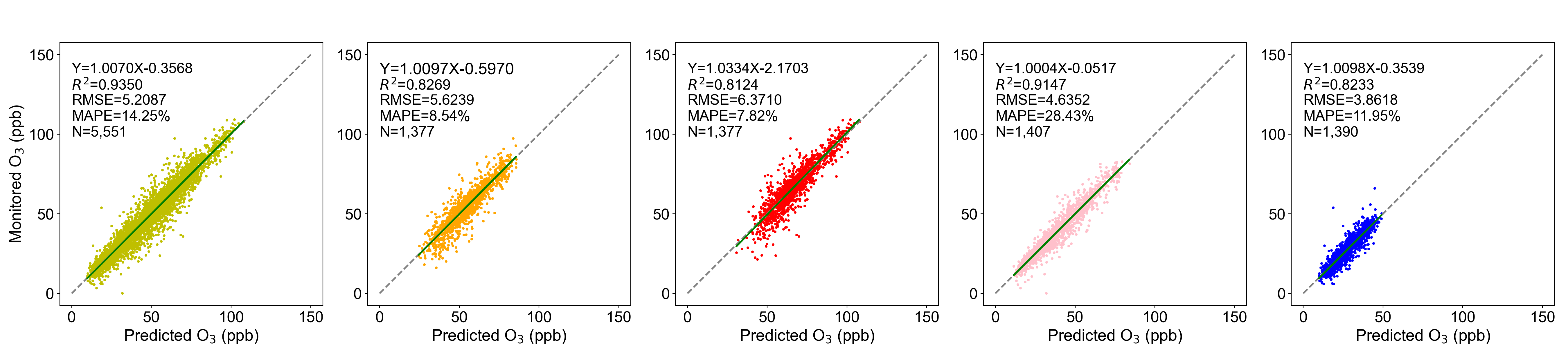 |
| Northeast | 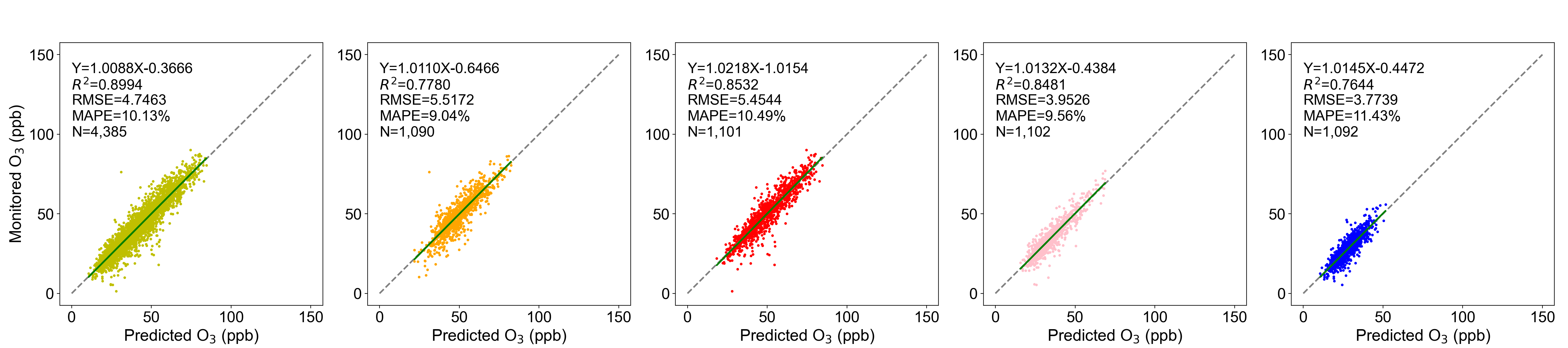 |
| East | 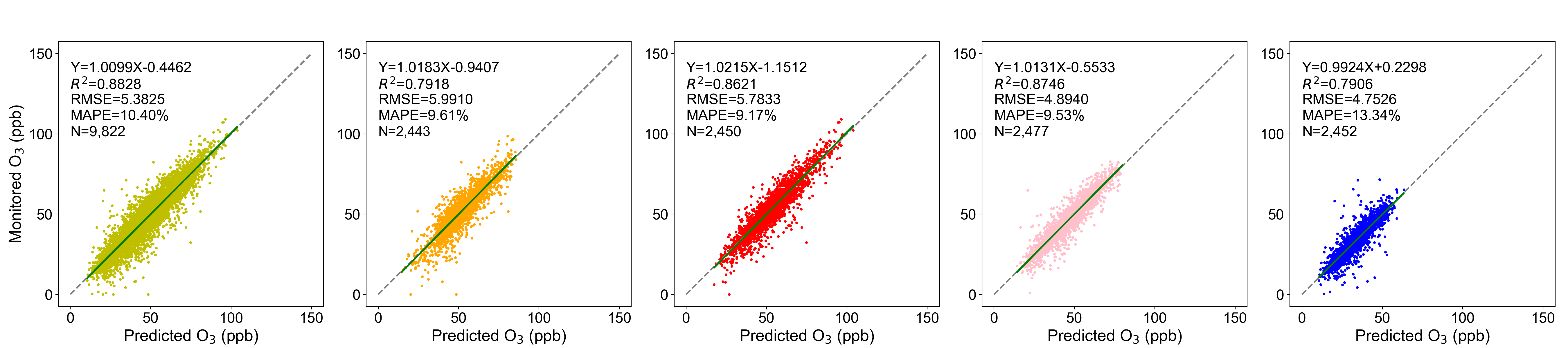 |
| PRD | 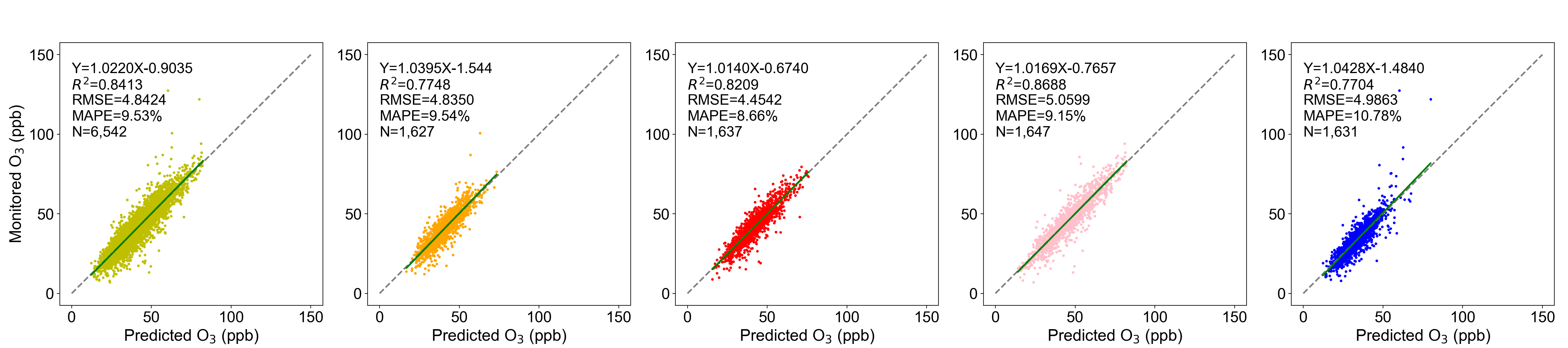 |
| Qinghai-Tibet | 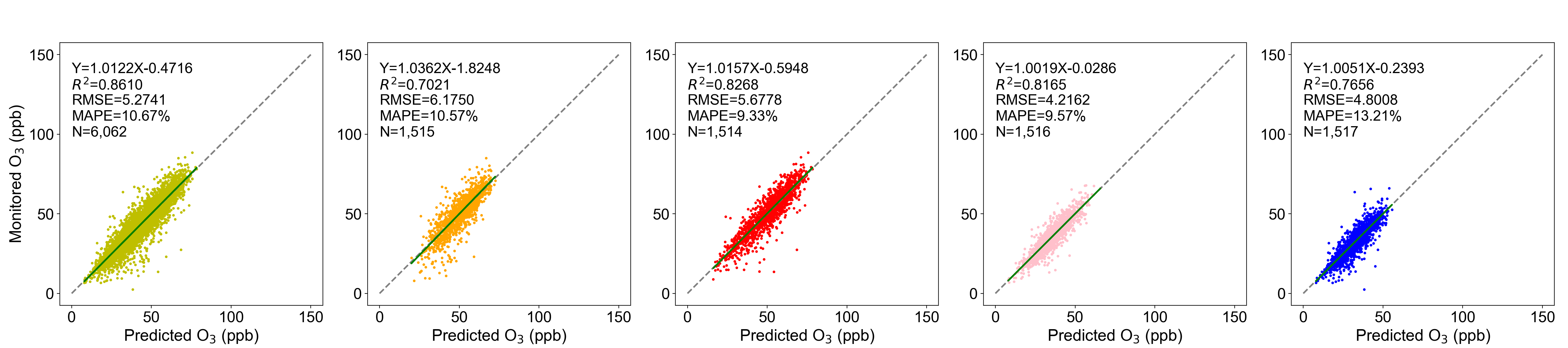 |
| Northwest | 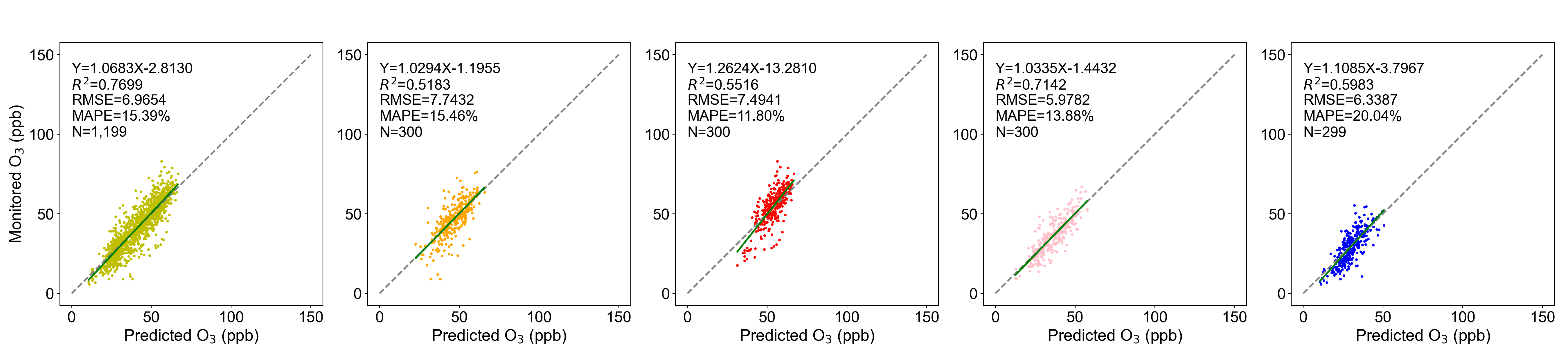 |
|  | **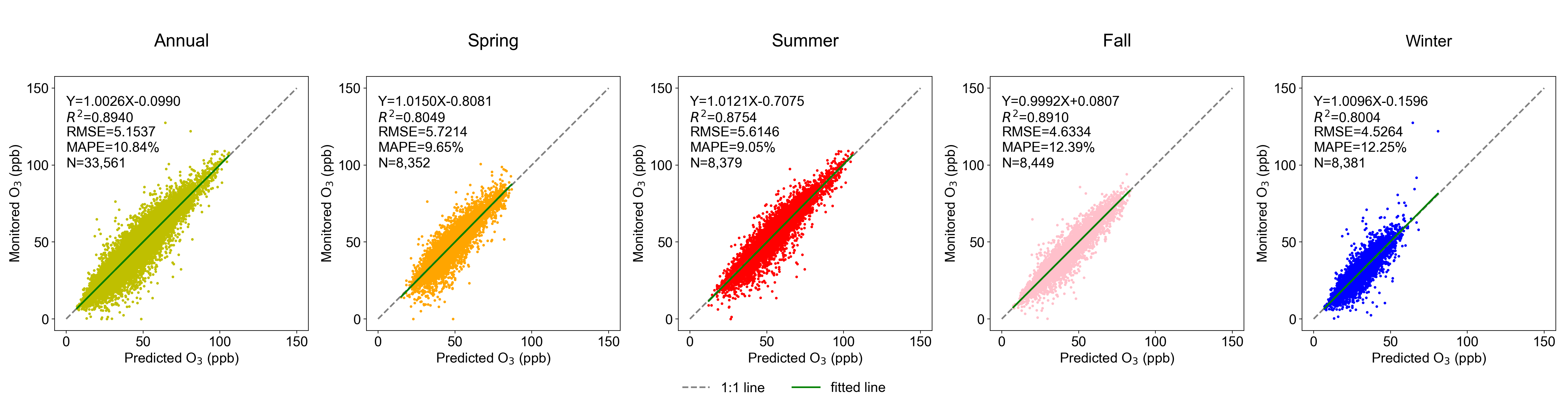** |

**Fig. S1.** Cross validation performance of the LightGBM model across seasons in six subregions of China at the monthly level. These figures show density scatter plots of the monthly predicted MDA8 O_3_ levels vs monitored levels from 2013 to 2019. RMSE stands for root-mean-squared prediction error. MAPE stands for mean absolute percentage error. PRD denotes the Pearl River Delta region.

|  | Annual Spring Summer Fall Winter |
| --- | --- |
| Overall | 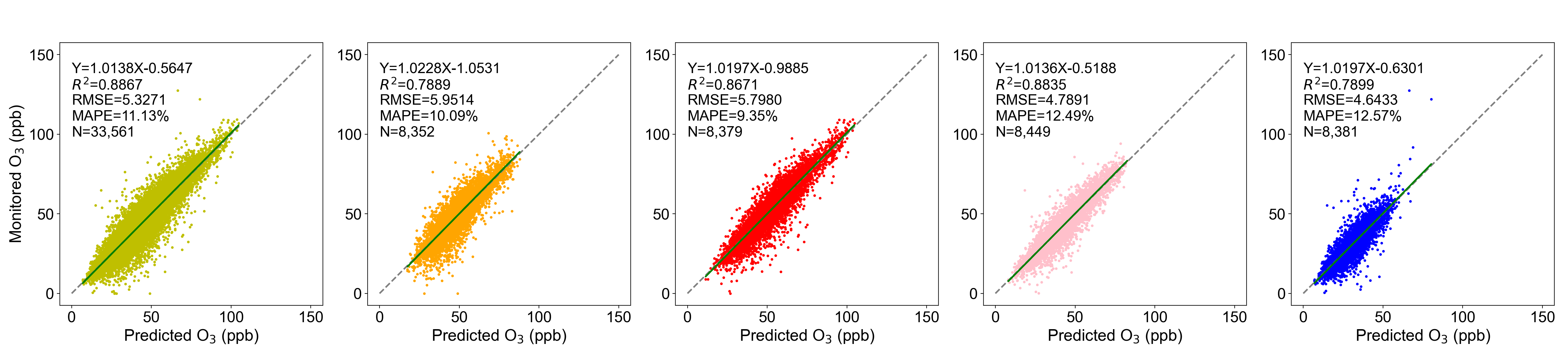 |
| North | 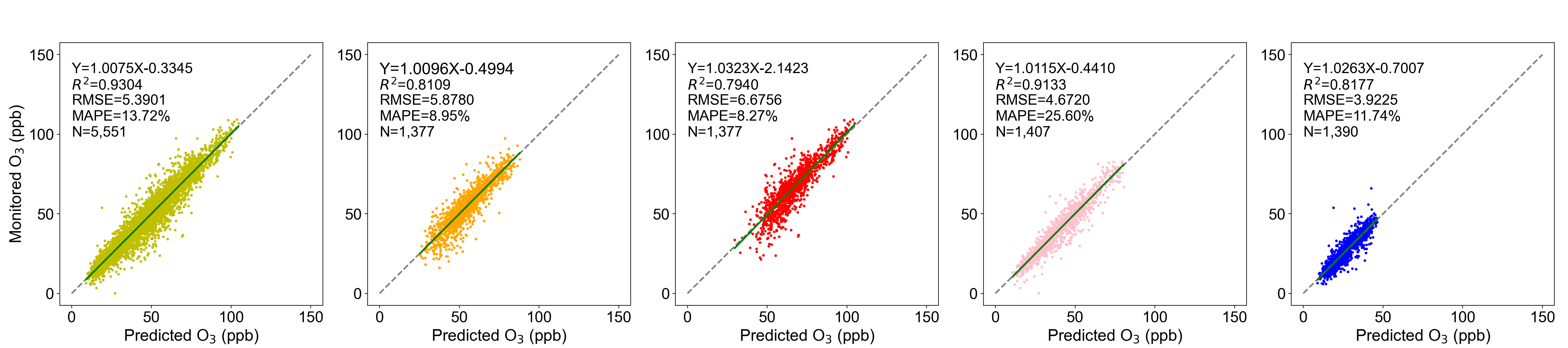 |
| Northeast | 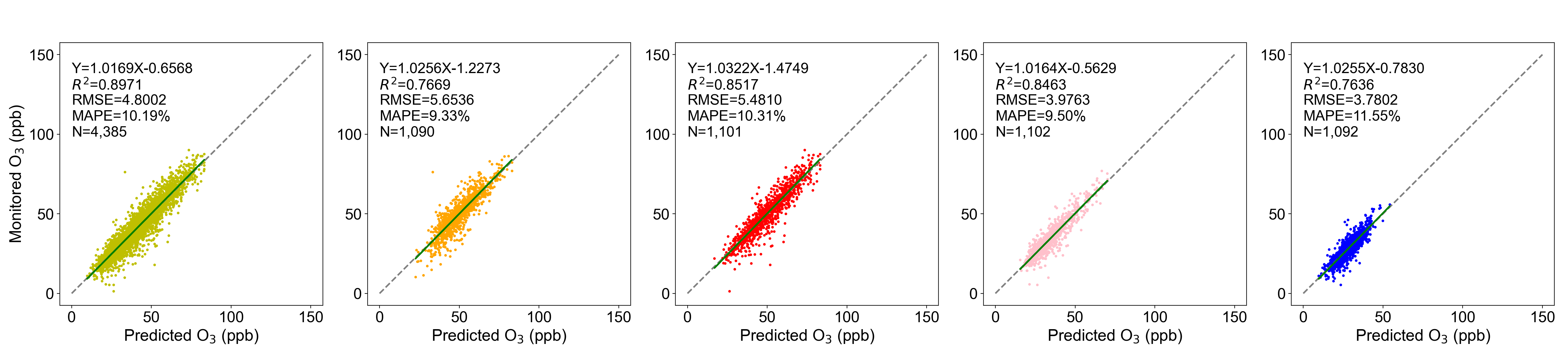 |
| East | 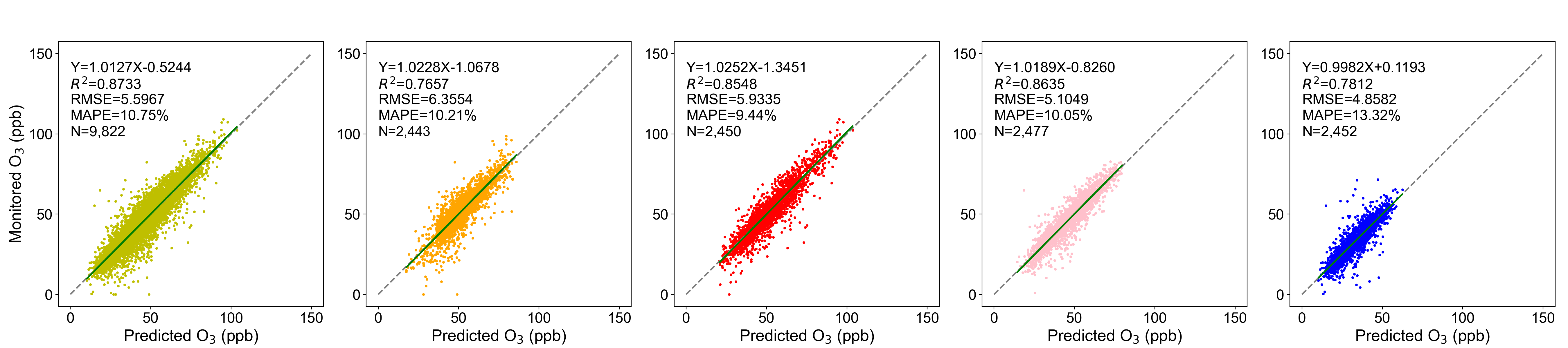 |
| PRD | 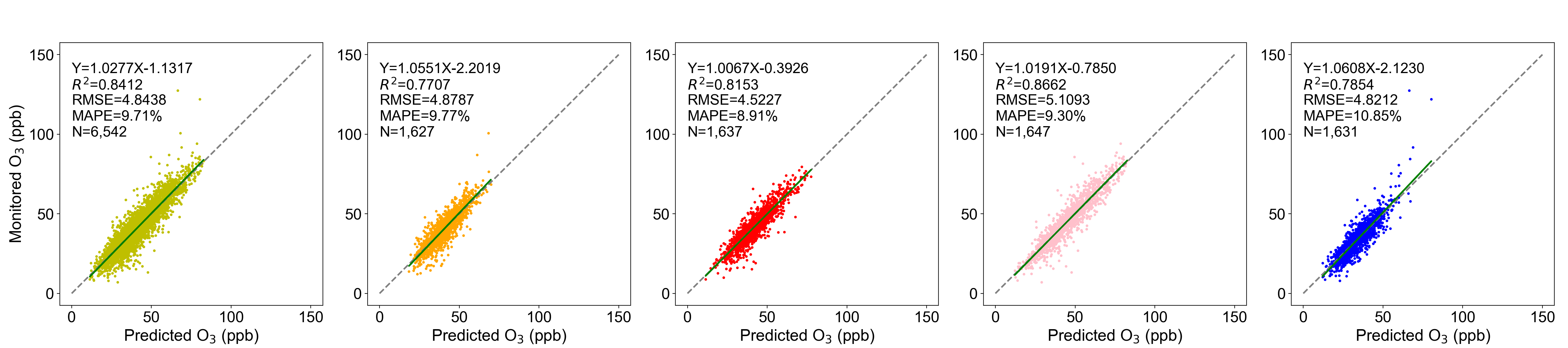 |
| Qinghai-Tibet | 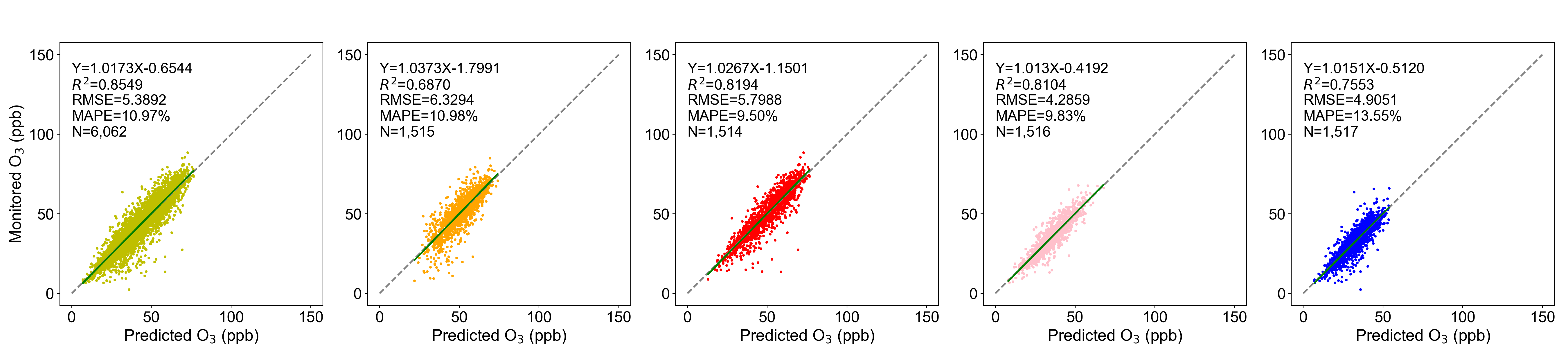 |
| Northwest | 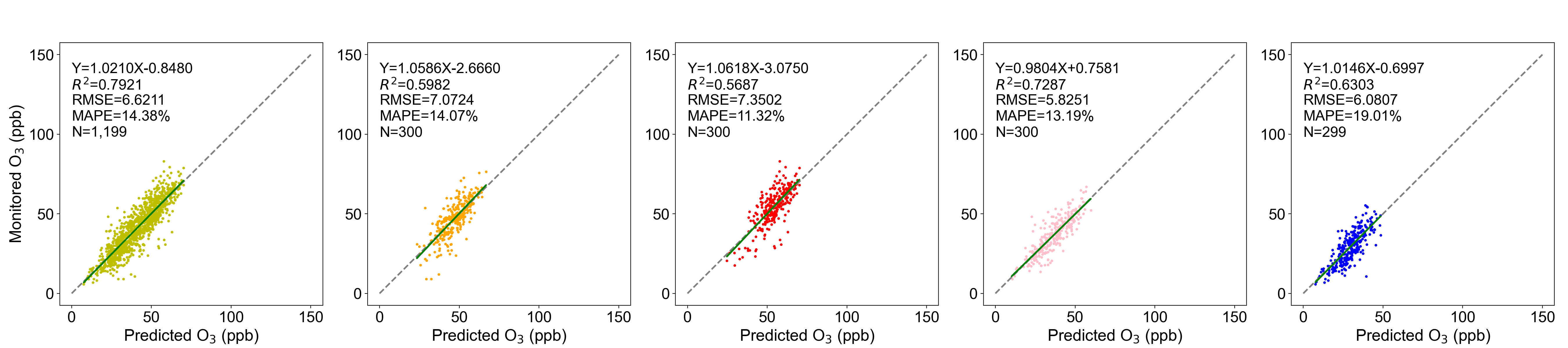 |
|  | **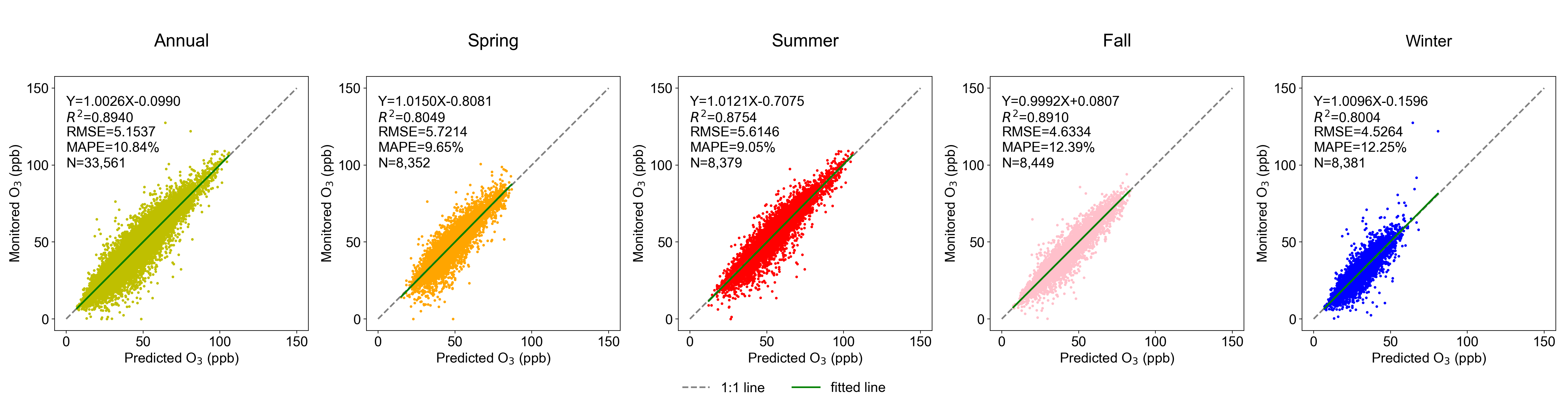** |

**Fig. S2.** Cross validation performance of the XGBoost model across seasons in six subregions of China at the monthly level. These figures show density scatter plots of the monthly predicted MDA8 O_3_ levels vs monitored levels from 2013 to 2019. RMSE stands for root-mean-squared prediction error. MAPE stands for mean absolute percentage error. PRD denotes the Pearl River Delta region.

| 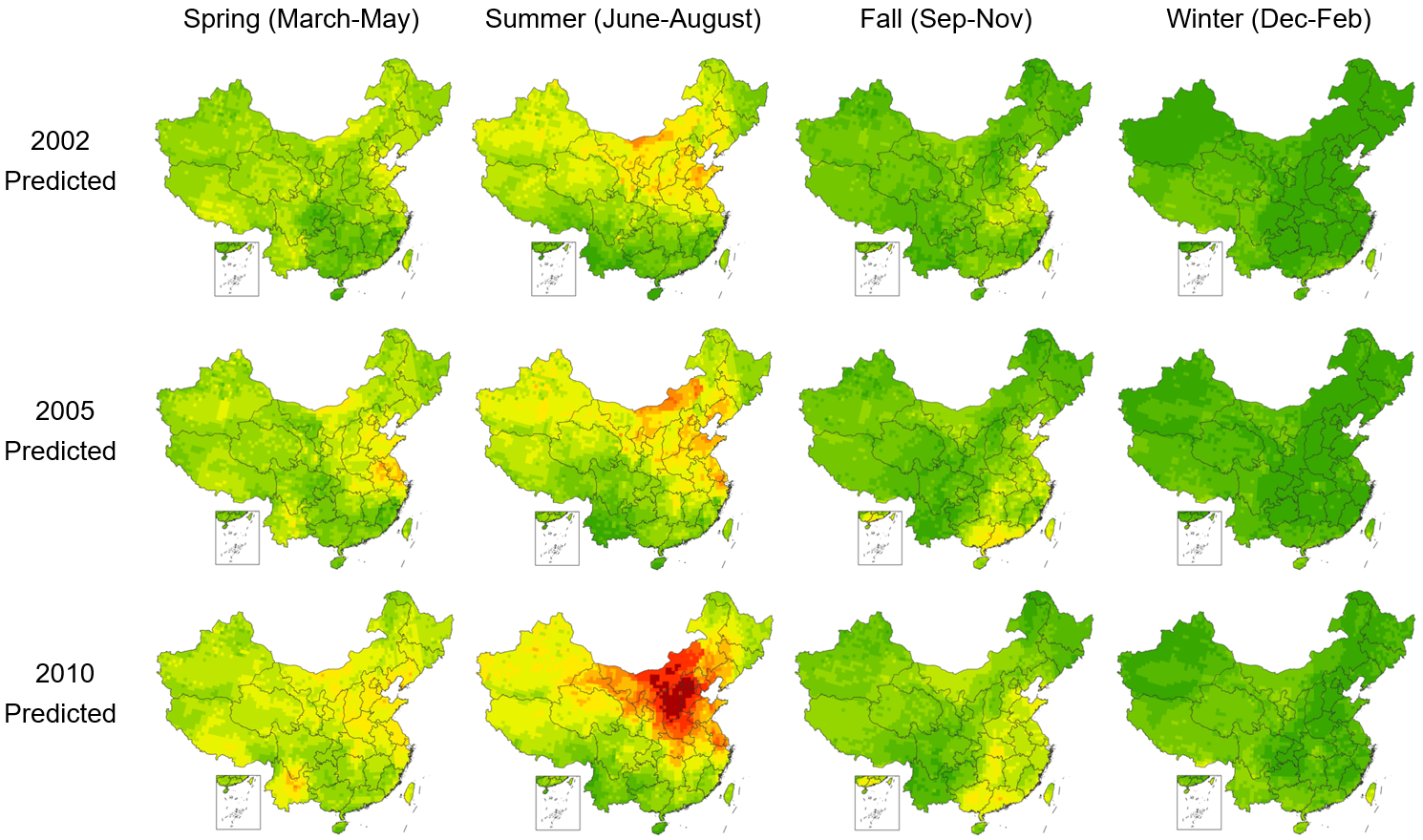 |
| --- |
| 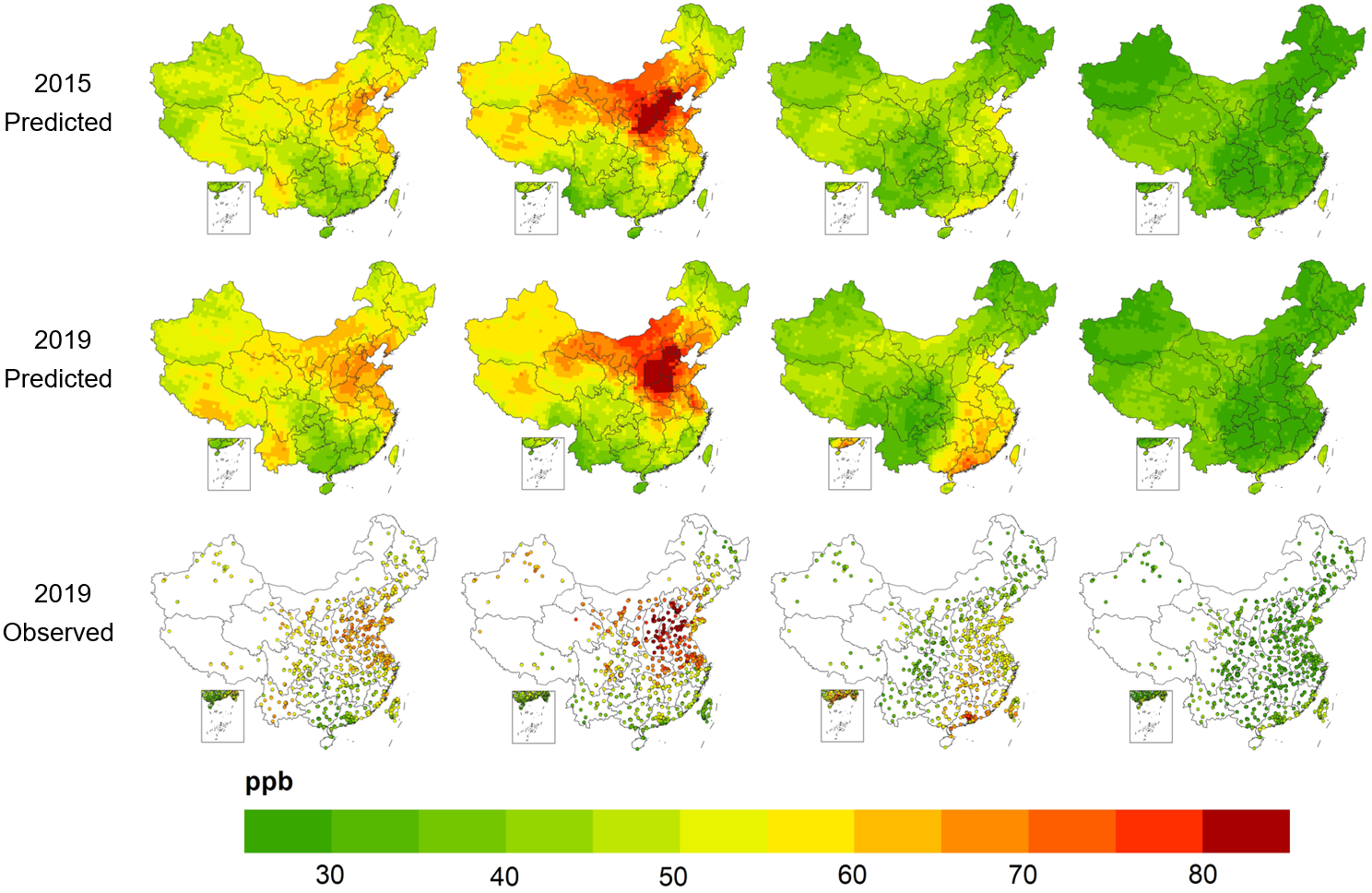 |

**Fig. S3.** Spatial distribution of monthly mean MDA8 O_3_ concentrations in China from 2002 to 2019 at 45 km × 55 km spatial resolution. Maps in top five rows show O_3_ estimates based on the LightGBM model. Maps in the bottom row show observed O_3_ levels in 2019.

| 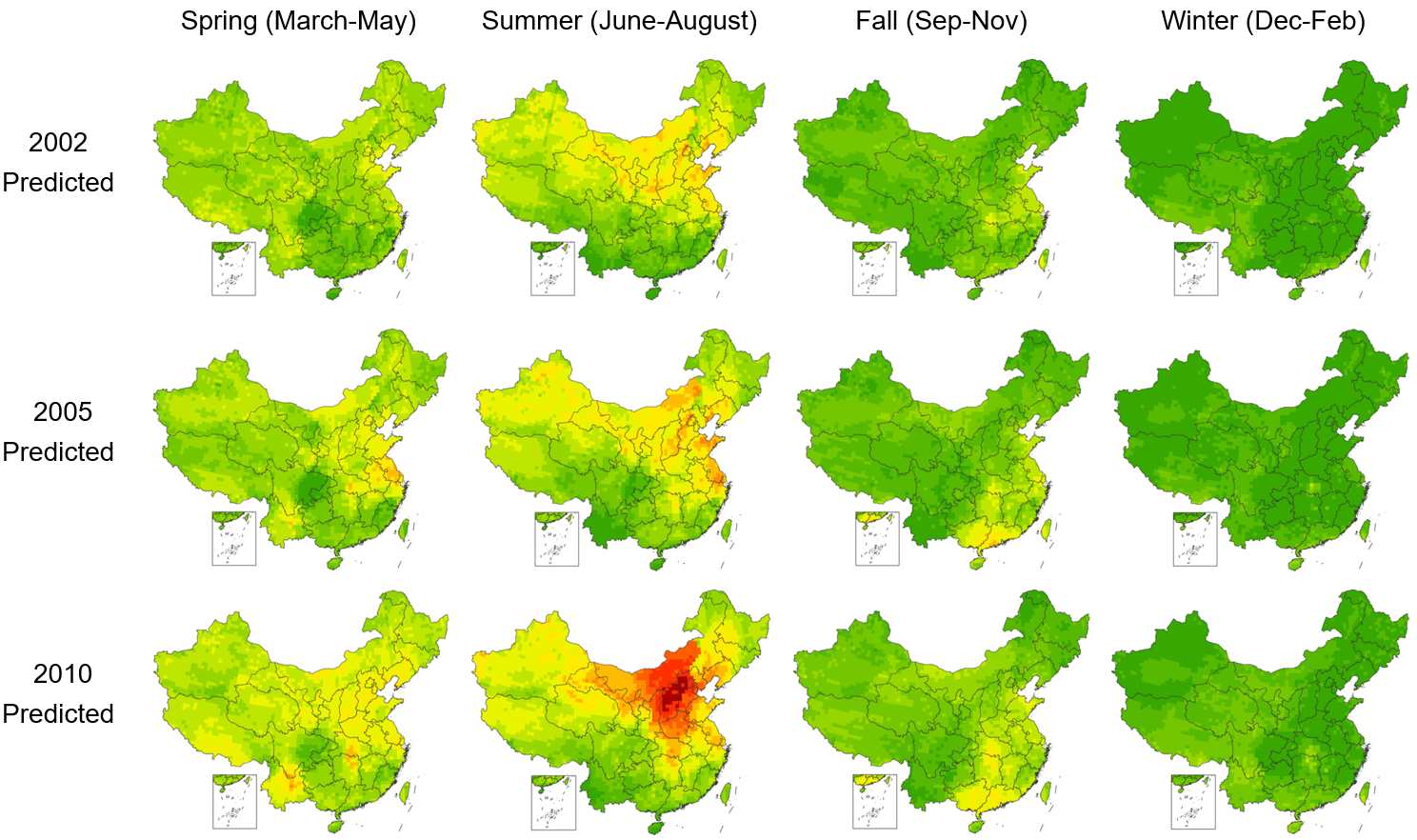 |
| --- |
| 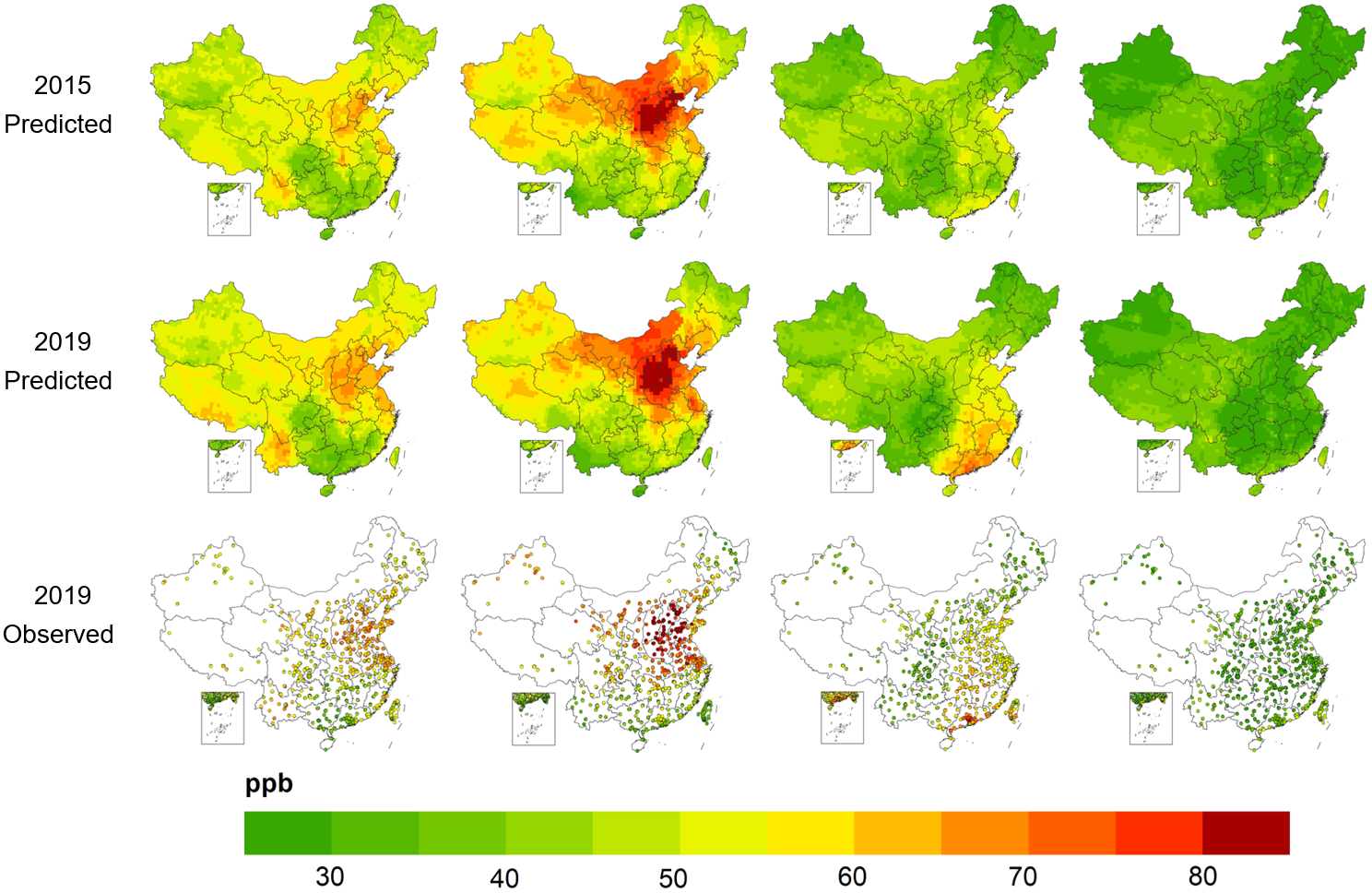 |

**Fig. S4.** Spatial distribution of monthly mean MDA8 O_3_ concentrations in China from 2002 to 2019 at 45 km × 55 km spatial resolution. Maps in top five rows show O_3_ estimates based on the XGBoost model. Maps in the bottom row show observed O_3_ levels in 2019.


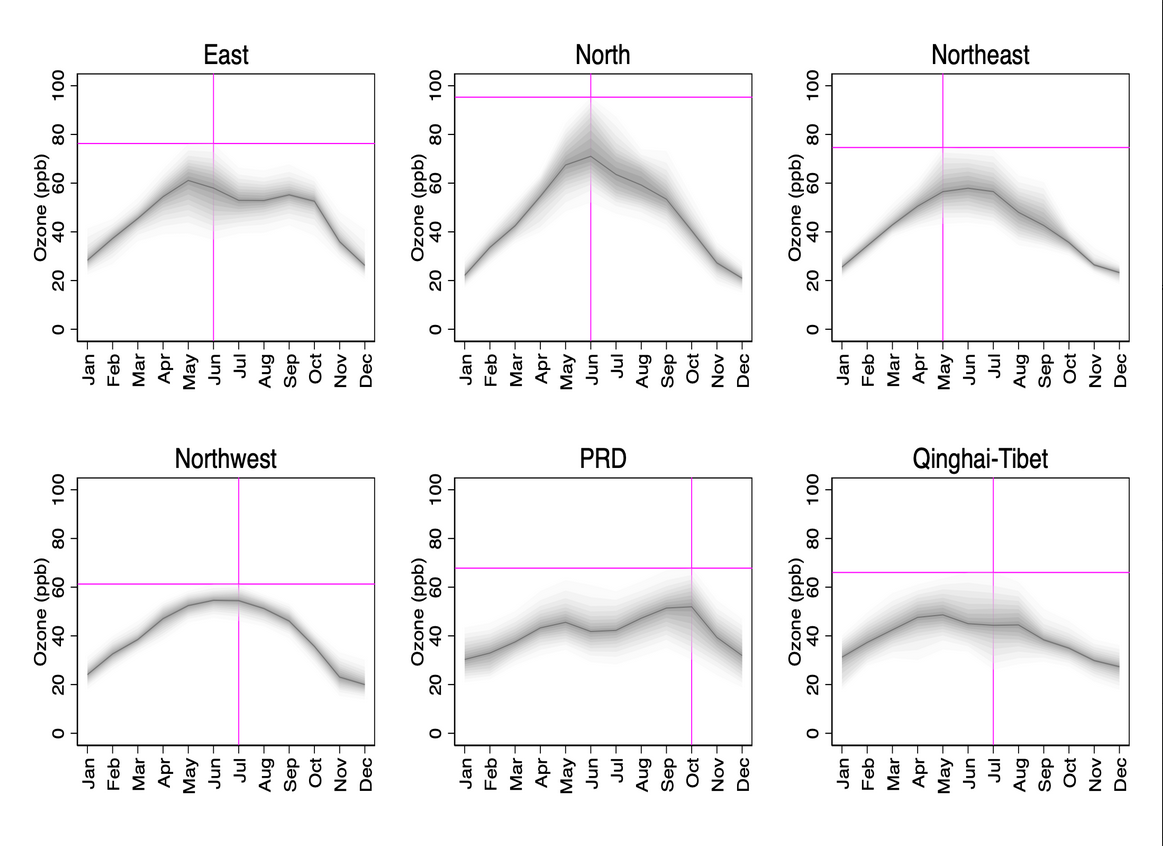


**Fig. S5.** Distribution of predicted monthly mean MDA8 O_3_ concentrations in six subregions of China from 2002 to 2019. These predictions were generated using the Super Learner model. The fan-shaped diagram illustrates the range from the 95th to the 5th percentile of O_3_ concentrations. Each shade increases the percentile by 5%. A magenta cross shows the month with the highest average 95th percentile concentration. The moving center of the cross shows that this peak concentration month varies across the regions.

| A | B |
| --- | --- |
| 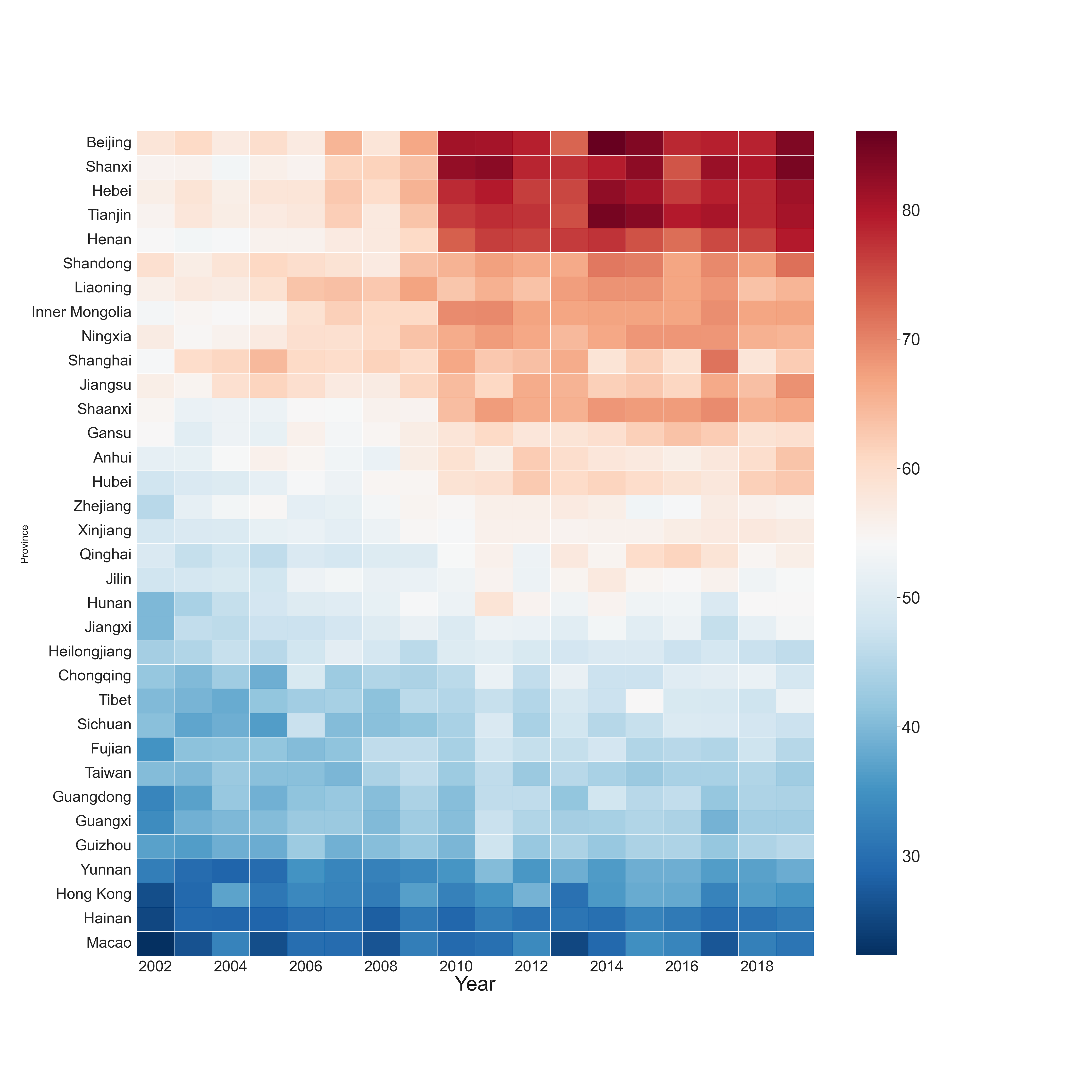 | 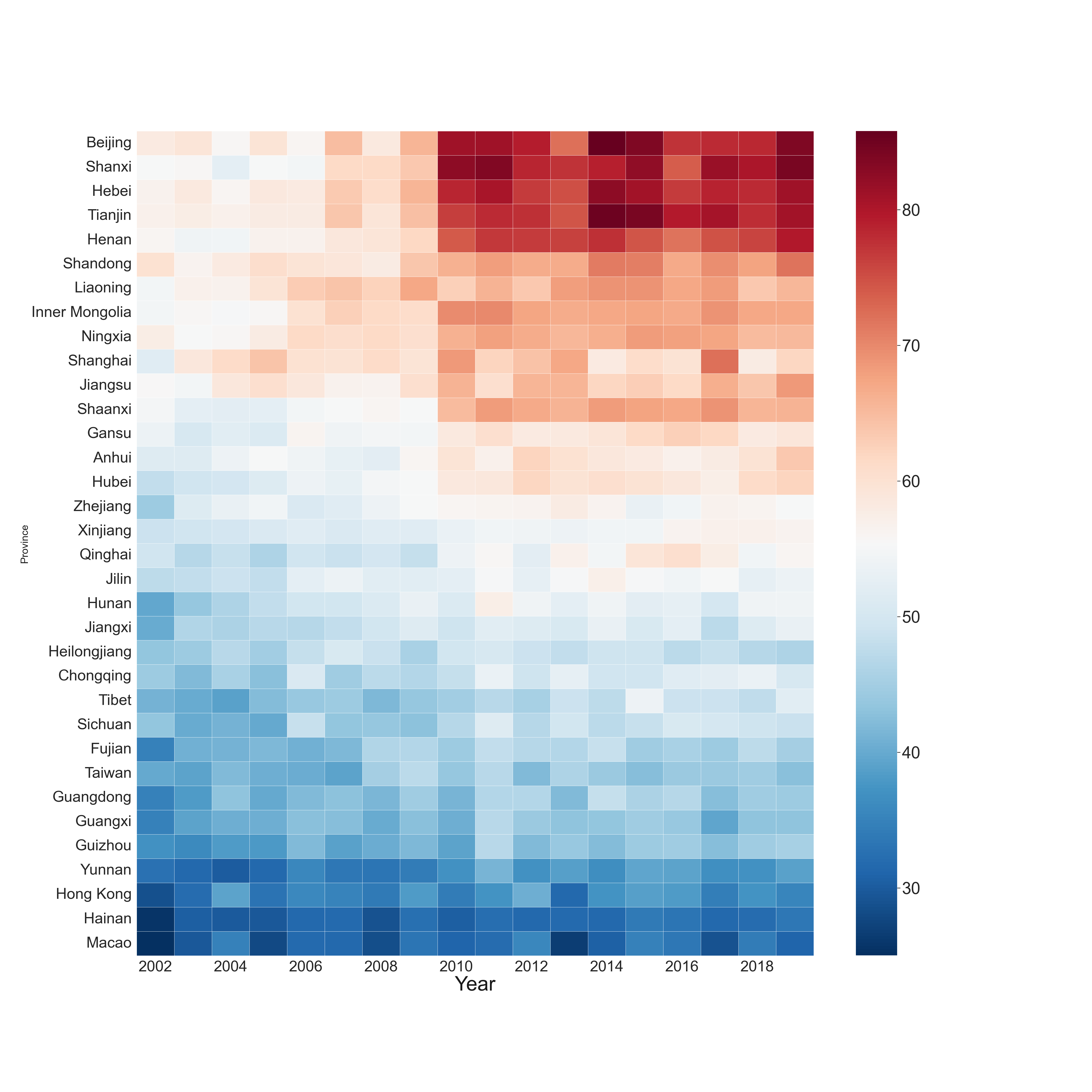 |
| C | D |
| 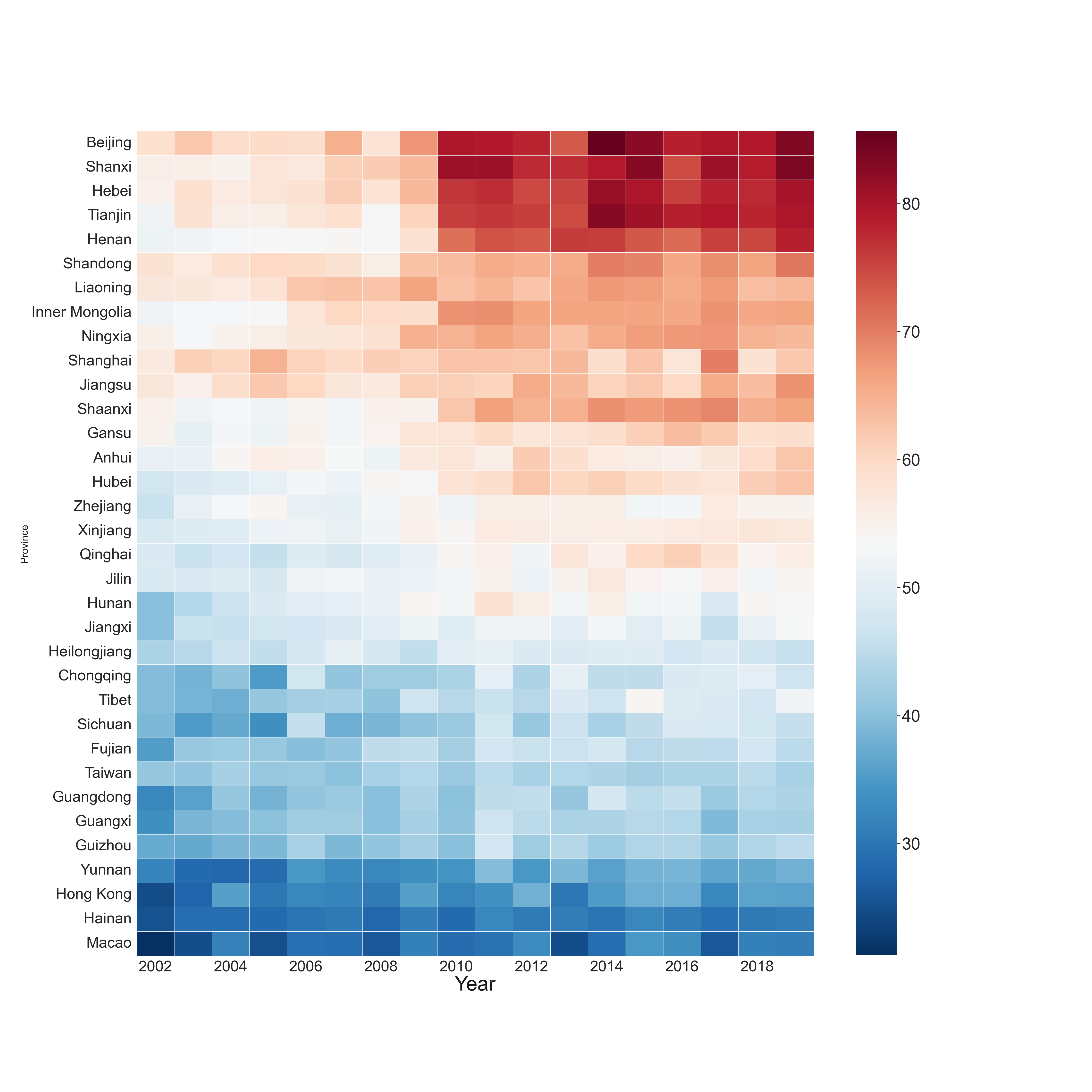 | 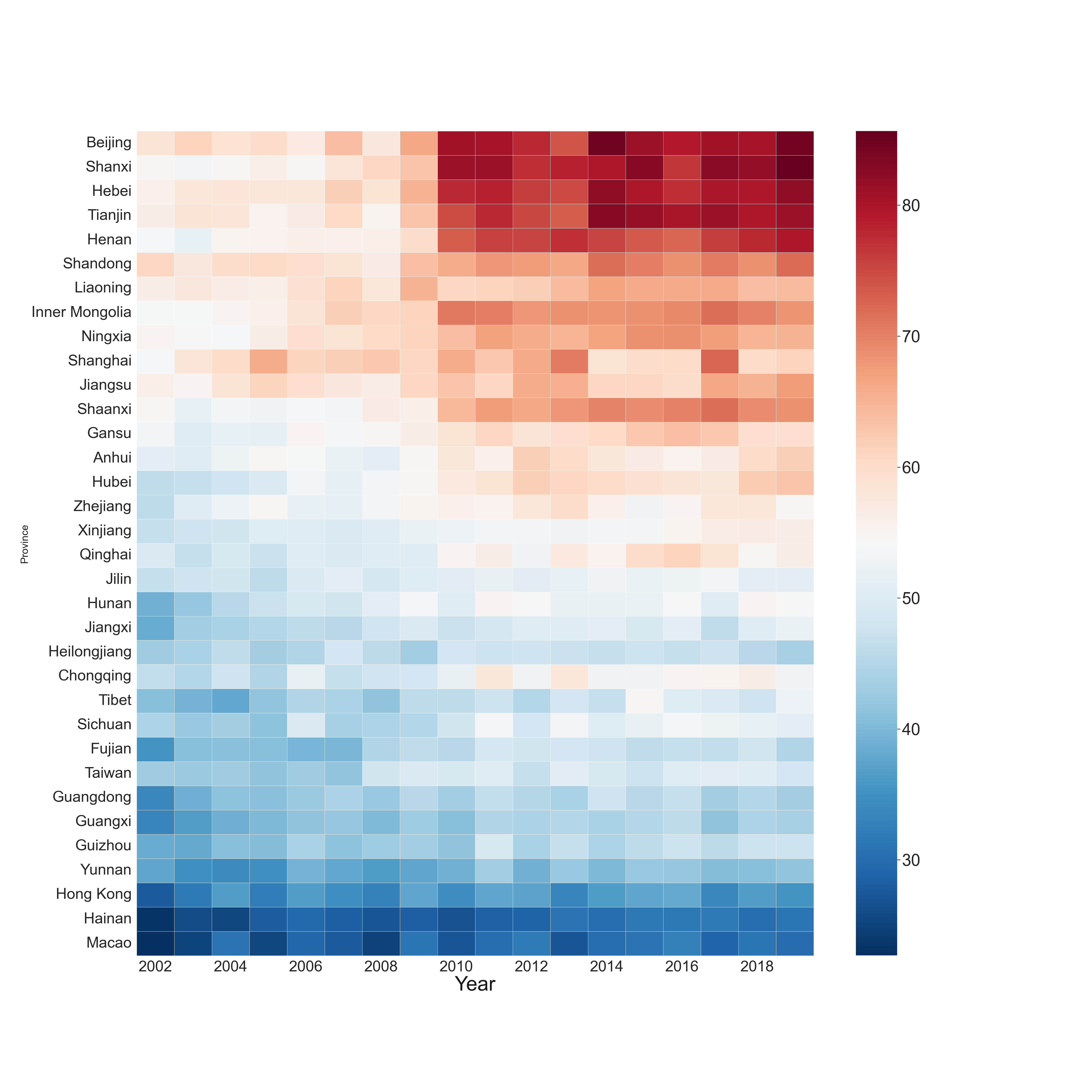 |

| E | F |
| --- | --- |
| 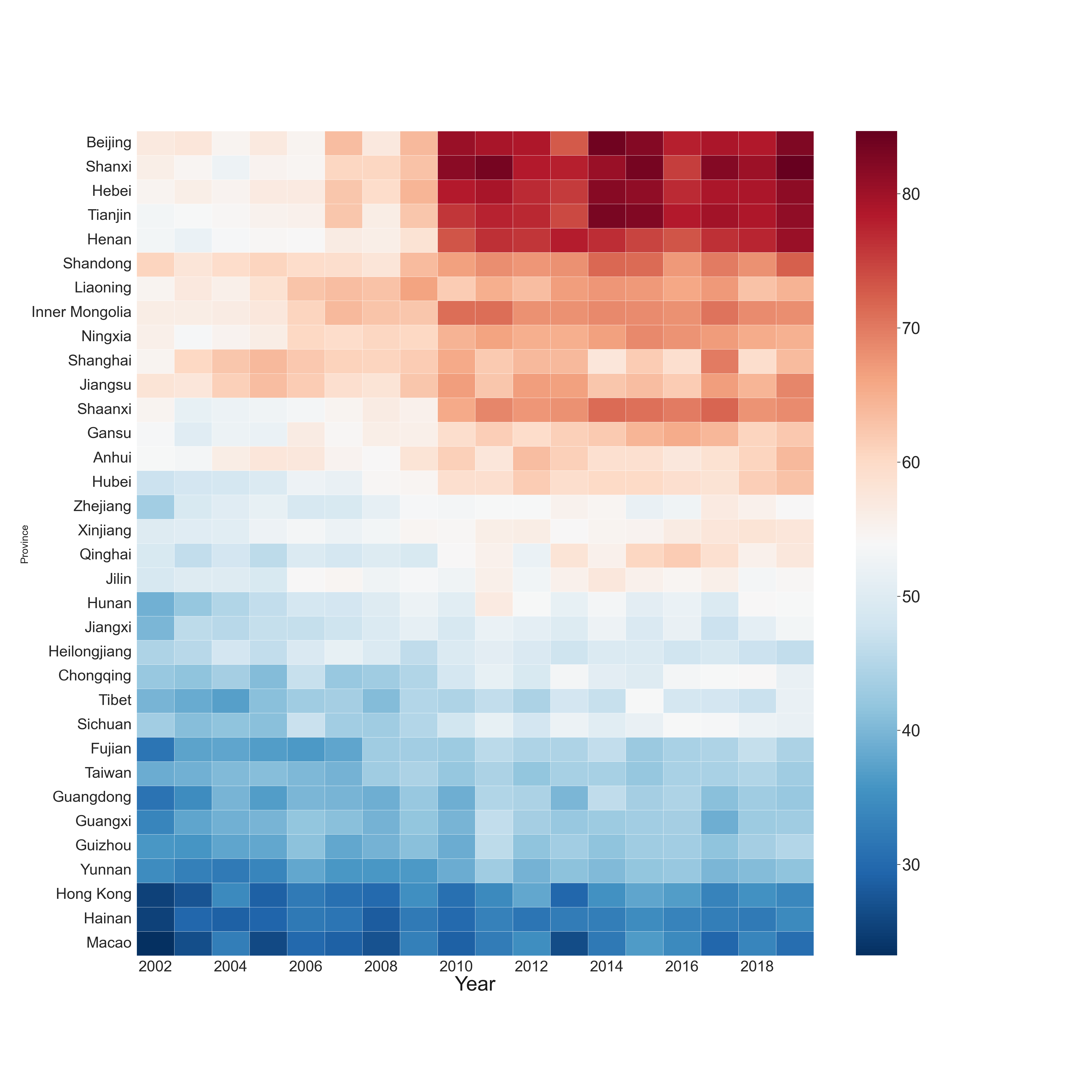 | 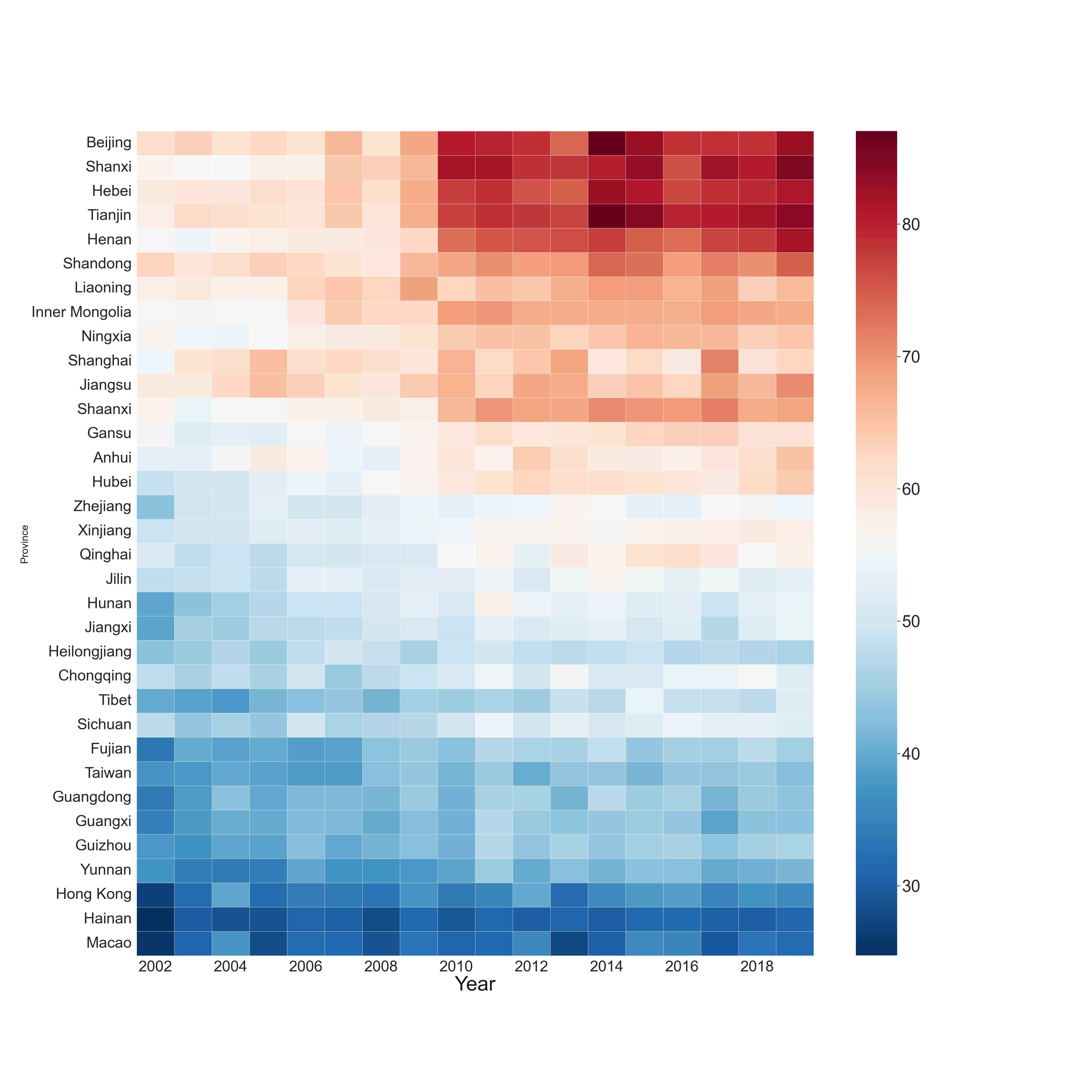 |
| G |  |
| 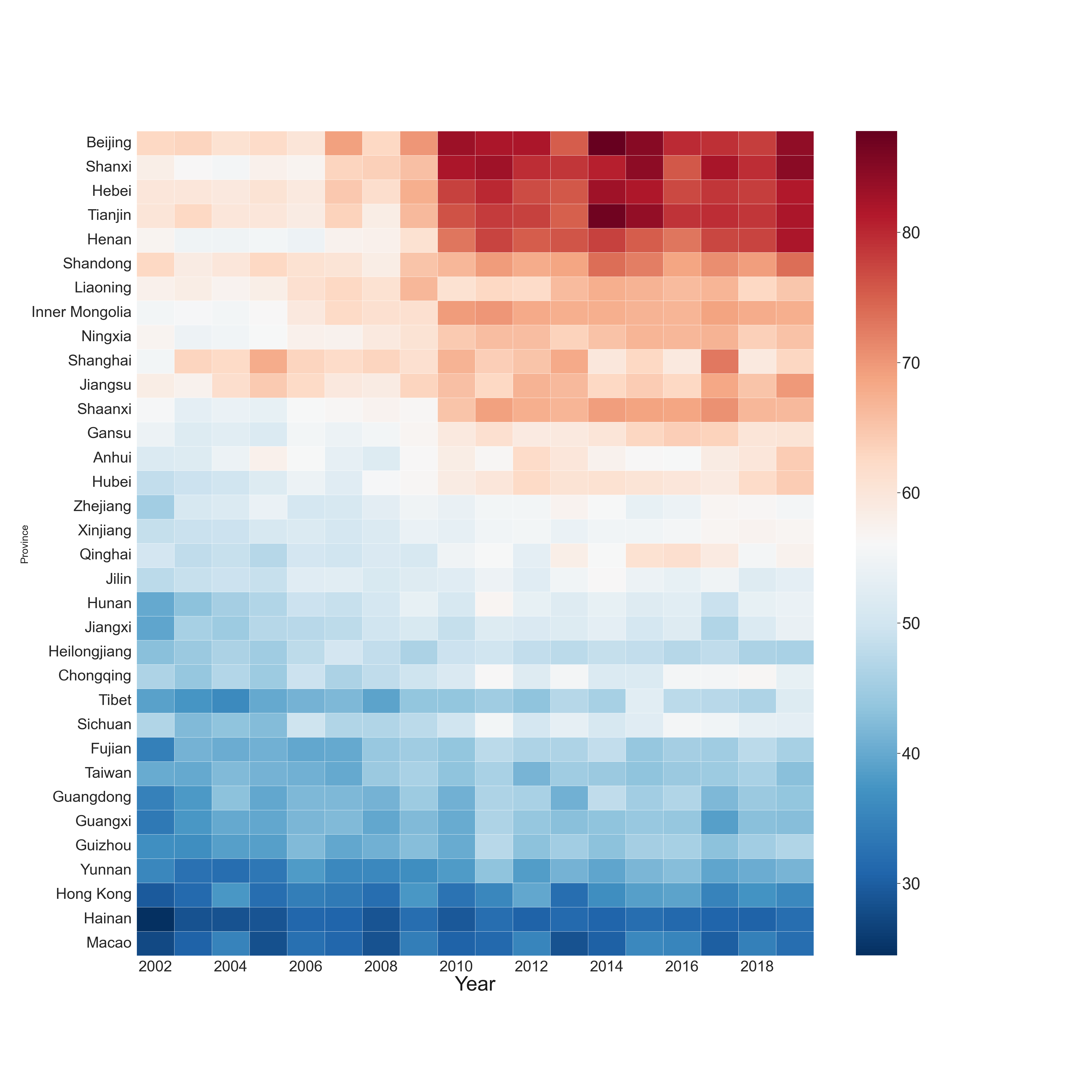 |  |

**Fig. S6.** Province-level spatiotemporal trends of summer mean MDA8 O_3_ concentrations. Panels A, B and C show the predicted concentrations using the Super Learner model, the LightGBM model, and the XGBoost model, all based on the baseline machine learning model configurations. Panels D-G show the predicted concentrations under four uncertainty scenarios: (1) utilizing validated ground-level PM_2.5_ for predictions, (2) excluding NDVI and population density as predictors, (3) applying IDW for grid-level weather data after excluding stations within 10 km of city centers, and (4) applying IDW for grid-level weather data after excluding stations within 20 km of city center. These uncertainty analyses were conducted using the Super Learner model.

| 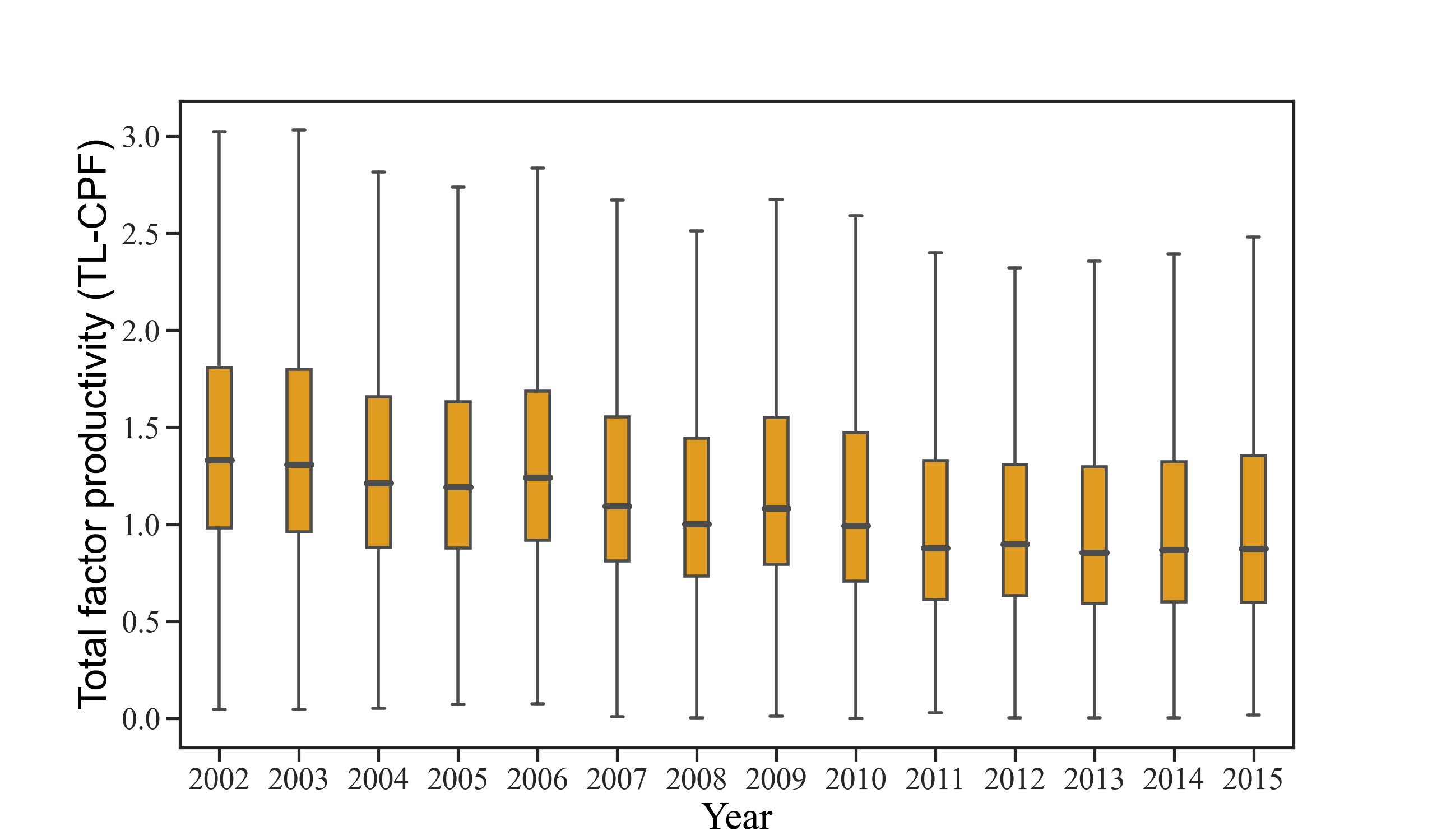 A | | 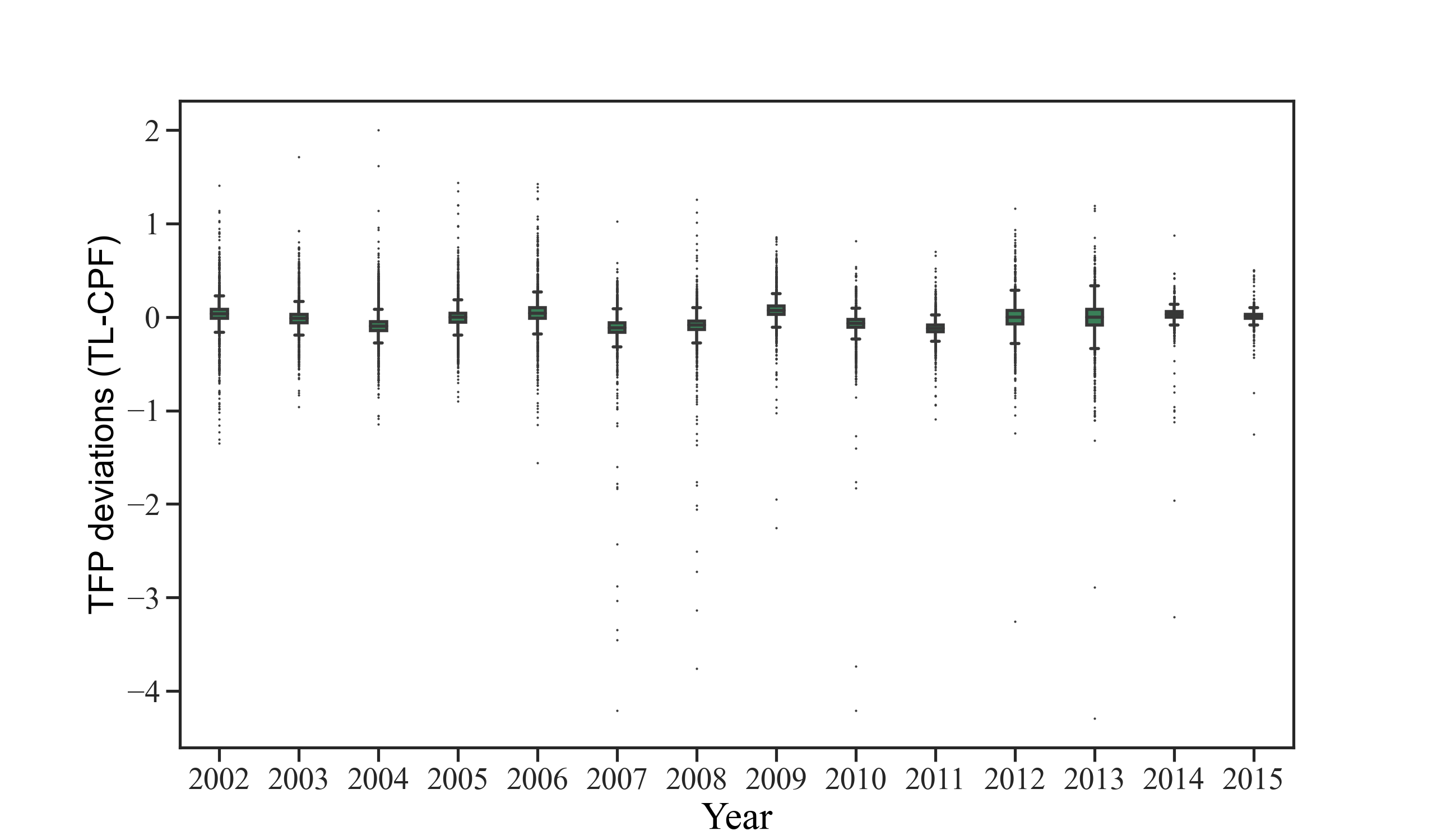 B |
| --- | --- | --- |
| 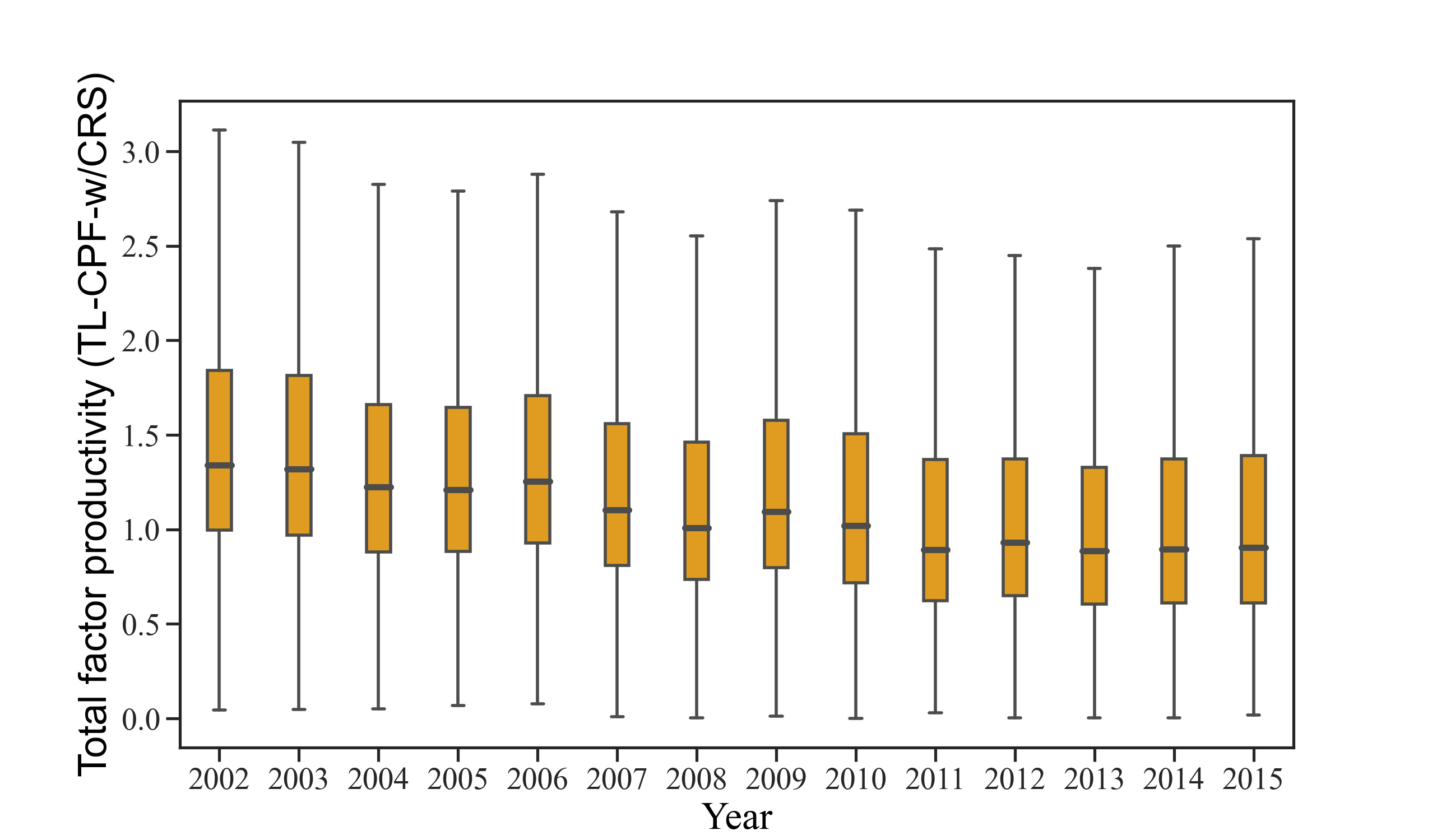 | | 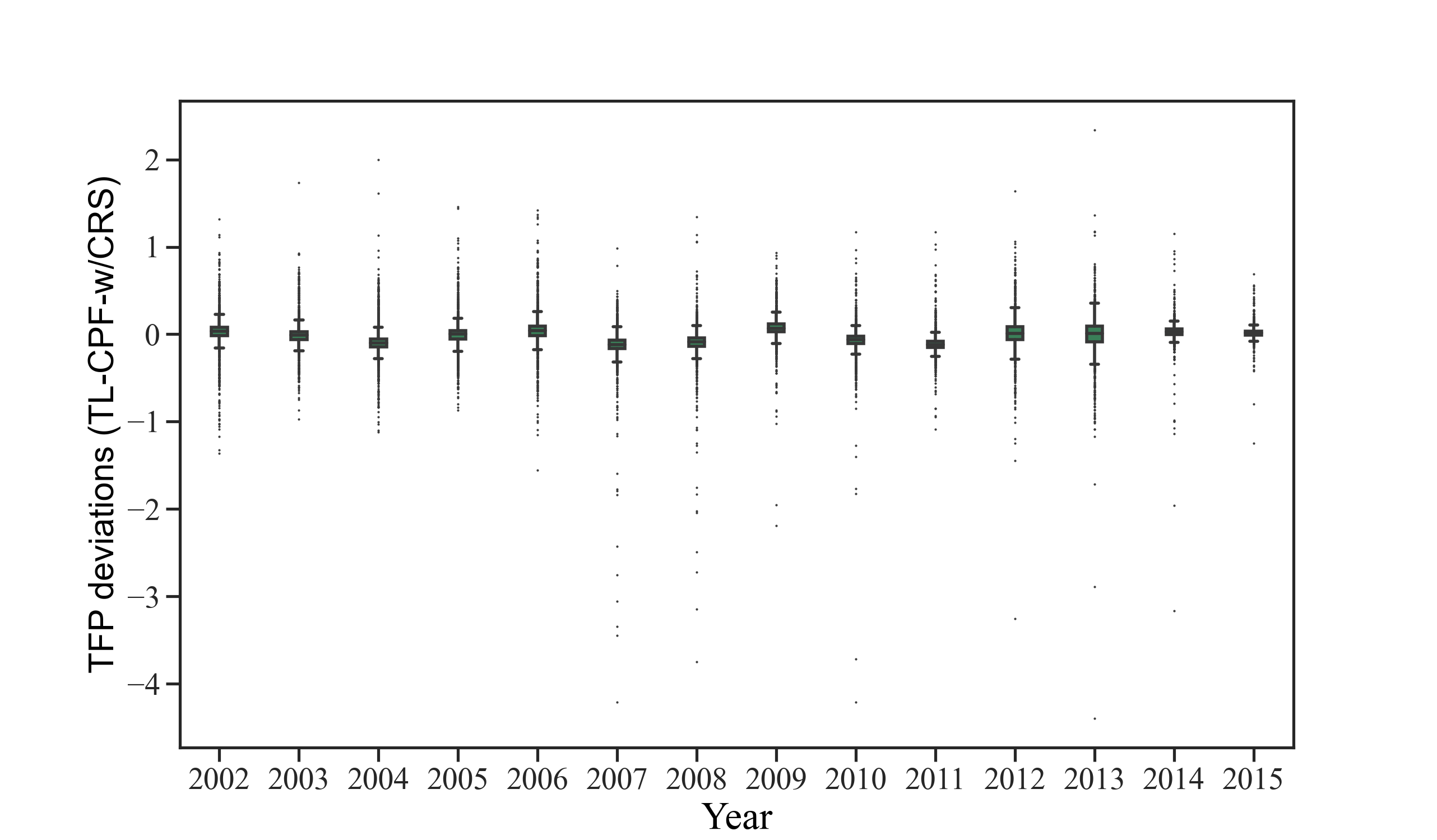 |
| 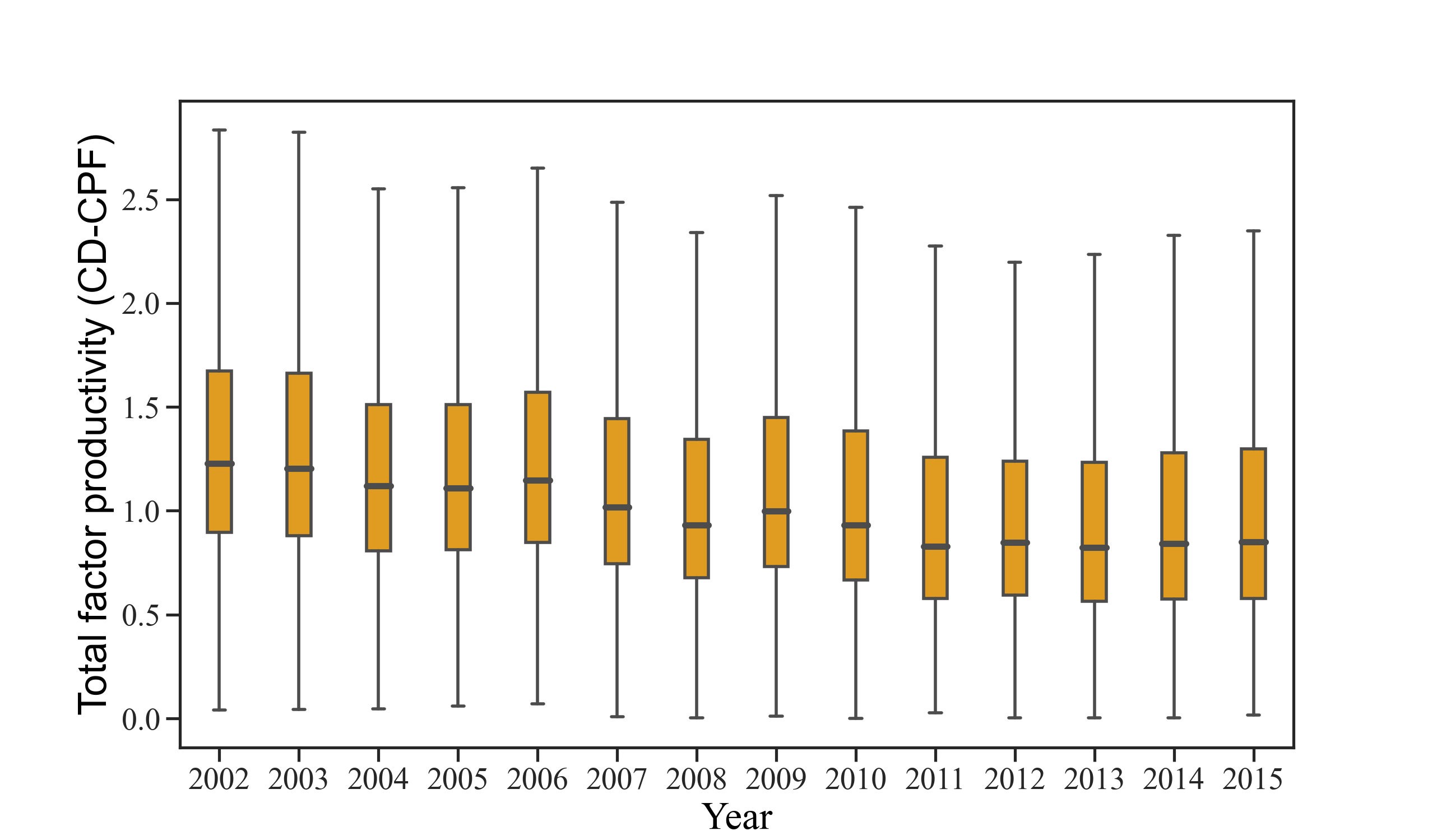 | | 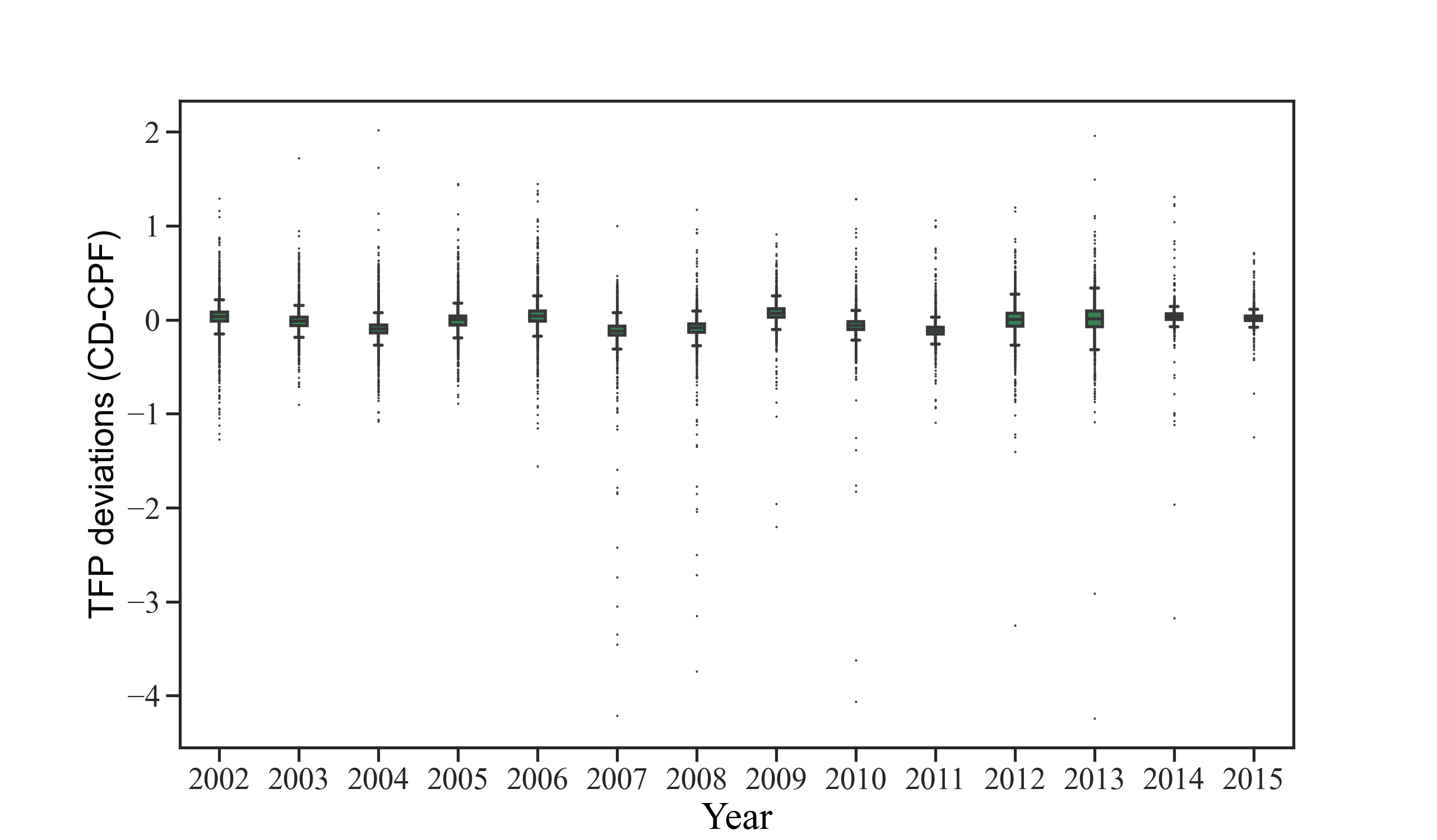 |
| 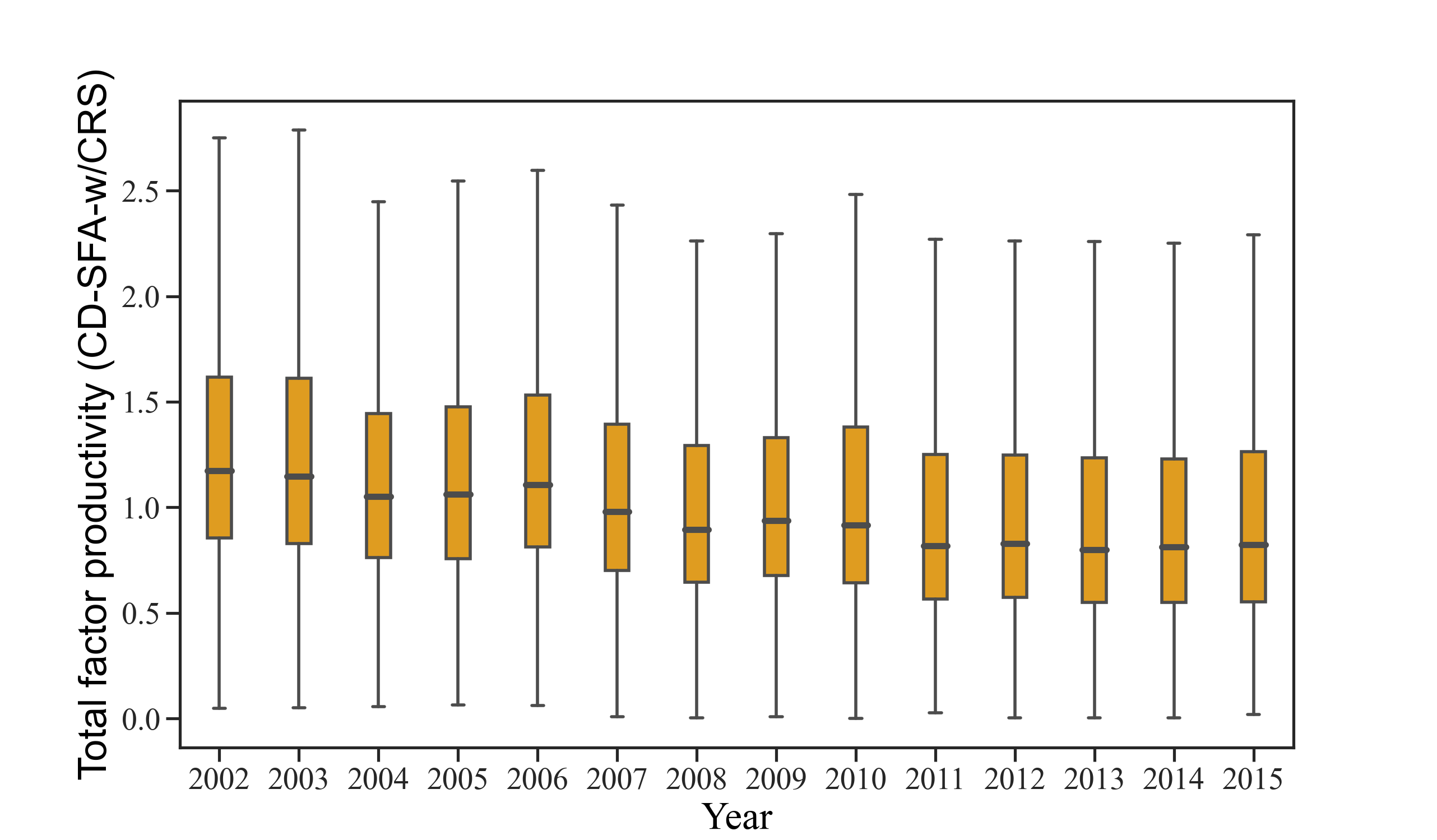 | 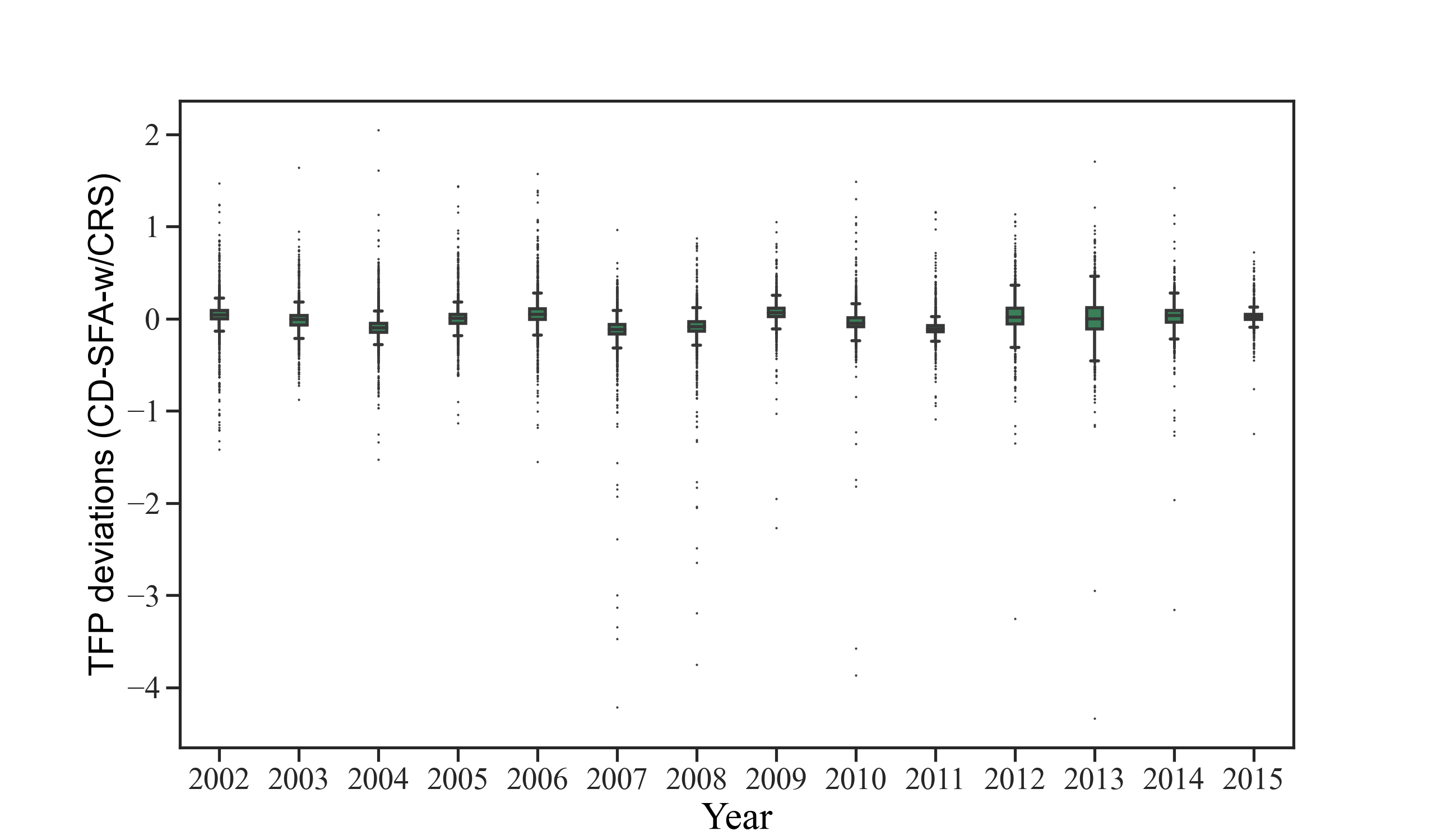 | |

**Fig. S7.** Distribution of agricultural TFP during 2002-2015 for TFP derived from the TL-CPF, TL-CPF-w/CRS, CD-CPF and CD-SFA-w/CRS models. (A) Distribution of China’s TFP over 2002-2015. The box represents the first three quartiles (Q1, Q2, and Q3) and whiskers extend to 1.5 times the interquartile range (IQR=Q3-Q1). TFP is normalized to 1 for Chaohu county in Anhui province in 2002. (B) Distribution of first differences in the log of county-level TFP. The box represents the first three quartiles and whiskers extend to 1.5 times the IQR. Observations that fall beyond 1.5 IQR are represented with small circles.

| A | B |
| --- | --- |
| 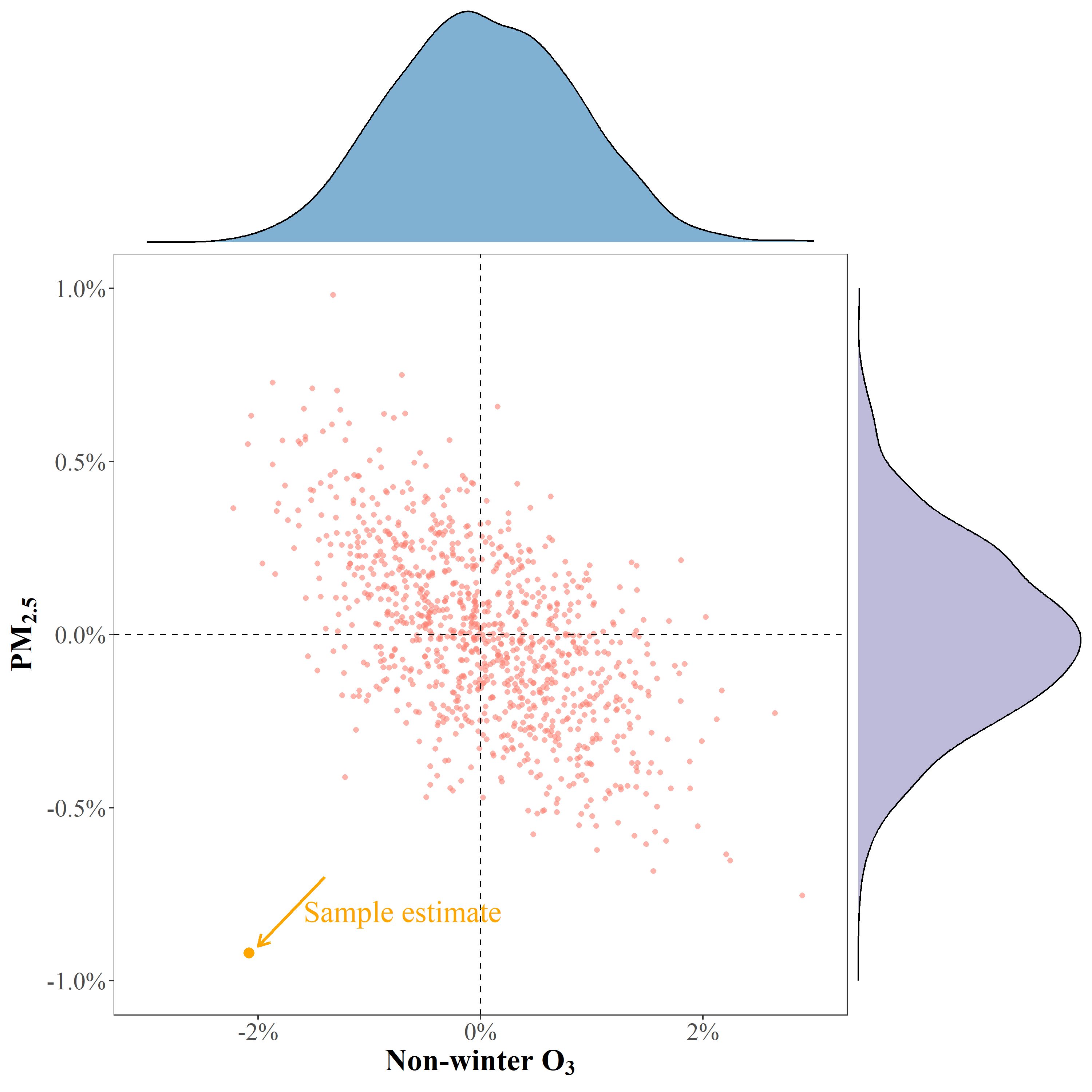 | 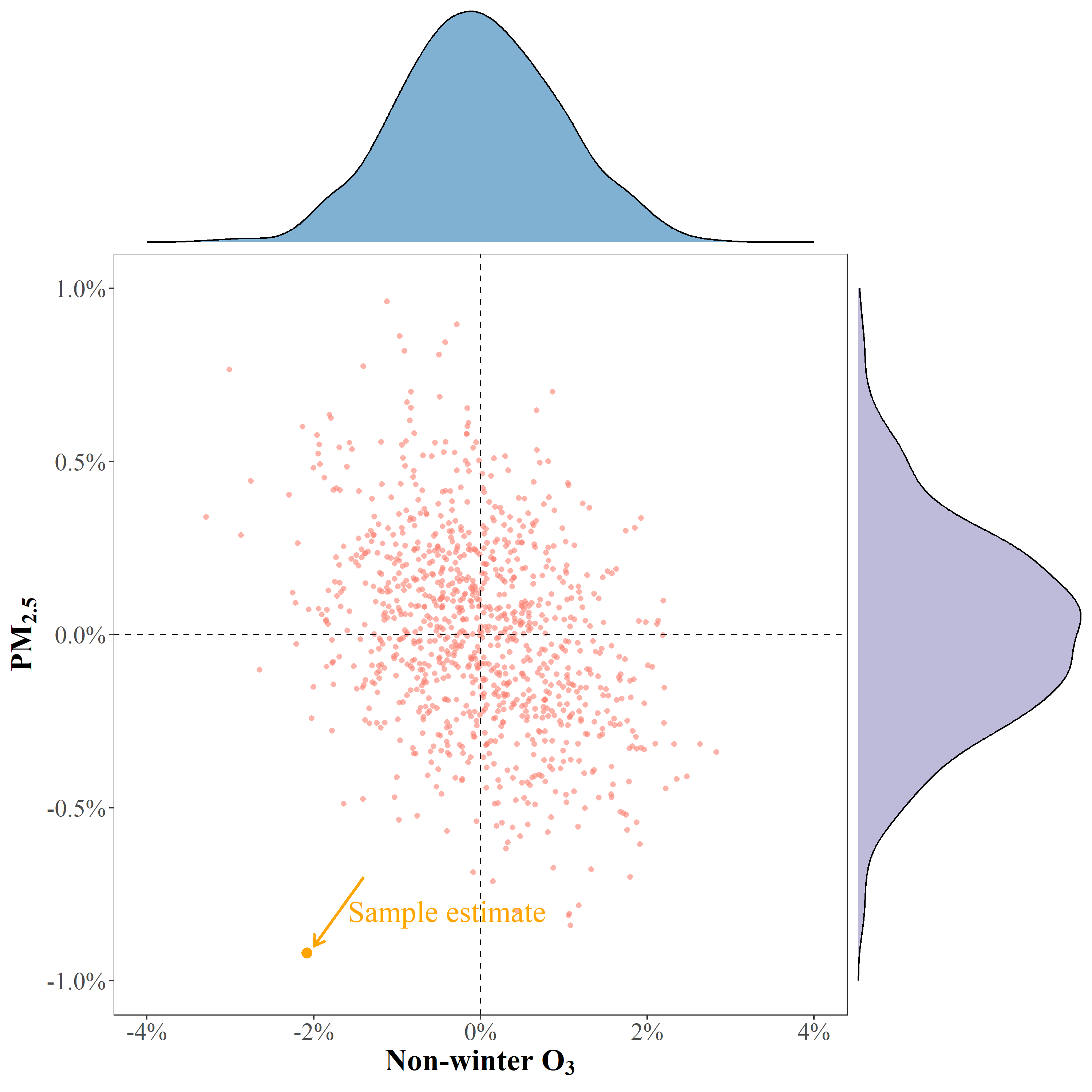 |

**Fig. S8.** Distribution of placebo estimates. In panel A, we randomly mismatched the county-year TFP and pollution data (including O_3_ and PM_2.5_) 1,000 times. In panel B, TFP and pollution data were randomized 1,000 times within seasons and regions. Specifically, we first mismatched seasonal O_3_ concentrations within individual counties and computed both winter and non-winter O_3_ concentrations for each county. We then randomly shuffled the county-year TFP and pollution data within each regional cluster. The orange dot in each panel represents our baseline estimates. In both panels, the upper blue figures represent the distribution of non-winter O_3_ estimates obtained from 1,000 placebo tests, while the right-side purple figures represent the corresponding distribution of PM_2.5_ estimates.

| 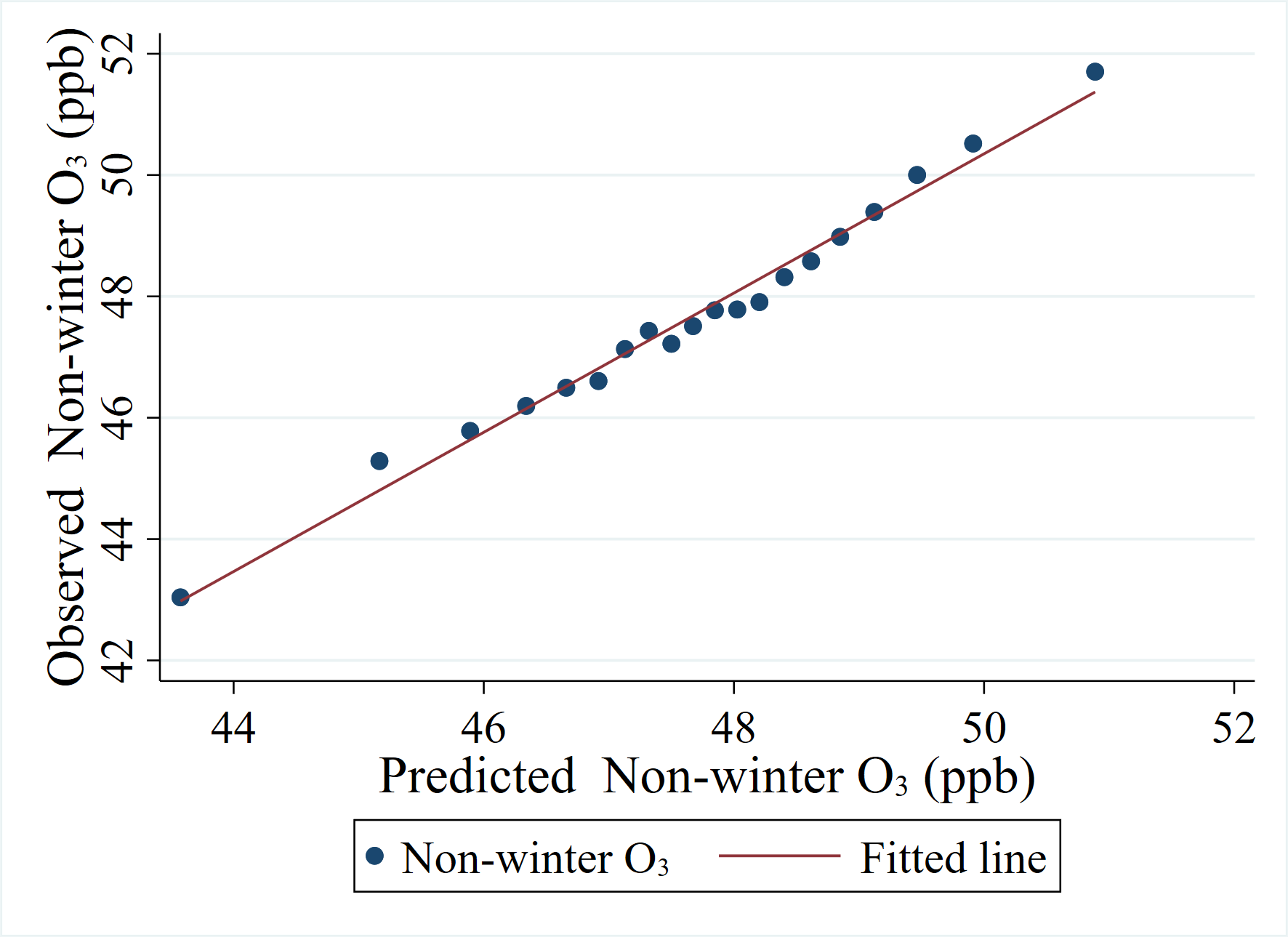 (a) | 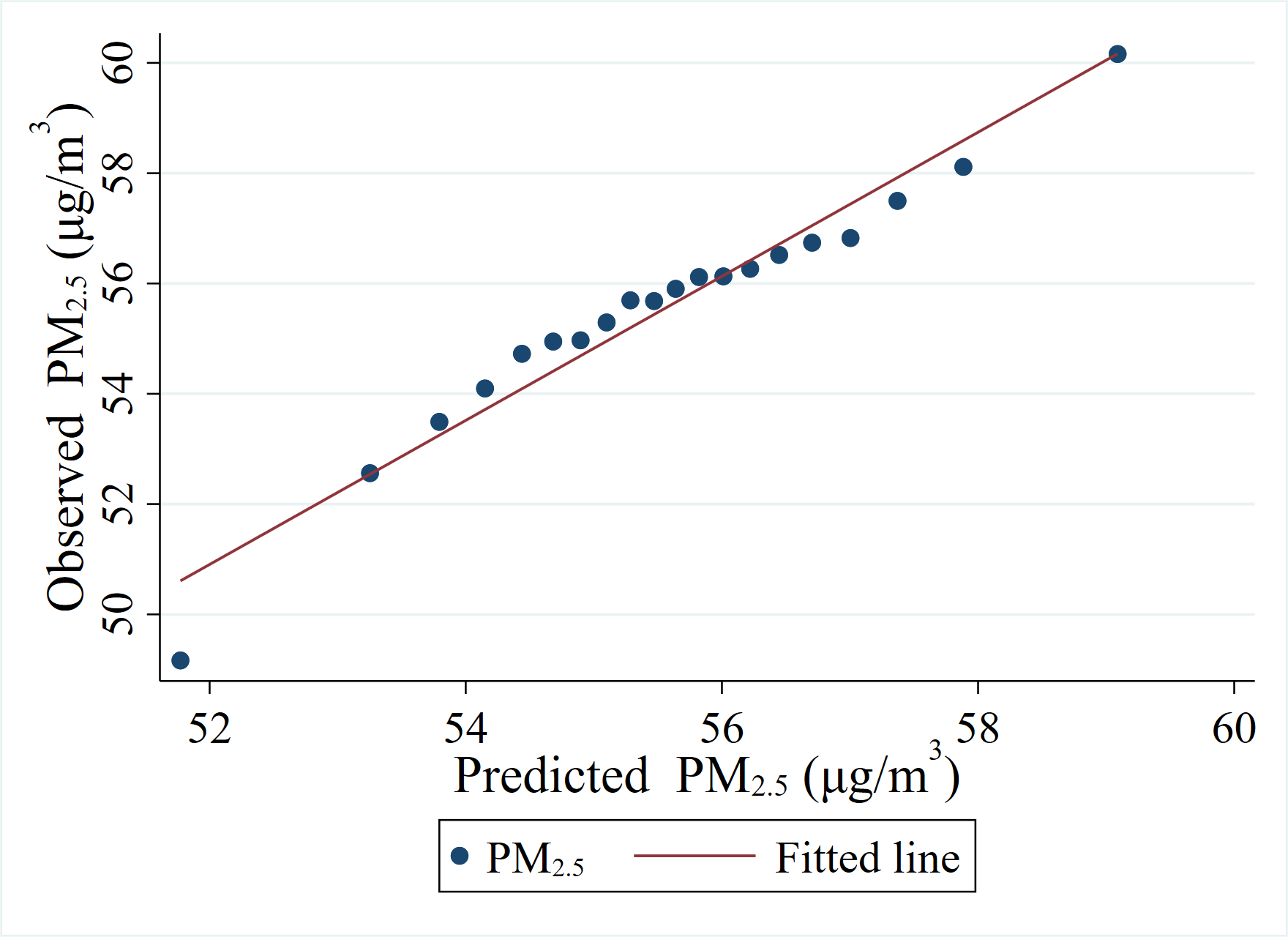(b) |
| --- | --- |

**Fig. S9.** Predicted and observed pollution levels. These figures show the observed non-winter O_3_ and PM_2.5_ levels plotted against the predicted non-winter O_3_ and PM_2.5_ levels generated by our first-stage model (Eq. 2). Weather controls and fixed effects have been included as is stated in the baseline specification. The data points correspond to the mean values of the predicted and observed pollution concentrations within each of the 20 quantiles. In each figure, the red line denotes the estimated fitted line from a linear regression of observed values on predicted values, with county fixed effects.

|  | A B C | D |
| --- | --- | --- |
| (1) TL-CPF | 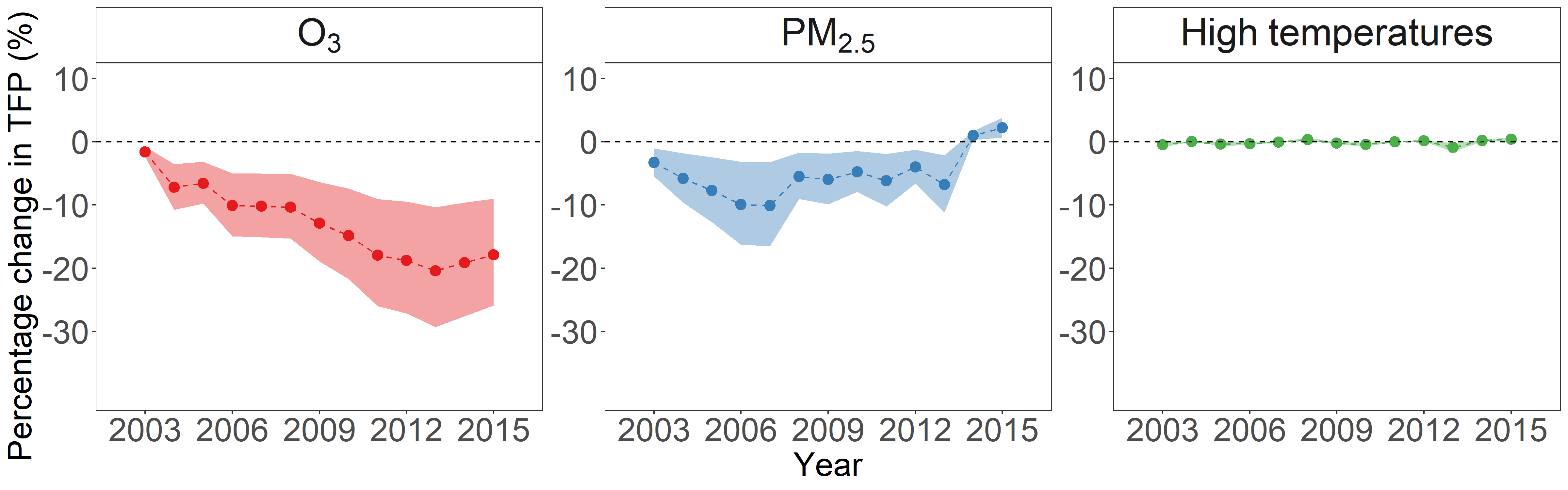 | 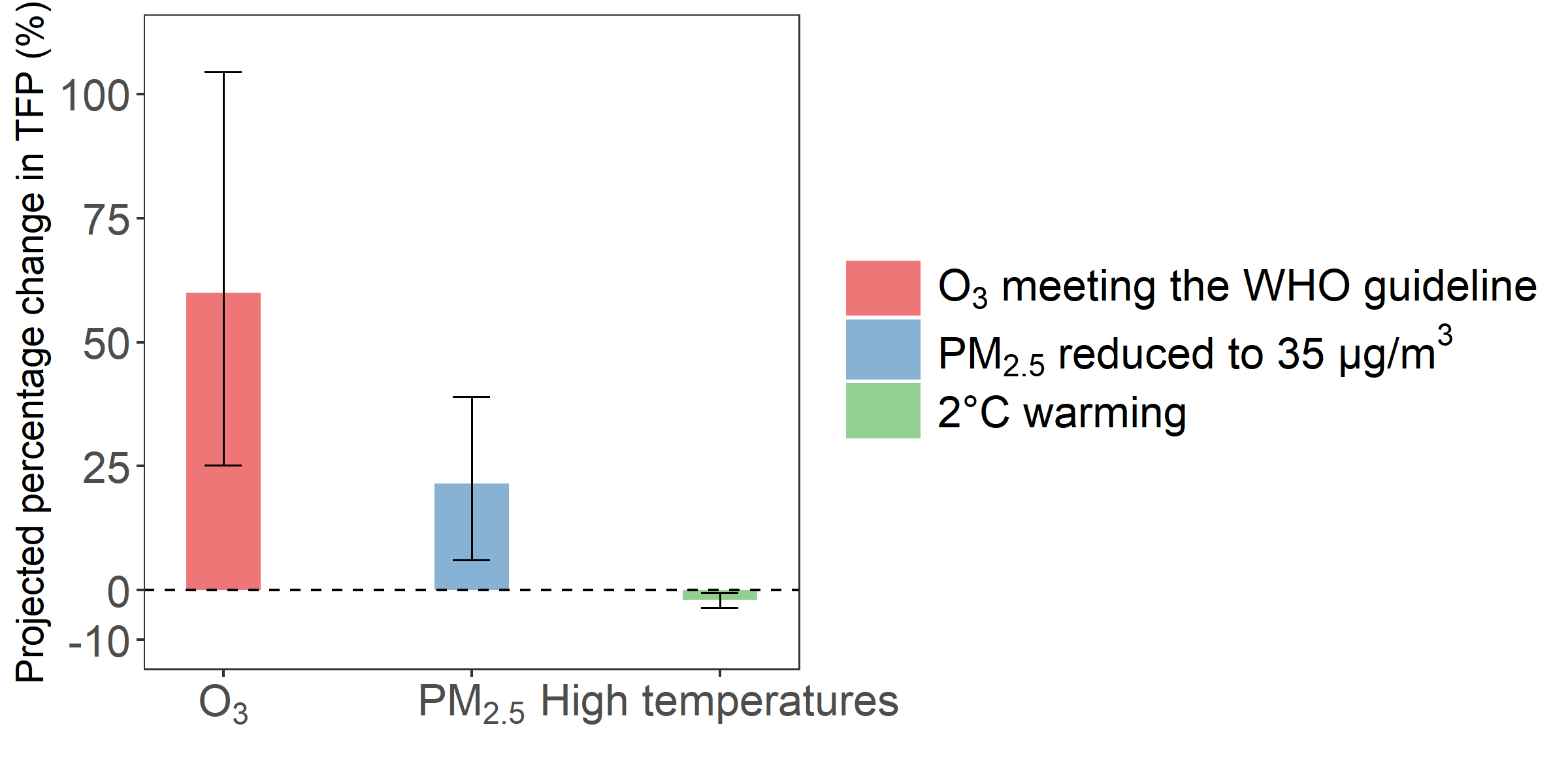 |
| (2) TL-CPF-w/CRS | 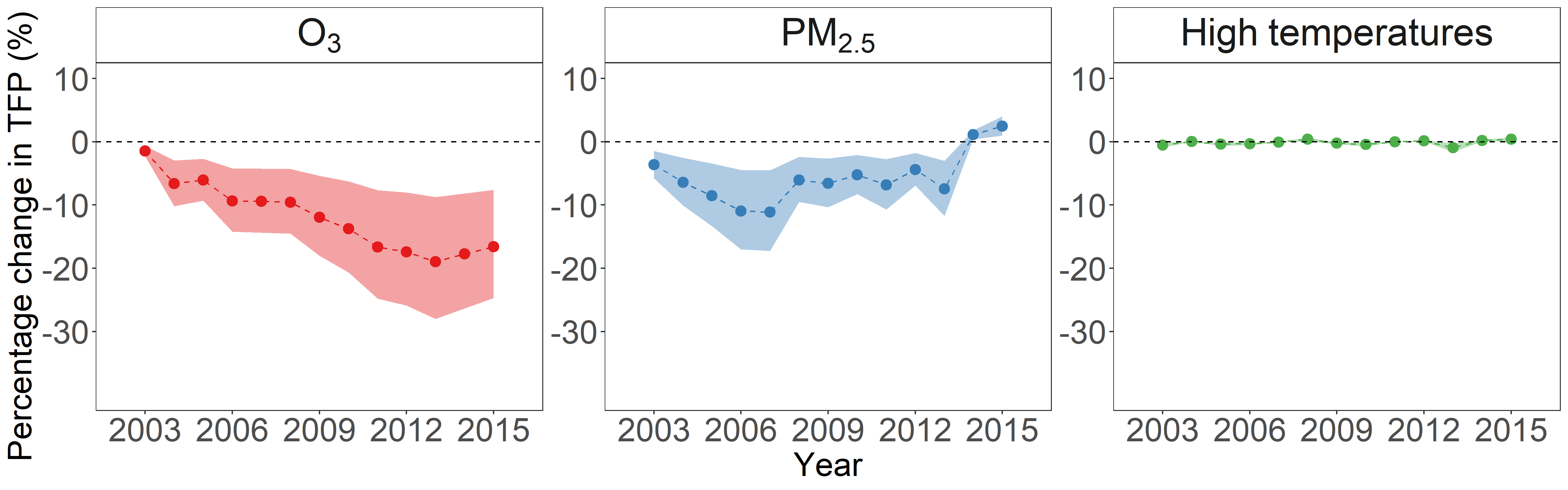 | 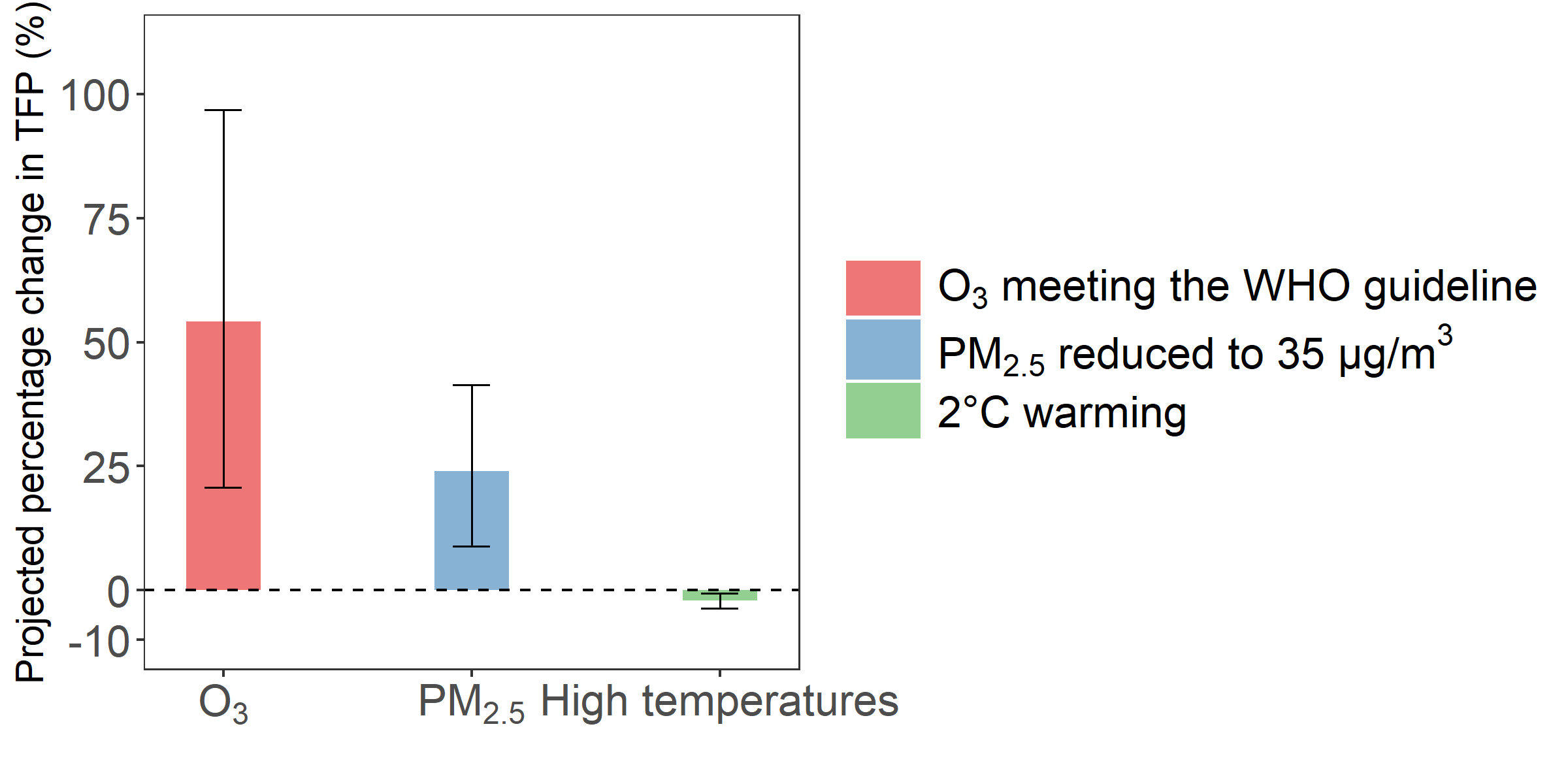 |
| (3) CD-CPF | 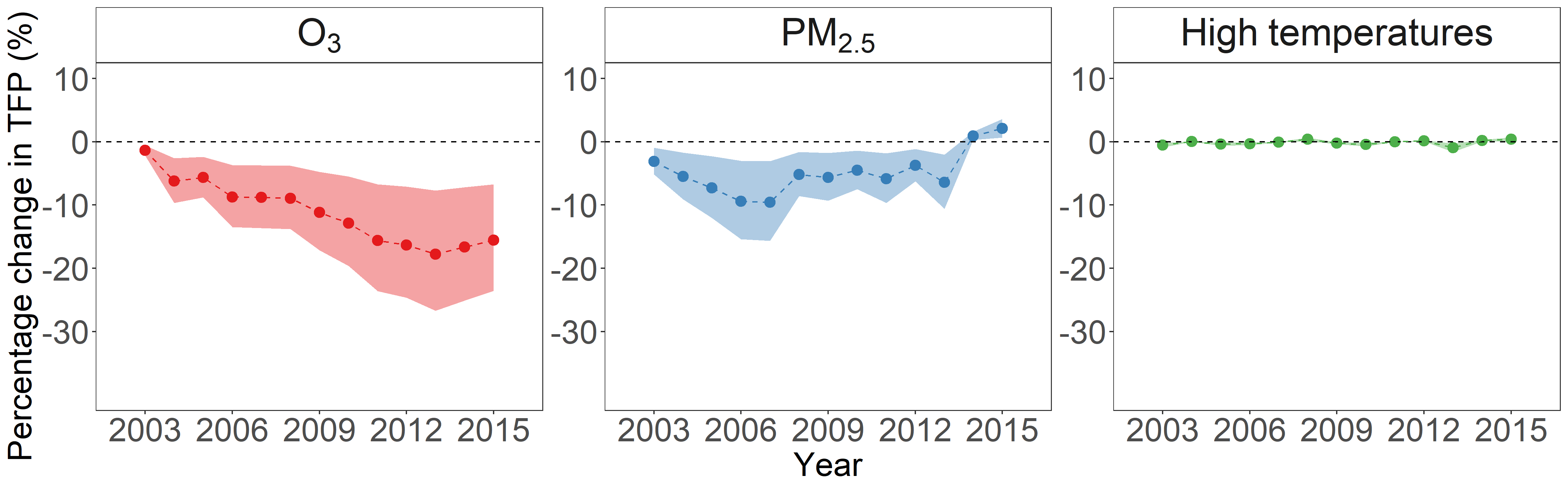 | 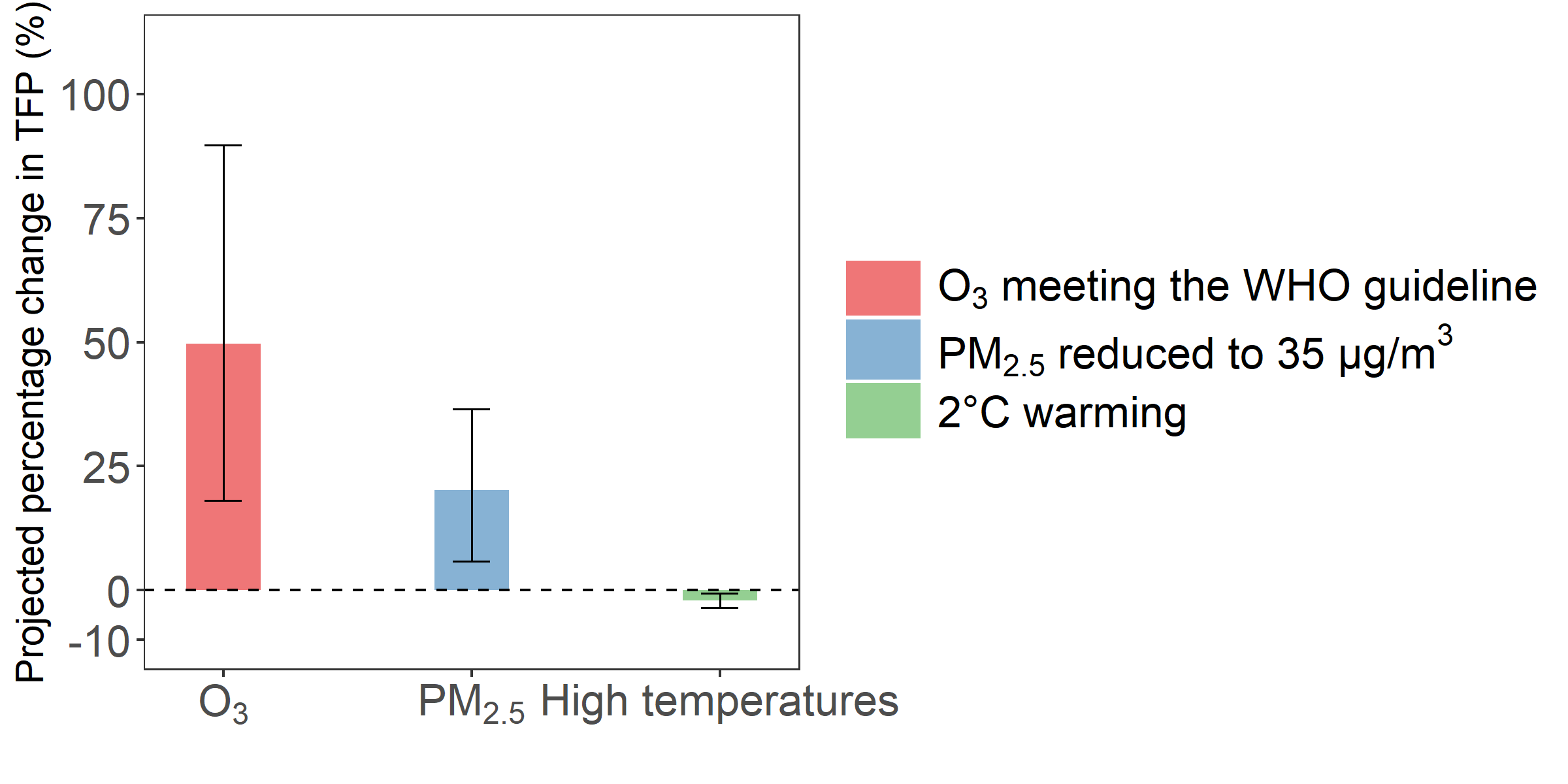 |
| (4) CD-SFA-w/CRS | 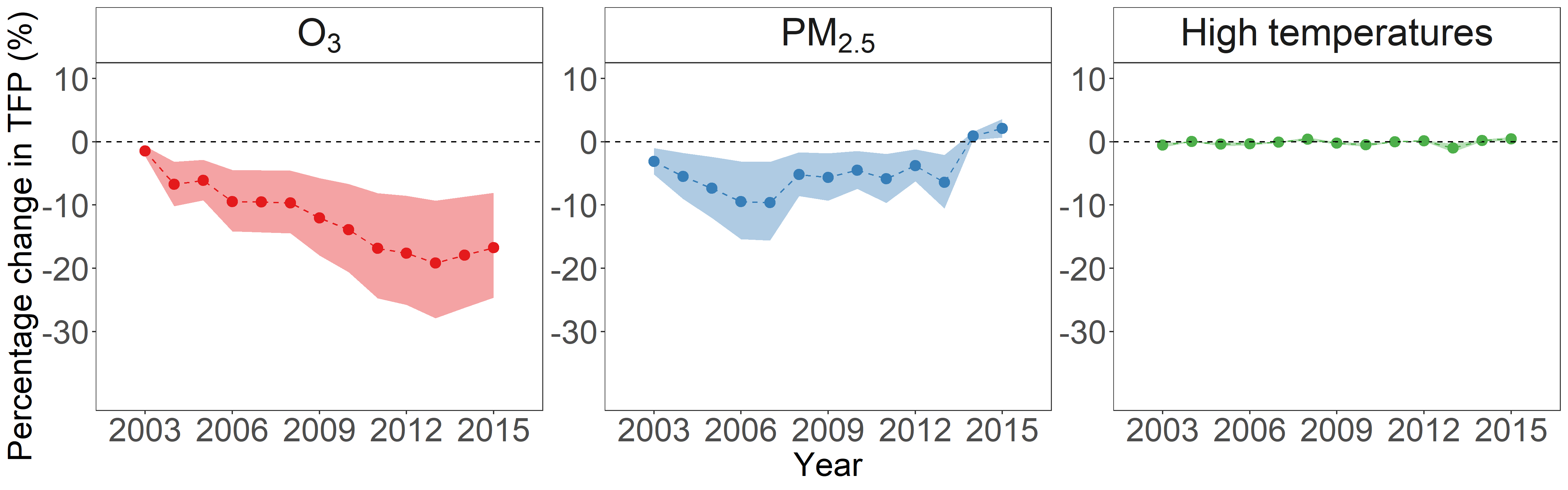 | 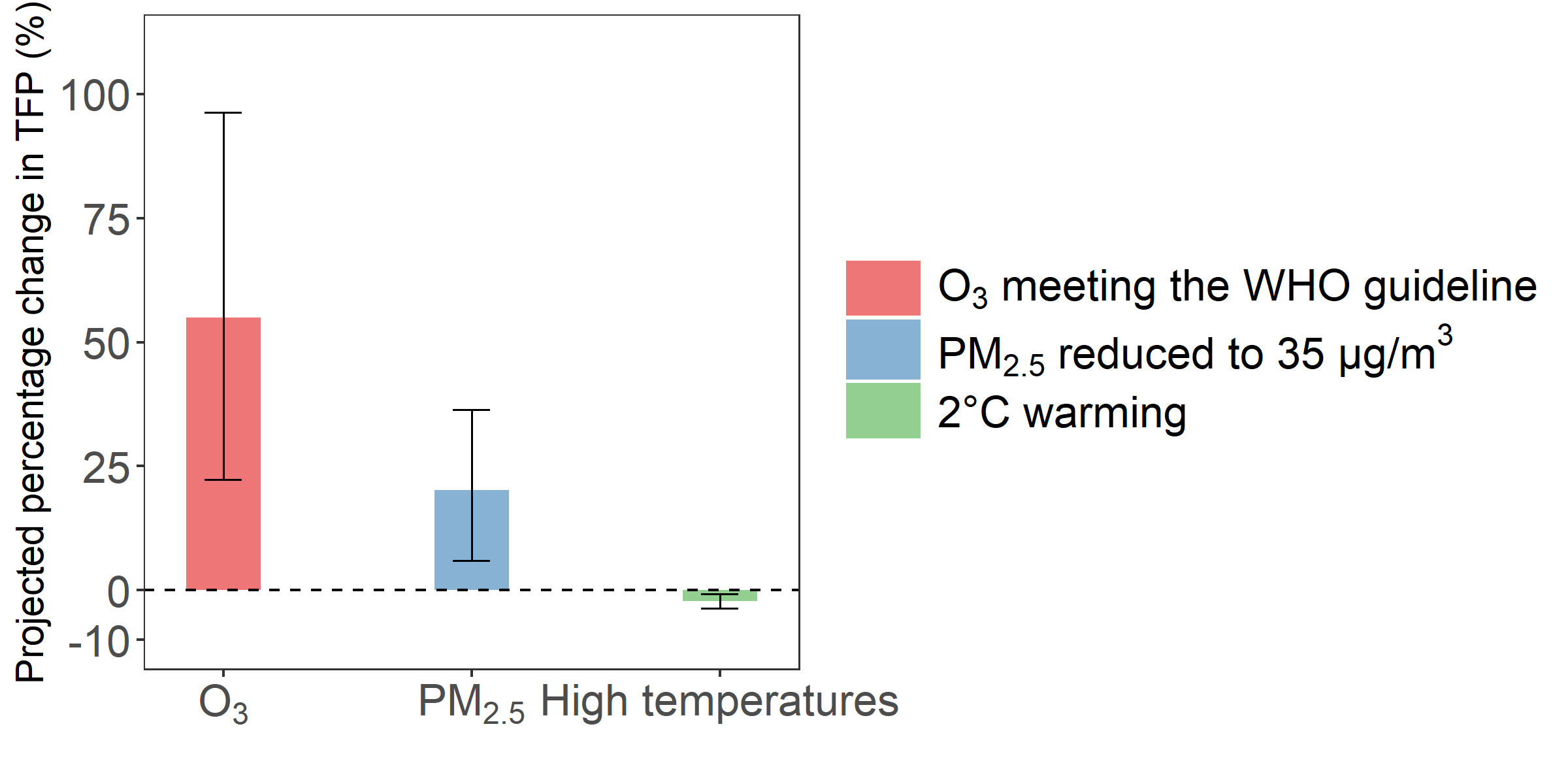 |
| (5) Labor  Productivity | 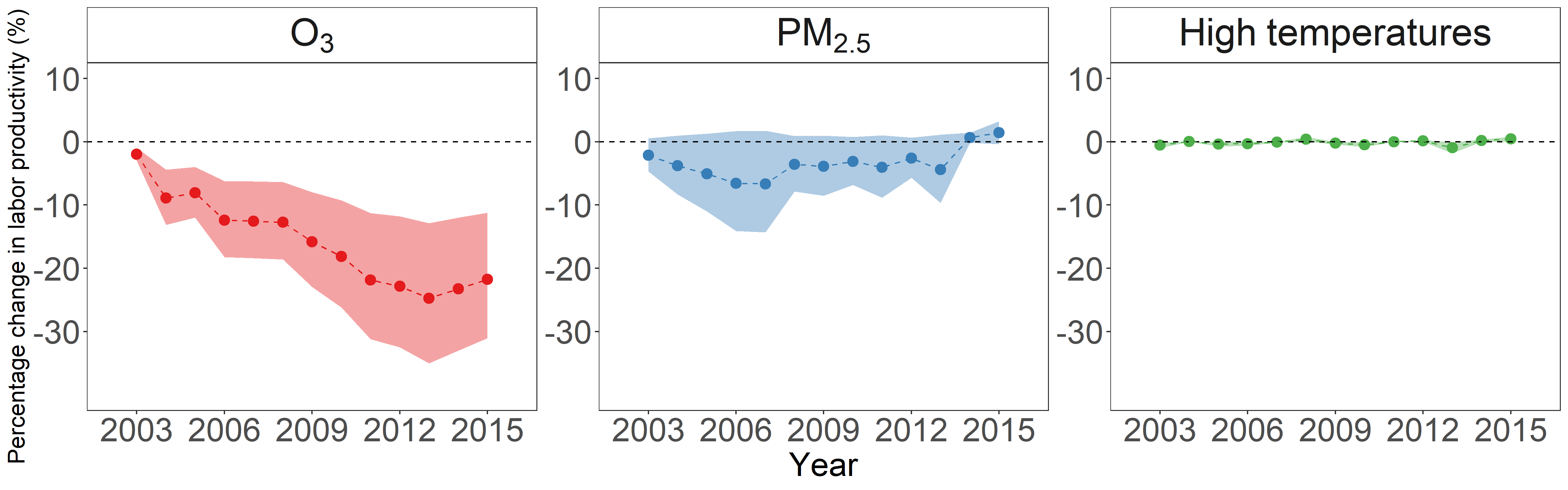 | 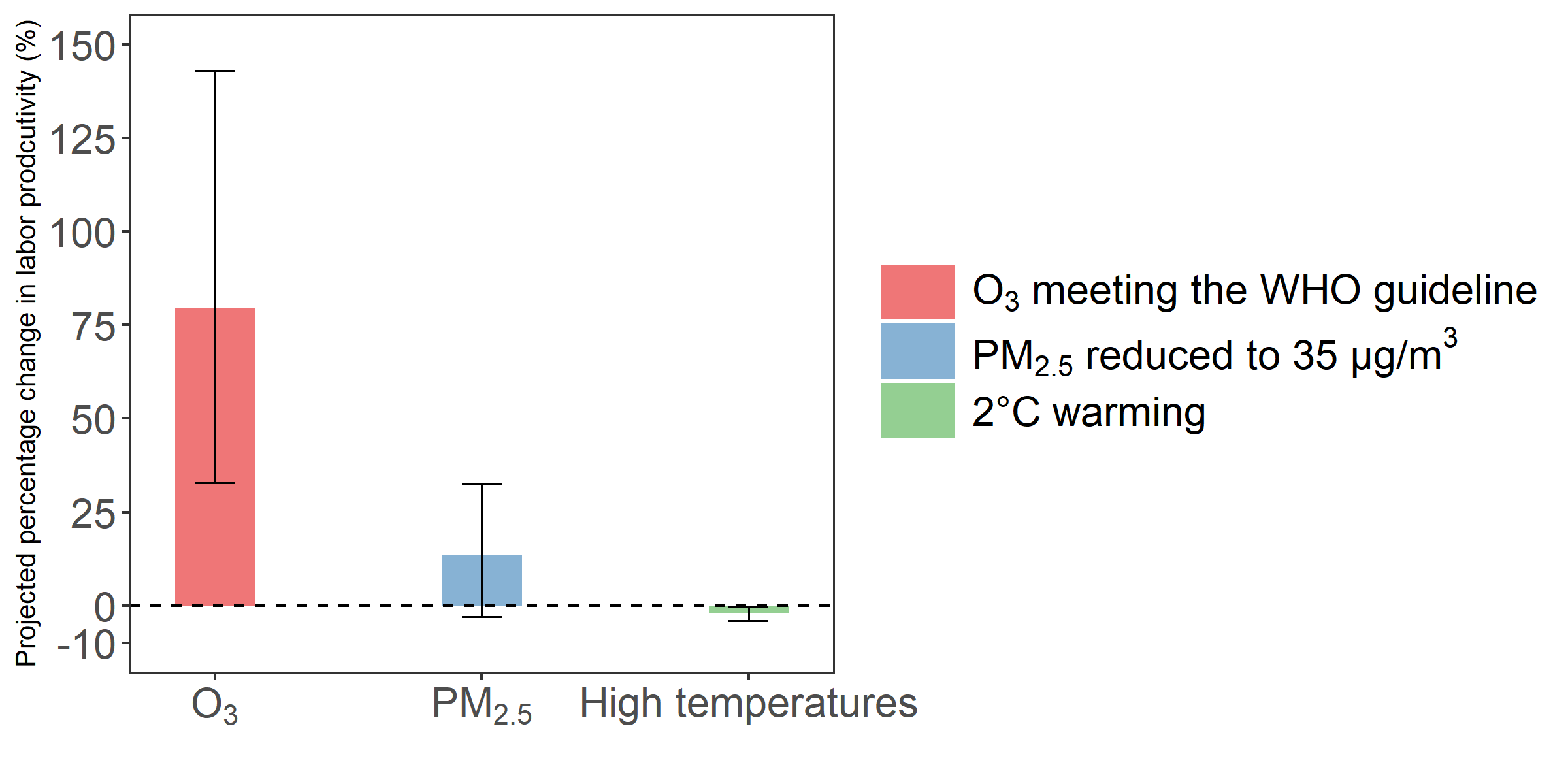 |

**Fig. S10.** Estimated agricultural productivity changes due to pollution and high temperatures. Panels A-C show estimated changes in TFP resulting from variations in non-winter O_3_ concentrations, PM_2.5_ and days with high temperatures above 35°C, respectively, for the years 2003-2015. Productivity changes were calculated by using Eq. (1) to predict TFP under two conditions: (*i*) using historical, observed values of O_3_, PM_2.5_ and days with high temperatures above 35°C for each year between 2002 and 2015, and (*ii*) hypothetical scenarios with each of these factors held at their 2002 levels. Each point is a weighted mean of percentage changes in county-level TFP between the two conditions, where the value of a county was weighted by its total output value. The black, dashed, horizontal line marks 0 change for reference. The shallow bands in each panel are 95% confidence intervals. Panel D shows the projected changes in agricultural TFP from hypothetical pollution reductions and a scenario of 2℃ warming, in which daily temperatures across all counties would uniformly increase by 2°C relative to the 2015 levels. The length of a bar shows the projected percentage change due to a given factor relative to 2015, and the whiskers are 95% confidence intervals for the estimates. Rows 1-4 show the results using TFP derived from the TL-CPF, TL-CPF-w/CRS, CD-CPF and CD-SFA-w/CRS models, respectively. The last row shows the results using labor productivity as the productivity measure.

| 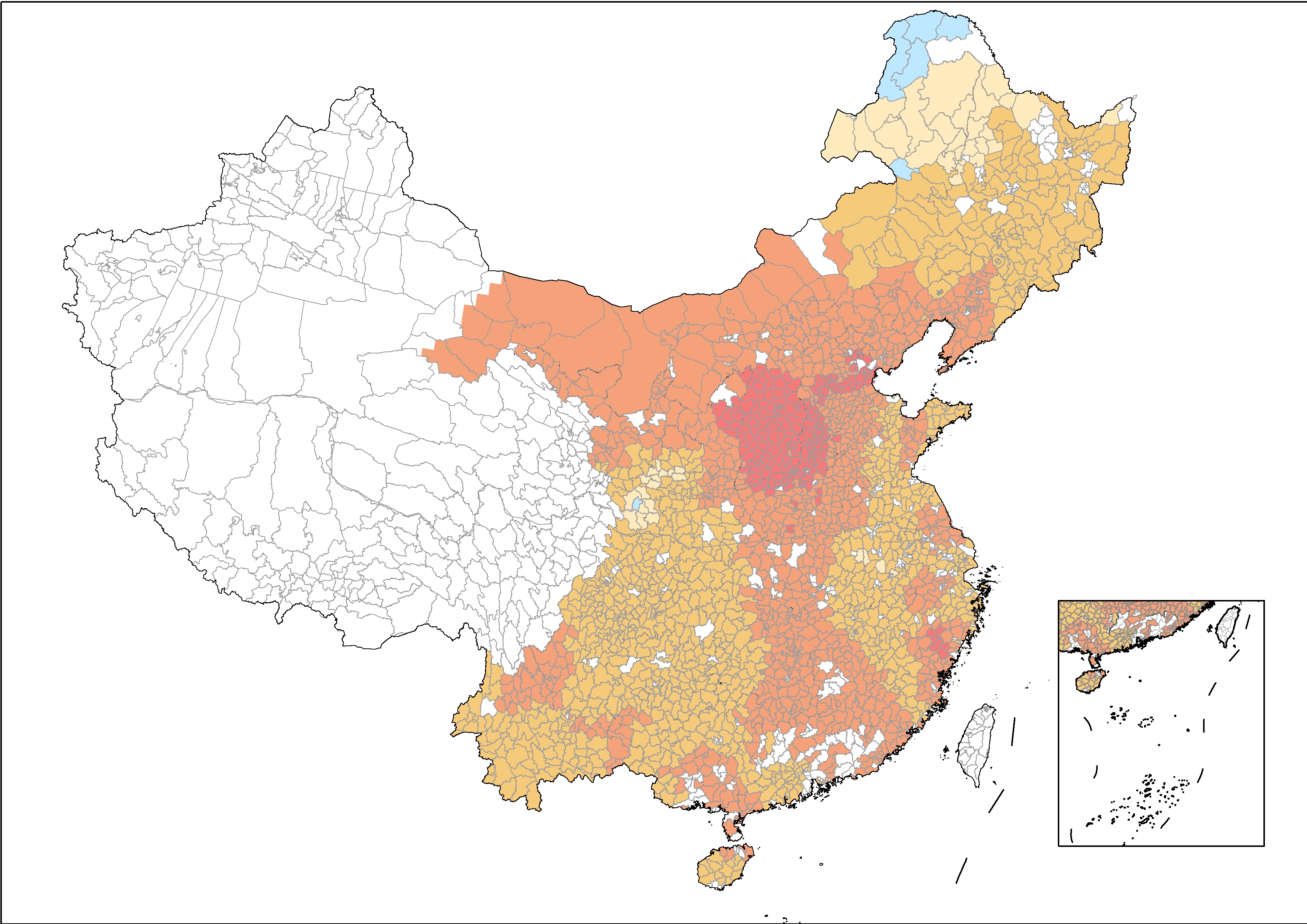(A) Non-winter O_3_ | 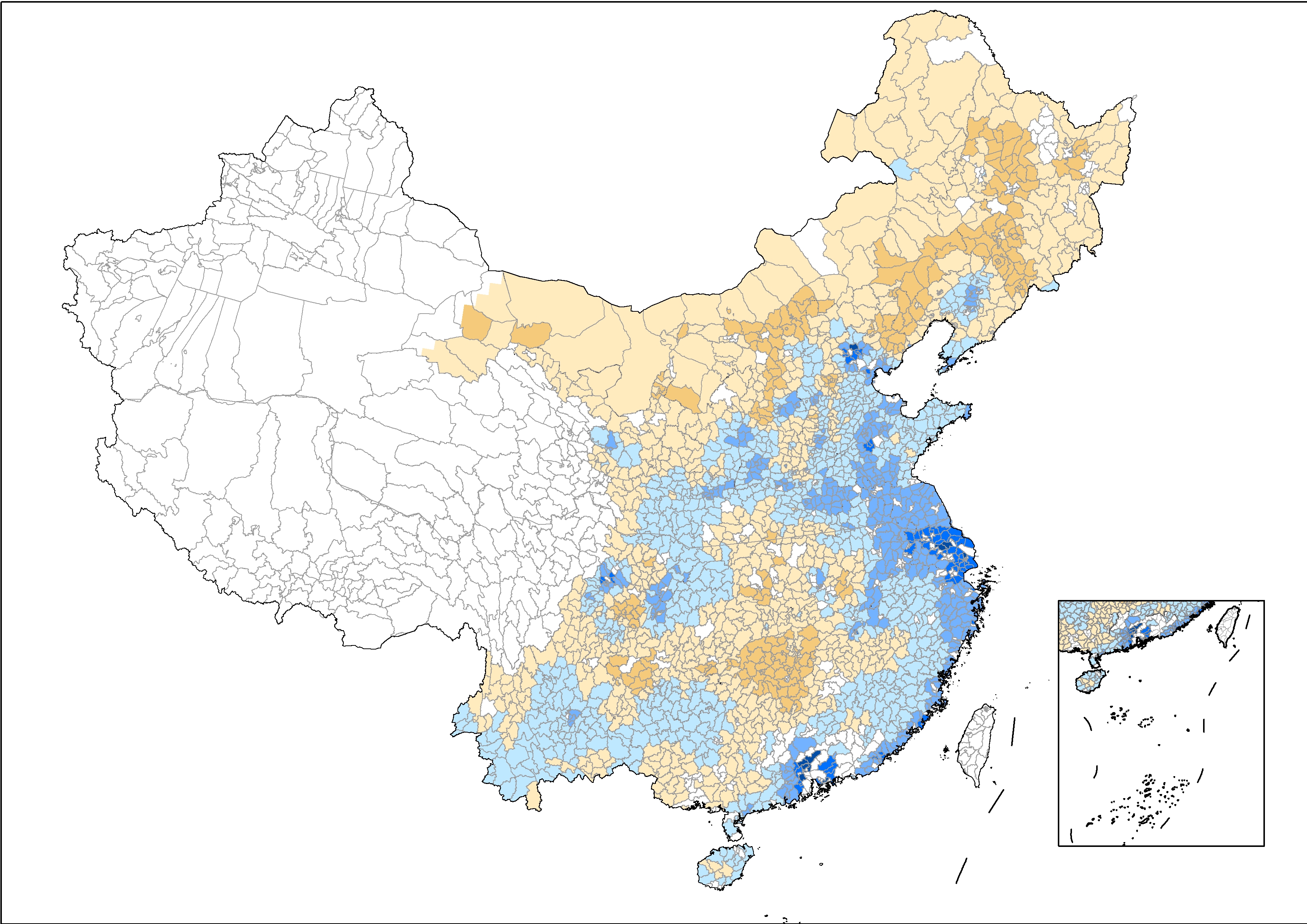(B) PM_2.5_ | 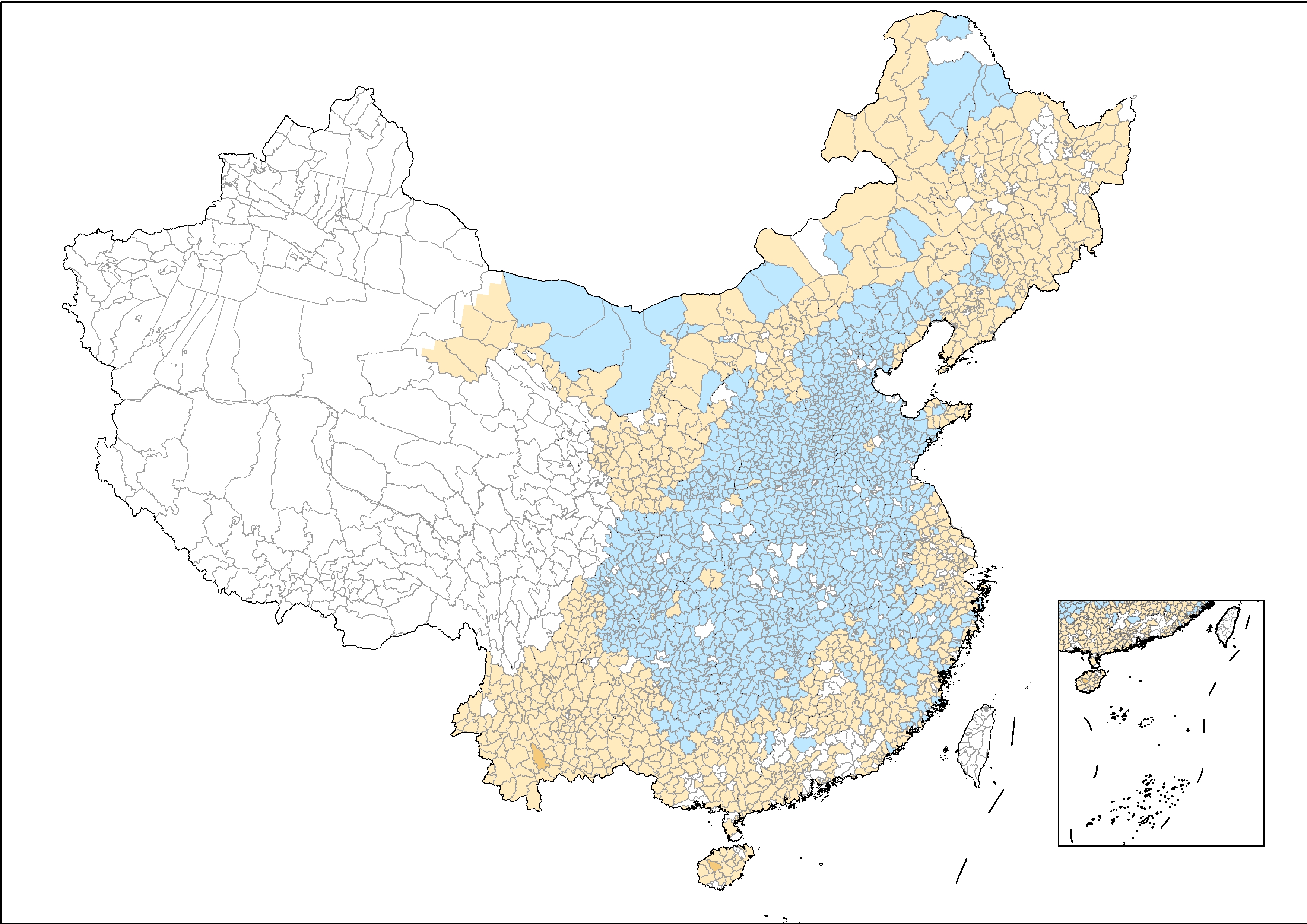(C) Temperatures ≥ 35°C | 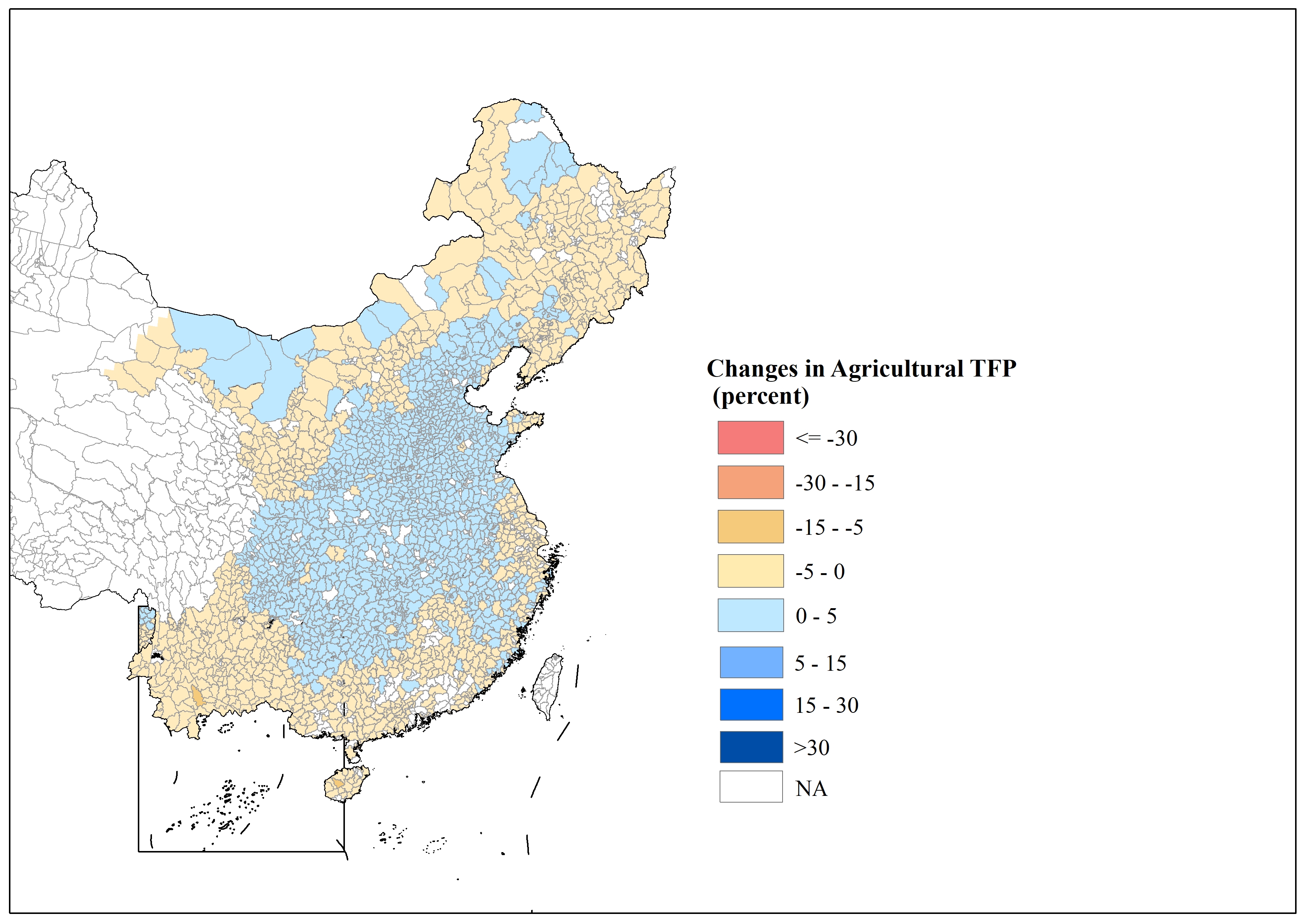 |
| --- | --- | --- | --- |

**Fig. S11.** Productivity changes due to pollution and high temperatures between 2002 and 2015. These maps show estimated percentage changes in county-level TFP due to changes in non-winter O_3_ (A), PM_2.5_ (B) and high temperatures above 35°C (C) for year 2015 relative to their levels in 2002. Productivity changes were calculated by using Eq. (1) to predict TFPs using historical, observed values of O_3_, PM_2.5_ and days with high temperatures above 35°C in 2015 and their values in 2002.

| Annual | 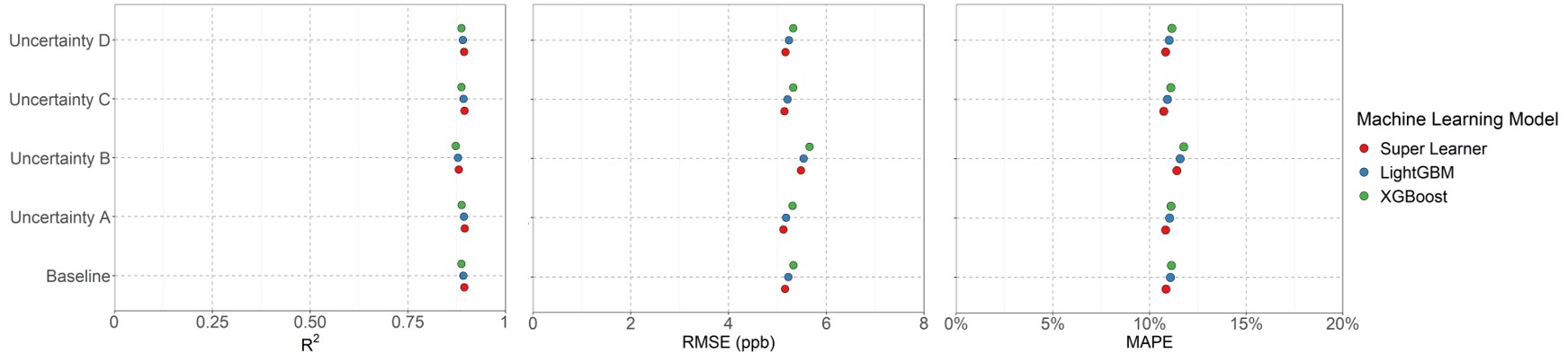 |
| --- | --- |
| Spring | 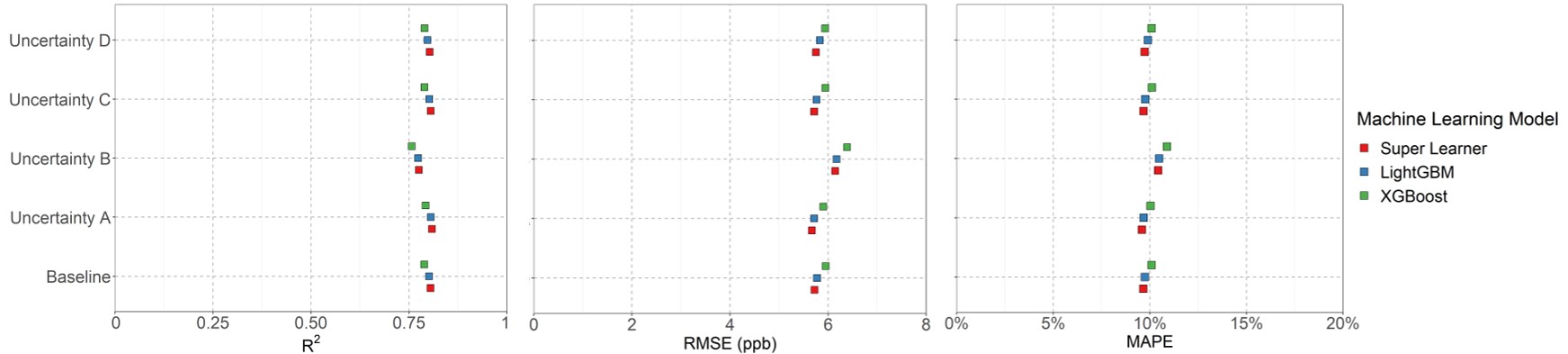 |
| Summer | 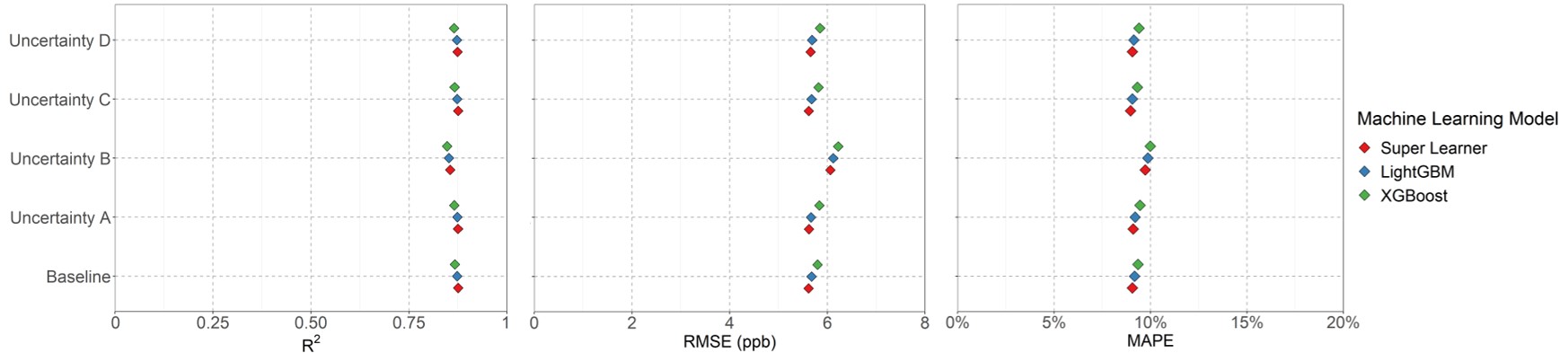 |
| Fall | 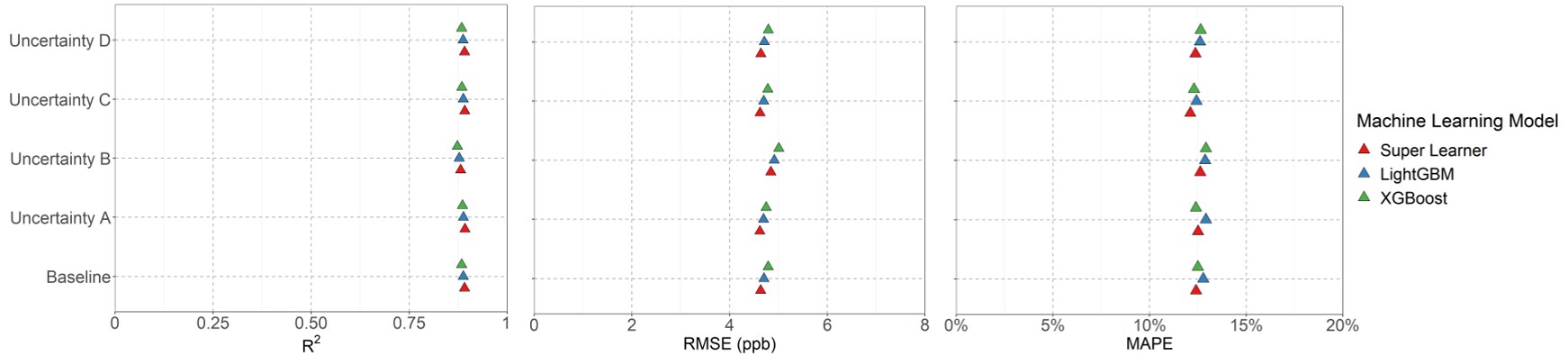 |
| Winter | 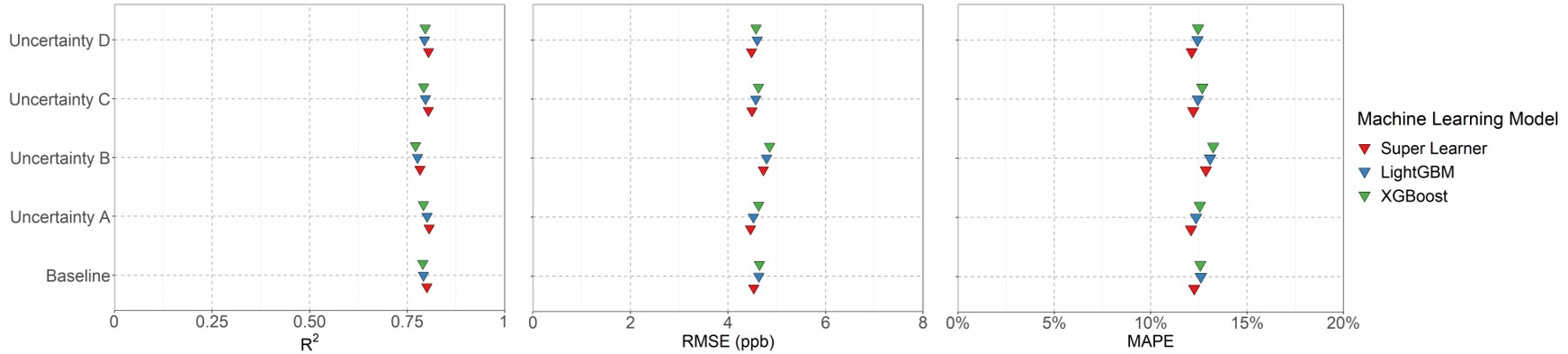 |
|  | 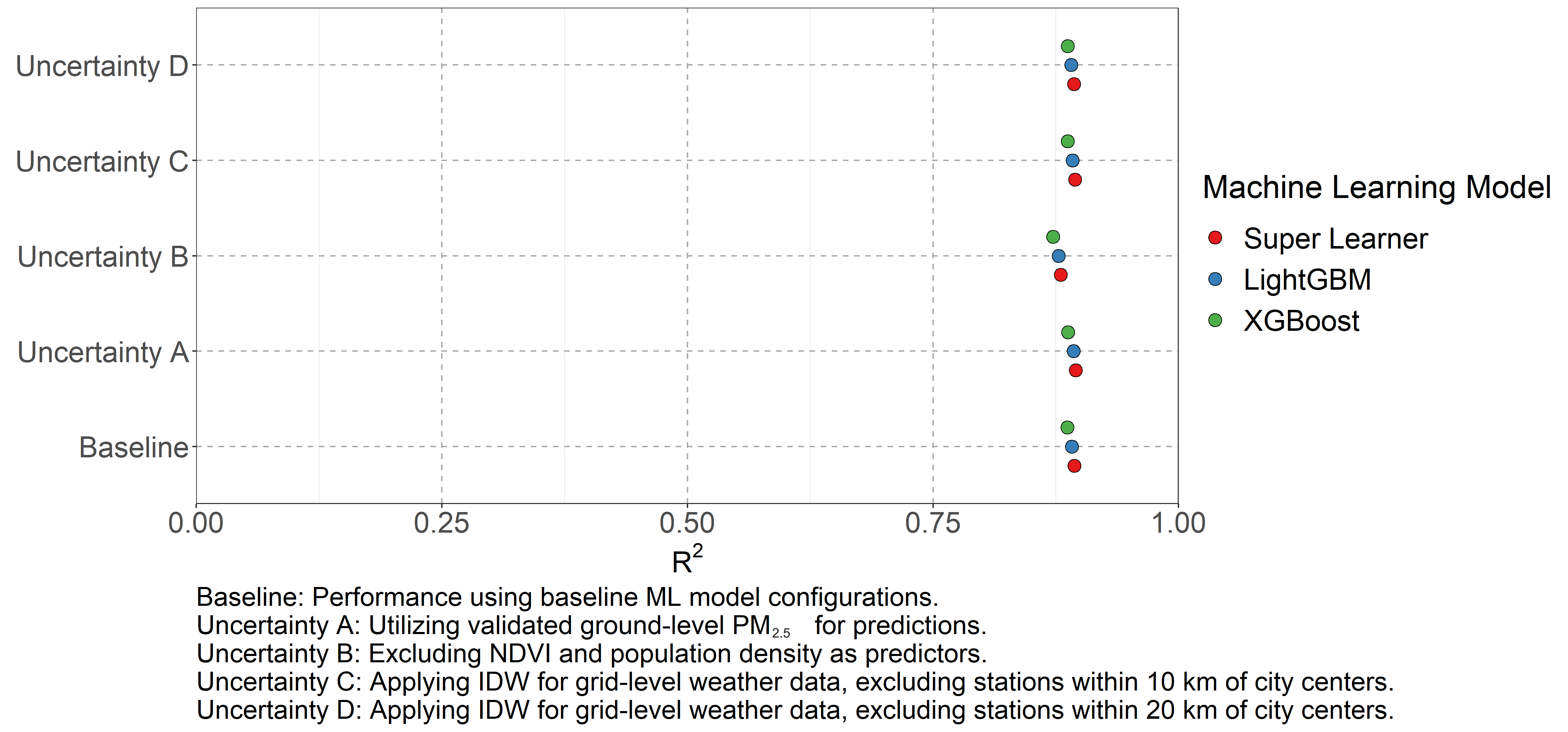 |

**Fig. S12.** Uncertainty ranges of cross-validation performances of machine learning models. These figures show model performance across seasons using the baseline model configurations and four alternative configurations.


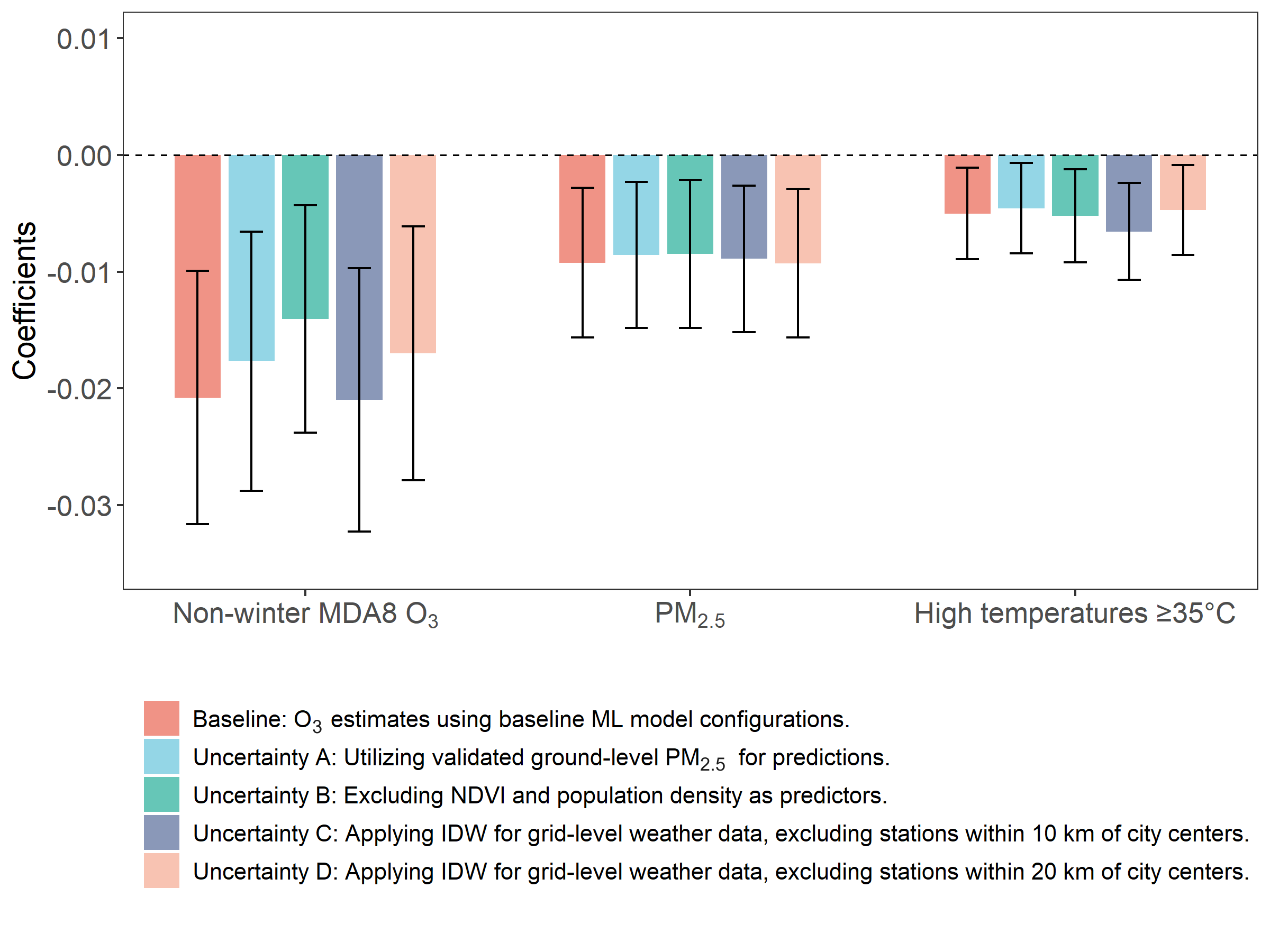


**Fig. S13.** Uncertainty ranges of estimated coefficients of the pollution and high temperature variables. Using the O_3_ estimates generated from the four uncertainty scenarios, we reconstructed the average MDA8 O_3_ concentrations for both winter and non-winter seasons. We then estimated Eqs. (1) and (2), with the dependent variables being the natural log of agricultural TFP derived from the TL-CPF model. The length of each bar shows the coefficients of the non-winter MDA8 O_3_ variable, the PM_2.5_ variable and the variable for high temperatures above 35°C. The whiskers are the corresponding 95% confidence intervals for these estimates. The black, dashed, horizontal line marks 0.

**Table S1. Sources of ground O_3_ observations used for historical validation in 2002-2012**

| Source | Region | Time period | # of sites | # of monthly observations |
| --- | --- | --- | --- | --- |
| Xu et al. (2) | Mainland China | 2002-2012 | 8 | 622 |
| Hong Kong Environmental Protection Department | Hong Kong | 2002-2012 | 11 | 1,452 |
| Taiwan Environmental Protection Agency | Taiwan | 2002-2012 | 76 | 9,887 |
| Macao Environmental Protection Agency | Macao | 2011-2012 | 5 | 120 |

| Regions | Sample size | LightGBM | | | XGBoost | | | Super Learner | | |
| --- | --- | --- | --- | --- | --- | --- | --- | --- | --- | --- |
|  |  | R^2^ | RMSE | MAPE | R^2^ | RMSE | MAPE | R^2^ | RMSE | MAPE |
| Overall (100) | 3,405 | 0.60 | 8.85 | 16.85% | 0.60 | 8.90 | 16.74% | 0.60 | 8.85 | 16.58% |
| North (3) | 219 | 0.77 | 11.35 | 24.27% | 0.76 | 11.54 | 23.96% | 0.77 | 11.31 | 23.92% |
| Northeast (1) | 86 | 0.55 | 7.03 | 13.87% | 0.53 | 7.19 | 13.92% | 0.55 | 7.04 | 13.77% |
| East (77) | 2,572 | 0.70 | 6.56 | 11.26% | 0.71 | 6.49 | 11.01% | 0.70 | 6.55 | 11.10% |
| PRD (16) | 288 | 0.49 | 6.70 | 20.63% | 0.58 | 6.08 | 19.00% | 0.55 | 6.26 | 19.50% |
| Qinghai-Tibet (2) | 202 | 0.67 | 6.78 | 11.65% | 0.61 | 7.40 | 12.41% | 0.65 | 6.96 | 11.88% |
| Northwest (1) | 38 | 0.74 | 4.88 | 8.97% | 0.76 | 4.71 | 8.70% | 0.76 | 4.71 | 8.70% |

**Table S2. Model performance in** **six subregions of China from 2002 to 2012 at the monthly level**

*Notes:* The first row displays the three machine-learning algorithms employed. RMSE stands for root-mean-squared prediction error. MAPE stands for mean absolute percentage prediction error. The numbers in parentheses in the first column are the number of ozone observation sites in each region.

**Table S3. Summary statistics**

| Variables | Unit | Obs | Mean | SD |
| --- | --- | --- | --- | --- |
| *Panel A: Annual agricultural data* |  |  |  |  |
| Total output value | billion CNY | 26,788 | 0.45 | 0.44 |
| Labor | thousand persons | 26,788 | 130.94 | 107.84 |
| Land | thousand ha | 26,788 | 45.85 | 44.83 |
| Machinery | thousand kw | 26,788 | 362.01 | 422.42 |
| Fertilizer | thousand tons | 26,788 | 26.35 | 40.67 |
| Labor productivity | CNY/person | 26,788 | 5,002.42 | 22,537.87 |
| Maize yield | ton/ha | 20,892 | 5.37 | 3.76 |
| Soybean yield | ton/ha | 21,833 | 2.27 | 2.74 |
| Rice yield | ton/ha | 9,291 | 6.95 | 2.22 |
| Wheat yield | ton/ha | 16,095 | 4.13 | 5.75 |
| Tuberous root yield | ton/ha | 22,868 | 4.80 | 7.58 |
| Milk per cow | ton/head | 18,683 | 3.50 | 7.45 |
|  |  |  |  |  |
| *Panel B: Annual Air pollution data* |  |  |  |  |
| Annual mean MDA8 O_3_ | ppb | 26,788 | 42.89 | 5.83 |
| Spring mean MDA8 O_3_ | ppb | 26,788 | 48.77 | 7.88 |
| Summer mean MDA8 O_3_ | ppb | 26,788 | 53.10 | 12.28 |
| Fall mean MDA8 O_3_ | ppb | 26,788 | 41.17 | 7.82 |
| Winter mean MDA8 O_3_ | ppb | 26,788 | 28.51 | 4.98 |
| Non-winter mean MDA8 O_3_ | ppb | 26,788 | 47.68 | 7.50 |
| Annual AOT40 | ppm h | 26,788 | 22.10 | 14.36 |
| Annual SUM06 | ppm h | 26,788 | 25.41 | 29.76 |
| Annual W126 | ppm h | 26,788 | 23.27 | 20.09 |
| Winter AOT40 | ppm h | 26,788 | 0.22 | 0.63 |
| Winter SUM06 | ppm h | 26,788 | 0.01 | 0.25 |
| Winter W126 | ppm h | 26,788 | 0.39 | 0.46 |
| Non-winter AOT40 | ppm h | 26,788 | 21.89 | 14.38 |
| Non-winter SUM06 | ppm h | 26,788 | 25.41 | 29.74 |
| Non-winter W126 | ppm h | 26,788 | 22.88 | 20.11 |
| Annual mean PM_2.5_ | μg/m^3^ | 26,788 | 55.54 | 26.12 |
|  |  |  |  |  |
| *Panel C: Annual weather data* |  |  |  |  |
| 0-5℃ | days | 26,788 | 32.73 | 15.71 |
| 5-10℃ | days | 26,788 | 43.42 | 12.96 |
| 10-15℃ | days | 26,788 | 49.52 | 11.26 |
| 15-20℃ | days | 26,788 | 59.97 | 14.25 |
| 20-25℃ | days | 26,788 | 67.41 | 17.63 |
| 25-30℃ | days | 26,788 | 51.23 | 23.48 |
| 30-35℃ | days | 26,788 | 19.04 | 14.16 |
| ≥35℃ | days | 26,788 | 1.88 | 2.72 |
| Average air pressure | hPa | 26,788 | 961.56 | 57.14 |
| Average wind speed | m/s | 26,788 | 2.07 | 0.62 |
| Average relative humidity | % | 26,788 | 68.63 | 8.83 |
| Total precipitation | cm | 26,788 | 94.40 | 47.87 |
| Total sunshine duration | hours | 26,788 | 1,966.59 | 504.13 |

*Notes:* This table shows summary statistics on our key variables of interest over the period 2002-2015 covering all available counties except for the Northwestern and Tibetan counties. Total output value is in 1980’s constant price. Unit of observation is a county-by-year pair.

**Table S4. The effects of pollution and temperature extremes on agricultural productivity: Extended panel A of Table 1**

| Dependent variable | Log (Agricultural productivity) | | | | | |
| --- | --- | --- | --- | --- | --- | --- |
|  | (1) | (2) | (3) | (4) | (5) | (6) |
|  | OLS | IV | IV | IV | IV | IV |
|  | TL-CPF | TL-CPF | TL-CPF-w/CRS | CD-CPF | CD-SFA-w/CRS | Labor productivity |
| Annual MDA8 O_3_ | -0.0008 | -0.0224^***^ | -0.0219^***^ | -0.0199^***^ | -0.0210^***^ | -0.0250^***^ |
|  | (0.0027) | (0.0070) | (0.0070) | (0.0069) | (0.0069) | (0.0084) |
| PM_2.5_ | -0.0039^***^ | -0.0092^***^ | -0.0102^***^ | -0.0087^***^ | -0.0087^***^ | -0.0058 |
|  | (0.0008) | (0.0033) | (0.0032) | (0.0031) | (0.0030) | (0.0038) |
| 0-5°C | 0.0002 | -0.0003 | -0.0005 | -0.0003 | 0.0002 | -0.0002 |
|  | (0.0005) | (0.0005) | (0.0005) | (0.0005) | (0.0005) | (0.0007) |
| 5-10°C | -0.0003 | -0.0004 | -0.0004 | -0.0003 | 0.0000 | -0.0002 |
|  | (0.0004) | (0.0004) | (0.0004) | (0.0004) | (0.0004) | (0.0006) |
| 10-15°C | -0.0002 | -0.0023^***^ | -0.0025^***^ | -0.0020^**^ | -0.0015^*^ | -0.0021^*^ |
|  | (0.0006) | (0.0009) | (0.0009) | (0.0009) | (0.0009) | (0.0011) |
| 15-20°C | -0.0018^**^ | -0.0023^***^ | -0.0027^***^ | -0.0026^***^ | -0.0023^***^ | -0.0020^*^ |
|  | (0.0007) | (0.0008) | (0.0008) | (0.0008) | (0.0008) | (0.0010) |
| 20-25°C | 0.0003 | 0.0002 | -0.0002 | -0.0001 | 0.0002 | 0.0009 |
|  | (0.0007) | (0.0007) | (0.0007) | (0.0007) | (0.0007) | (0.0009) |
| 25-30°C | -0.0011 | -0.0013 | -0.0015^*^ | -0.0011 | -0.0011 | 0.0015 |
|  | (0.0008) | (0.0009) | (0.0009) | (0.0008) | (0.0008) | (0.0011) |
| 30-35°C | 0.0003 | 0.0018 | 0.0014 | 0.0013 | 0.0013 | 0.0010 |
|  | (0.0013) | (0.0014) | (0.0014) | (0.0014) | (0.0014) | (0.0018) |
| ≥35°C | -0.0032^*^ | -0.0050^**^ | -0.0053^***^ | -0.0053^***^ | -0.0055^***^ | -0.0054^**^ |
|  | (0.0018) | (0.0020) | (0.0020) | (0.0020) | (0.0020) | (0.0026) |
| Precipitation | -0.0004 | -0.0007 | -0.0008 | -0.0005 | -0.0004 | -0.0008 |
|  | (0.0004) | (0.0005) | (0.0005) | (0.0005) | (0.0005) | (0.0006) |
| Precipitation Squared | 0.0000^*^ | 0.0000 | 0.0000 | 0.0000 | 0.0000 | 0.0000 |
|  | (0.0000) | (0.0000) | (0.0000) | (0.0000) | (0.0000) | (0.0000) |
| Sunshine Duration | 0.0001 | 0.0004^***^ | 0.0006^***^ | 0.0006^***^ | 0.0007^***^ | 0.0005^**^ |
|  | (0.0001) | (0.0002) | (0.0002) | (0.0002) | (0.0002) | (0.0002) |
| Sunshine Duration Squared | -0.0000 | -0.0000^**^ | -0.0000^***^ | -0.0000^***^ | -0.0000^***^ | -0.0000^**^ |
|  | (0.0000) | (0.0000) | (0.0000) | (0.0000) | (0.0000) | (0.0000) |
| Humidity | 0.0161 | 0.0209 | 0.0167 | 0.0112 | 0.0053 | 0.0175 |
|  | (0.0121) | (0.0134) | (0.0134) | (0.0129) | (0.0130) | (0.0152) |
| Humidity Squared | -0.0001 | -0.0002^**^ | -0.0002^*^ | -0.0001 | -0.0001 | -0.0002^*^ |
|  | (0.0001) | (0.0001) | (0.0001) | (0.0001) | (0.0001) | (0.0001) |
| Atmospheric Pressure | -0.0338 | -0.0287 | -0.0325 | -0.0214 | -0.0231 | -0.0311 |
|  | (0.0217) | (0.0219) | (0.0225) | (0.0208) | (0.0215) | (0.0252) |
| Atmospheric Pressure Squared | 0.0000 | 0.0000 | 0.0000 | 0.0000 | 0.0000 | 0.0000 |
|  | (0.0000) | (0.0000) | (0.0000) | (0.0000) | (0.0000) | (0.0000) |
| Wind Speed | 0.0284 | -0.0597 | -0.0922 | -0.0975 | -0.1255^*^ | -0.0989 |
|  | (0.0656) | (0.0693) | (0.0684) | (0.0671) | (0.0658) | (0.0751) |
| Wind Speed Squared | -0.0226 | -0.0053 | -0.0022 | 0.0002 | 0.0087 | 0.0074 |
|  | (0.0141) | (0.0143) | (0.0140) | (0.0138) | (0.0134) | (0.0151) |
| F-test (KP statistics) | - | 12.4088 | 12.4088 | 12.4088 | 12.4088 | 12.4088 |
| Observations | 26,788 | 26,788 | 26,788 | 26,788 | 26,788 | 26,788 |

*Notes:* This table shows estimated coefficients of pollution and weather on agricultural productivity. The dependent variables are the natural log of agricultural TFP derived from the TL-CPF model (columns 1-2), the TL-CPF-w/CRS model (column 3), the CD-CPF model (column 4), the CD-SFA-w/CRS model (column 5), and labor productivity (defined as the output per agricultural worker, column 6). Column 1 reports the OLS estimates. Columns 2-6 report the estimated coefficients from the instrumental variable design. All regressions include the number of days with daily temperatures falling into specific bins at a width of 5°C, as well as linear and quadratic terms of cumulative precipitation, sunshine duration, average relative humidity, air pressure and wind speed as weather controls. The symbol “$\geq$35°C” denotes the number of days with daily temperatures exceeding 35°C. All regressions include county fixed effects and year fixed effects. Standard errors (in parentheses) are clustered at county level. Significance: *** p<0.01, ** p<0.05, * p<0.1.

**Table S5. The effects of pollution and temperature extremes on agricultural productivity using cumulative O_3_ indicators (W126, AOT40, and SUM06)**

|  | (1) | (2) | (3) |
| --- | --- | --- | --- |
| Panel A: Productivity responses to annual O_3_ indicators | | | |
| Annual O_3_ measure | -0.0036^***^ | -0.0091^***^ | -0.0022^**^ |
|  | (0.0014) | (0.0024) | (0.0009) |
| PM_2.5_ | -0.0094^***^ | -0.0099^***^ | -0.0090^***^ |
|  | (0.0033) | (0.0033) | (0.0033) |
| ≥35°C | -0.0053^**^ | -0.0058^***^ | -0.0054^**^ |
|  | (0.0021) | (0.0020) | (0.0022) |
| F-test (KP statistics) | 12.4409 | 13.2766 | 10.7058 |
| Observations | 26,788 | 26,788 | 26,788 |
| Panel B: Productivity responses to non-winter O_3_ indicators | | | |
| Non-winter O_3_ measure | -0.0036^***^ | -0.0089^***^ | -0.0022^**^ |
|  | (0.0014) | (0.0024) | (0.0009) |
| PM_2.5_ | -0.0094^***^ | -0.0100^***^ | -0.0090^***^ |
|  | (0.0033) | (0.0032) | (0.0033) |
| ≥35°C | -0.0052^**^ | -0.0057^***^ | -0.0054^**^ |
|  | (0.0021) | (0.0020) | (0.0022) |
| F-test (KP statistics) | 12.5077 | 13.4449 | 10.7119 |
| Observations | 26,788 | 26,788 | 26,788 |

*Notes:* This table presents the IV estimates of O_3_, PM_2.5_ pollution, and high temperatures above 35°C on agricultural TFP. We used the TL-CPF model for TFP calculations and an instrumental variable approach for these estimates. In columns 1-3, we used W126, AOT40, and SUM06, respectively, as O_3_ measures. In panel A, these measures were computed over the entire year. In panel B, they were computed during the non-winter seasons. All regressions include the number of days with daily temperatures falling into specific bins at a width of 5°C, as well as linear and quadratic terms of cumulative precipitation, sunshine duration, average relative humidity, air pressure and wind speed as weather controls. The symbol “$\geq$35°C” denotes the number of days with daily temperatures exceeding 35°C. All regressions include county fixed effects and year fixed effects. Standard errors (in parentheses) are clustered at county level. Significance: *** p<0.01, ** p<0.05, * p<0.1.

**Table S6. The effect of pollution and temperature extremes on agricultural productivity: Extended panel B of Table 1**

| Dependent variable | Log (Agricultural productivity) | | | | | |
| --- | --- | --- | --- | --- | --- | --- |
|  | (1) | (2) | (3) | (4) | (5) | (6) |
|  | OLS | IV | IV | IV | IV | IV |
|  | TL-CPF | TL-CPF | TL-CPF-w/CRS | CD-CPF | CD-SFA-w/CRS | Labor productivity |
| Winter MDA8 O_3_ | -0.0027^*^ | 0.0051 | 0.0016 | 0.0028 | 0.0043 | 0.0127 |
|  | (0.0014) | (0.0088) | (0.0087) | (0.0086) | (0.0087) | (0.0103) |
| Non winter MDA8 O_3_ | 0.0005 | -0.0208^***^ | -0.0191^***^ | -0.0178^***^ | -0.0194^***^ | -0.0259^***^ |
|  | (0.0023) | (0.0055) | (0.0055) | (0.0054) | (0.0053) | (0.0068) |
| PM_2.5_ | -0.0039^***^ | -0.0092^***^ | -0.0102^***^ | -0.0087^***^ | -0.0087^***^ | -0.0060 |
|  | (0.0008) | (0.0033) | (0.0032) | (0.0031) | (0.0030) | (0.0038) |
| 0-5°C | 0.0002 | -0.0001 | -0.0004 | -0.0002 | 0.0003 | 0.0001 |
|  | (0.0005) | (0.0006) | (0.0006) | (0.0006) | (0.0006) | (0.0007) |
| 5-10°C | -0.0004 | -0.0000 | -0.0002 | -0.0001 | 0.0003 | 0.0004 |
|  | (0.0004) | (0.0005) | (0.0005) | (0.0005) | (0.0005) | (0.0007) |
| 10-15°C | -0.0002 | -0.0023^***^ | -0.0025^***^ | -0.0021^**^ | -0.0016^*^ | -0.0022^**^ |
|  | (0.0006) | (0.0009) | (0.0009) | (0.0008) | (0.0008) | (0.0011) |
| 15-20°C | -0.0017^**^ | -0.0028^***^ | -0.0029^***^ | -0.0029^***^ | -0.0027^***^ | -0.0027^**^ |
|  | (0.0007) | (0.0009) | (0.0009) | (0.0009) | (0.0008) | (0.0011) |
| 20-25°C | 0.0004 | -0.0004 | -0.0006 | -0.0005 | -0.0003 | -0.0001 |
|  | (0.0007) | (0.0008) | (0.0008) | (0.0008) | (0.0008) | (0.0010) |
| 25-30°C | -0.0010 | -0.0015^*^ | -0.0017^**^ | -0.0013 | -0.0013 | 0.0010 |
|  | (0.0008) | (0.0009) | (0.0009) | (0.0008) | (0.0008) | (0.0011) |
| 30-35°C | 0.0003 | 0.0018 | 0.0014 | 0.0013 | 0.0013 | 0.0009 |
|  | (0.0013) | (0.0014) | (0.0014) | (0.0014) | (0.0014) | (0.0018) |
| ≥35°C | -0.0032^*^ | -0.0050^**^ | -0.0054^***^ | -0.0054^***^ | -0.0056^***^ | -0.0055^**^ |
|  | (0.0018) | (0.0020) | (0.0020) | (0.0020) | (0.0019) | (0.0026) |
| Precipitation | -0.0004 | -0.0007 | -0.0007 | -0.0004 | -0.0003 | -0.0008 |
|  | (0.0004) | (0.0005) | (0.0005) | (0.0005) | (0.0005) | (0.0006) |
| Precipitation Squared | 0.0000^*^ | 0.0000 | 0.0000 | 0.0000 | 0.0000 | 0.0000 |
|  | (0.0000) | (0.0000) | (0.0000) | (0.0000) | (0.0000) | (0.0000) |
| Sunshine Duration | 0.0002 | 0.0004^**^ | 0.0005^***^ | 0.0006^***^ | 0.0007^***^ | 0.0004^**^ |
|  | (0.0001) | (0.0002) | (0.0002) | (0.0002) | (0.0002) | (0.0002) |
| Sunshine Duration Squared | -0.0000 | -0.0000^*^ | -0.0000^***^ | -0.0000^***^ | -0.0000^***^ | -0.0000^*^ |
|  | (0.0000) | (0.0000) | (0.0000) | (0.0000) | (0.0000) | (0.0000) |
| Humidity | 0.0171 | 0.0166 | 0.0139 | 0.0081 | 0.0014 | 0.0098 |
|  | (0.0122) | (0.0141) | (0.0140) | (0.0135) | (0.0136) | (0.0159) |
| Humidity Squared | -0.0001 | -0.0002^*^ | -0.0002 | -0.0001 | -0.0001 | -0.0001 |
|  | (0.0001) | (0.0001) | (0.0001) | (0.0001) | (0.0001) | (0.0001) |
| Atmospheric Pressure | -0.0353 | -0.0227 | -0.0285 | -0.0170 | -0.0177 | -0.0205 |
|  | (0.0217) | (0.0223) | (0.0229) | (0.0211) | (0.0218) | (0.0257) |
| Atmospheric Pressure Squared | 0.0000 | 0.0000 | 0.0000 | 0.0000 | 0.0000 | 0.0000 |
|  | (0.0000) | (0.0000) | (0.0000) | (0.0000) | (0.0000) | (0.0000) |
| Wind Speed | 0.0342 | -0.0813 | -0.1066 | -0.1133^*^ | -0.1449^**^ | -0.1373^*^ |
|  | (0.0656) | (0.0702) | (0.0693) | (0.0677) | (0.0663) | (0.0755) |
| Wind Speed Squared | -0.0233^*^ | -0.0029 | -0.0006 | 0.0020 | 0.0109 | 0.0117 |
|  | (0.0141) | (0.0143) | (0.0140) | (0.0137) | (0.0134) | (0.0150) |
| F-test (KP statistics) | - | 10.9139 | 10.9139 | 10.9139 | 10.9139 | 10.9139 |
| Observations | 26,788 | 26,788 | 26,788 | 26,788 | 26,788 | 26,788 |

*Notes:* This table shows estimated coefficients of pollution and weather on agricultural productivity. The dependent variables are the natural log of agricultural TFP derived from the TL-CPF model (columns 1-2), the TL-CPF-w/CRS model (column 3), the CD-CPF model (column 4), the CD-SFA-w/CRS model (column 5), and labor productivity (defined as the output per agricultural worker, column 6). Column 1 reports the OLS estimates. Columns 2-6 report the estimated coefficients from the instrumental variable design. All regressions include the number of days with daily temperatures falling into specific bins at a width of 5°C, as well as linear and quadratic terms of cumulative precipitation, sunshine duration, average relative humidity, air pressure and wind speed as weather controls. The symbol “$\geq$35°C” denotes the number of days with daily temperatures exceeding 35°C. All regressions include county fixed effects and year fixed effects. Standard errors (in parentheses) are clustered at county level. Significance: *** p<0.01, ** p<0.05, * p<0.1.

**Table S7. Robustness of main estimates to alternative clustering choices**

| Dependent variable | Log (Agricultural TFP) | | |
| --- | --- | --- | --- |
|  | (1) | (2) | (3) |
| Winter MDA8 O_3_ | 0.0051 | 0.0051 | 0.0051 |
|  | (0.0088) | (0.0101) | (0.0100) |
| Non-winter MDA8 O_3_ | -0.0208^***^ | -0.0208^***^ | -0.0208^***^ |
|  | (0.0055) | (0.0066) | (0.0069) |
| PM_2.5_ | -0.0092^***^ | -0.0092^**^ | -0.0092^**^ |
|  | (0.0033) | (0.0035) | (0.0039) |
| ≥35°C | -0.0050^**^ | -0.0050 | -0.0050 |
|  | (0.0020) | (0.0037) | (0.0037) |
| Clustering variables | County | County and year | County and  region-year |
| F-test (KP statistics) | 10.9139 | 25.5930 | 8.2472 |
| Observations | 26,788 | 26,788 | 26,788 |

*Notes:* This table shows IV estimates of Eq. (1) from the main text. Standard errors (in parentheses) are clustered by variables indicated in the columns. Dependent variable is the natural log of agricultural TFP derived from the TL-CPF model. All regressions include the number of days with daily temperatures falling into specific bins at a width of 5°C, as well as linear and quadratic terms of cumulative precipitation, sunshine duration, average relative humidity, air pressure and wind speed as weather controls. The symbol “$\geq$35°C” denotes the number of days with daily temperatures exceeding 35°C. Significance: *** p<0.01, ** p<0.05, * p<0.1.

**Table S8. Robustness of main estimates to different fixed effects, time trends, and weather controls**

| Dependent variable | Log (Agricultural TFP) | | | | | |
| --- | --- | --- | --- | --- | --- | --- |
|  | (1) | (2) | (3) | (4) | (5) | (6) |
| Winter MDA8 O_3_ | 0.0068 | 0.0051 | -0.0012 | 0.0098 | 0.0098 | 0.0049 |
|  | (0.0064) | (0.0088) | (0.0091) | (0.0083) | (0.0081) | (0.0086) |
| Non-winter MDA8 O_3_ | -0.0096^**^ | -0.0208^***^ | -0.0312^***^ | -0.0343^***^ | -0.0219^***^ | -0.0208^***^ |
|  | (0.0044) | (0.0055) | (0.0080) | (0.0083) | (0.0054) | (0.0056) |
| PM_2.5_ | -0.0090^***^ | -0.0092^***^ | -0.0074^**^ | -0.0041 | -0.0102^***^ | -0.0100^***^ |
|  | (0.0031) | (0.0033) | (0.0033) | (0.0033) | (0.0033) | (0.0032) |
| ≥35°C |  | -0.0050^**^ | -0.0022 | -0.0003 | -0.0035^*^ | -0.0008 |
|  |  | (0.0020) | (0.0018) | (0.0015) | (0.0018) | (0.0013) |
| Type of weather control | None | Full | Full | Full | Linear | GDD |
| County FE | X | X | X | X | X | X |
| Year FE | X | X | X | X | X | X |
| Region time trend |  |  | X |  |  |  |
| Province time trend |  |  |  | X |  |  |
| F-test (KP statistics) | 11.3905 | 10.9139 | 10.3946 | 9.6288 | 12.0211 | 10.9328 |
| Observations | 26,788 | 26,788 | 26,788 | 26,788 | 26,788 | 26,788 |

*Notes:* This table shows IV estimates of Eq. (1) from the main text with different time trends and weather controls. Dependent variable is the natural log of agricultural TFP derived from the TL-CPF model. Column 1 excludes weather controls. Column 2 repeats the baseline results. Columns 3 and 4 use the same weather controls as in our baseline model, with varying time trends. Column 5 removes the quadratic terms of weather controls. Column 6 uses growing degree days (GDD), which is defined as the sum of heat received between two consecutive 5°C temperature bins, as the temperature variables. Standard errors (in parentheses) are clustered at county level. Significance: *** p<0.01, ** p<0.05, * p<0.1.

**Table S9. Robustness of main estimates to instrument choices**

| Dependent variable | Log (Agricultural TFP) | | | | |
| --- | --- | --- | --- | --- | --- |
|  | (1) | (2) | (3) | (4) | (5) |
| Winter MDA8 O_3_ | 0.0051 | 0.0061 | -0.0023 | -0.0013 | 0.0106 |
|  | (0.0088) | (0.0073) | (0.0078) | (0.0069) | (0.0157) |
| Non-winter MDA8 O_3_ | -0.0208^***^ | -0.0173^***^ | -0.0123^***^ | -0.0088^*^ | -0.0313^***^ |
|  | (0.0055) | (0.0053) | (0.0047) | (0.0046) | (0.0080) |
| PM_2.5_ | -0.0092^***^ | -0.0075^***^ | -0.0094^***^ | -0.0076^***^ | -0.0127^**^ |
|  | (0.0033) | (0.0027) | (0.0029) | (0.0026) | (0.0050) |
| ≥35°C | -0.0050^**^ | -0.0048^**^ | -0.0041^**^ | -0.0038^**^ | -0.0057^***^ |
|  | (0.0020) | (0.0019) | (0.0019) | (0.0019) | (0.0022) |
| Size of wind angle bins | 90 | 90 | 60 | 60 | 90 |
| Number of county groups | 50 | 75 | 50 | 75 | 50 |
| F-test (KP statistics) | 10.9139 | 10.7257 | 8.6448 | 16.5668 | 10.9139 |
| Observations | 26,788 | 26,788 | 26,788 | 26,788 | 26,788 |

*Notes:* Columns 1-4 shows IV estimates of Eq. (1) from the main text when varying with the size of wind angle bins and the number of county groups. Dependent variable is the natural log of agricultural TFP derived from the TL-CPF model, the same as in the baseline model. Column 1 reports the baseline estimate. Column 5 reports the estimate when using the LIML estimator instead of the 2SLS estimator. All regressions include the number of days with daily temperatures falling into specific bins at a width of 5°C, as well as linear and quadratic terms of cumulative precipitation, sunshine duration, average relative humidity, air pressure and wind speed as weather controls. The symbol “$\geq$35°C” denotes the number of days with daily temperatures exceeding 35°C. Significance: *** p<0.01, ** p<0.05, * p<0.1.

**Table S10. Robustness of main estimates to data treatment**

| Dependent variable | Log (Agricultural TFP) | |
| --- | --- | --- |
|  | (1) | (2) |
| Winter MDA8 O_3_ | 0.0062 | 0.0099 |
|  | (0.0084) | (0.0080) |
| Non-winter MDA8 O_3_ | -0.0187^***^ | -0.0164^***^ |
|  | (0.0052) | (0.0049) |
| PM_2.5_ | -0.0080^**^ | -0.0065^**^ |
|  | (0.0031) | (0.0030) |
| ≥35°C | -0.0050^***^ | -0.0048^***^ |
|  | (0.0018) | (0.0018) |
| F-test (KP statistics) | 10.9139 | 10.8402 |
| Observations | 26,788 | 26,518 |

*Notes:* This table shows IV estimates of Eq. (1) from the main text with data treatment. Column 1 reports the results when replacing the TFP estimates that are larger (or smaller) than the 99.5th (or the 0.5th) percentile by the 99.5th (or the 0.5th) percentile estimates. Column 2 reports the results after removing observations when the TFP estimates are larger (or smaller) than the 99.5th (or the 0.5th) percentile. Dependent variable is the natural log of agricultural TFP derived from the TL-CPF model, the same as in the baseline model. All regressions include the number of days with daily temperatures falling into specific bins at a width of 5°C, as well as linear and quadratic terms of cumulative precipitation, sunshine duration, average relative humidity, air pressure and wind speed as weather controls. The symbol “$\geq$35°C” denotes the number of days with daily temperatures exceeding 35°C. All regressions include county fixed effects and year fixed effects. Standard errors (in parentheses) are clustered at county level. Significance: *** p<0.01, ** p<0.05, * p<0.1.

**Table S11. Robustness of main estimates to the exclusion of coastal counties**

| Dependent variable | Log (Agricultural TFP) | | | | | |  |
| --- | --- | --- | --- | --- | --- | --- | --- |
|  | (1) | (2) | (3) | (4) | (5) | (6) |  |
|  | OLS | IV | IV | IV | IV | IV |  |
|  | TL-CPF | TL-CPF | TL-CPF-w/CRS | CD-CPF | CD-SFA-w/CRS | Labor productivity |  |
| Panel A: Productivity responses to annual mean MDA8 O_3_ | | | | | | | |
| Annual MDA8 O_3_ | 0.0016 | -0.0195^***^ | -0.0198^***^ | -0.0174^***^ | -0.0188^***^ | -0.0237^***^ |  |
|  | (0.0028) | (0.0066) | (0.0066) | (0.0065) | (0.0064) | (0.0082) |  |
| PM_2.5_ | -0.0033^***^ | -0.0099^***^ | -0.0110^***^ | -0.0095^***^ | -0.0096^***^ | -0.0087^**^ |  |
|  | (0.0008) | (0.0031) | (0.0030) | (0.0029) | (0.0029) | (0.0038) |  |
| ≥35°C | -0.0030 | -0.0046^**^ | -0.0051^**^ | -0.0050^**^ | -0.0053^***^ | -0.0050^*^ |  |
|  | (0.0019) | (0.0021) | (0.0021) | (0.0021) | (0.0021) | (0.0028) |  |
| F-test (KP statistics) | - | 13.1388 | 13.1388 | 13.1388 | 13.1388 | 13.1388 |  |
| Observations | 24,821 | 24,821 | 24,821 | 24,821 | 24,821 | 24,821 |  |
| Panel B: Productivity responses to winter and non-winter MDA8 O_3_ | | | | | | | |
| Winter MDA8 O_3_ | -0.0025^*^ | -0.0026 | -0.0065 | -0.0048 | -0.0025 | 0.0029 |  |
|  | (0.0015) | (0.0084) | (0.0083) | (0.0082) | (0.0083) | (0.0104) |  |
| Non-winter MDA8 O_3_ | 0.0024 | -0.0155^***^ | -0.0142^***^ | -0.0129^**^ | -0.0149^***^ | -0.0212^***^ |  |
|  | (0.0024) | (0.0052) | (0.0052) | (0.0050) | (0.0050) | (0.0066) |  |
| PM_2.5_ | -0.0033^***^ | -0.0100^***^ | -0.0110^***^ | -0.0095^***^ | -0.0096^***^ | -0.0088^**^ |  |
|  | (0.0008) | (0.0031) | (0.0030) | (0.0029) | (0.0029) | (0.0038) |  |
| ≥35°C | -0.0029 | -0.0047^**^ | -0.0050^**^ | -0.0049^**^ | -0.0054^***^ | -0.0051^*^ |  |
|  | (0.0019) | (0.0021) | (0.0021) | (0.0021) | (0.0021) | (0.0027) |  |
| F-test (KP statistics) | - | 10.9573 | 10.9573 | 10.9573 | 10.9573 | 10.9573 |  |
| Observations | 24,821 | 24,821 | 24,821 | 24,821 | 24,821 | 24,821 |  |

*Notes:* This table shows estimated coefficients of pollution and high temperatures on agricultural productivity for a subsample that excludes observations from 227 coastal counties. The dependent variables are the natural log of agricultural TFP derived from the TL-CPF model (columns 1-2), the TL-CPF-w/CRS model (column 3), the CD-CPF model (column 4), the CD-SFA-w/CRS model (column 5), and labor productivity (defined as the output per agricultural worker in column 6). Column 1 reports the OLS estimates. Columns 2-6 report the estimated coefficients from the instrumental variable design. All regressions include the number of days with daily temperatures falling into specific bins at a width of 5°C, as well as linear and quadratic terms of cumulative precipitation, sunshine duration, average relative humidity, air pressure and wind speed as weather controls. The symbol “$\geq$35°C” denotes the number of days with daily temperatures exceeding 35°C. All regressions include county fixed effects and year fixed effects. Standard errors (in parentheses) are clustered at county level. Significance: *** p<0.01, ** p<0.05, * p<0.1.

**Table S12. Robustness of main estimates to the exclusion of the PM_2.5_ variable**

| Dependent variable | Log (Agricultural TFP) | | | | | |
| --- | --- | --- | --- | --- | --- | --- |
|  | (1) | (2) | (3) | (4) | (5) | (6) |
|  | OLS | IV | IV | IV | IV | IV |
|  | TL-CPF | TL-CPF | TL-CPF-w/CRS | CD-CPF | CD-SFA-w/CRS | Labor productivity |
| Winter MDA8 O_3_ | -0.0029^**^ | 0.0056 | 0.0023 | 0.0034 | 0.0049 | 0.0131 |
|  | (0.0014) | (0.0088) | (0.0087) | (0.0087) | (0.0088) | (0.0103) |
| Non-winter MDA8 O_3_ | 0.0012 | -0.0176^***^ | -0.0156^***^ | -0.0148^***^ | -0.0164^***^ | -0.0239^***^ |
|  | (0.0023) | (0.0055) | (0.0054) | (0.0053) | (0.0052) | (0.0067) |
| ≥35°C | -0.0039^**^ | -0.0064^***^ | -0.0069^***^ | -0.0066^***^ | -0.0069^***^ | -0.0064^**^ |
|  | (0.0018) | (0.0020) | (0.0020) | (0.0019) | (0.0020) | (0.0026) |
| F-test (KP statistics) | - | 11.1781 | 11.1781 | 11.1781 | 11.1781 | 11.1781 |
| Observations | 26,788 | 26,788 | 26,788 | 26,788 | 26,788 | 26,788 |

*Notes:* This table shows estimated coefficients of O_3_ and high temperatures on agricultural productivity. The dependent variables are the natural log of agricultural TFP derived from the TL-CPF model (columns 1-2), the TL-CPF-w/CRS model (column 3), the CD-CPF model (column 4), the CD-SFA-w/CRS model (column 5), and labor productivity (defined as the output per agricultural worker in column 6). Column 1 reports the OLS estimates. Columns 2-6 report the estimated coefficients from the instrumental variable design. All regressions include the number of days with daily temperatures falling into specific bins at a width of 5°C, as well as linear and quadratic terms of cumulative precipitation, sunshine duration, average relative humidity, air pressure and wind speed as weather controls. The symbol “$\geq$35°C” denotes the number of days with daily temperatures exceeding 35°C. All regressions include county fixed effects and year fixed effects. Standard errors (in parentheses) are clustered at county level. Significance: *** p<0.01, ** p<0.05, * p<0.1.

**Table S13. Regional heterogeneity**

|  | (1)  Northeast and North China | (2)  Northwest | (3)  Southwest | (4)  South and Yangtze River | (5)  Major grain- producing regions | (6)  Major livestock- producing regions |
| --- | --- | --- | --- | --- | --- | --- |
| Panel A: Productivity responses to annual mean MDA8 O_3_ | | | | | | |
| Annual MDA8 O_3_ | -0.0288^**^ | -0.0101 | 0.0046 | -0.0267^**^ | -0.0139^*^ | -0.0165 |
|  | (0.0122) | (0.0208) | (0.0275) | (0.0126) | (0.0077) | (0.0101) |
| PM_2.5_ | -0.0088^*^ | -0.0040 | -0.0306^***^ | -0.0189^**^ | -0.0128^***^ | -0.0073 |
|  | (0.0053) | (0.0089) | (0.0112) | (0.0074) | (0.0034) | (0.0048) |
| ≥35°C | -0.0175^***^ | -0.0097 | -0.0050 | 0.0033 | 0.0003 | -0.0066^**^ |
|  | (0.0062) | (0.0110) | (0.0047) | (0.0035) | (0.0033) | (0.0033) |
| F-test (KP statistics) | 80.3205 | 242.8862 | 10.3461 | 22.1434 | 43.1182 | 507.7526 |
| Observations | 8,095 | 4,830 | 5,826 | 8,037 | 10,335 | 6,927 |
| Panel B: Productivity responses to non-winter mean MDA8 O_3_ | | | | | | |
| Non-winter MDA8 O_3_ | -0.0266^***^ | -0.0079 | -0.0021 | -0.0233^**^ | -0.01678^**^ | -0.0089 |
|  | (0.0102) | (0.0168) | (0.0236) | (0.0106) | (0.0069) | (0.0085) |
| PM_2.5_ | -0.0092^*^ | -0.0039 | -0.0301^***^ | -0.0195^**^ | -0.0122^***^ | -0.0076 |
|  | (0.0053) | (0.0089) | (0.0115) | (0.0076) | (0.0034) | (0.0048) |
| ≥35°C | -0.0176^***^ | -0.0095 | -0.0048 | 0.0045 | 0.0001 | -0.0062^**^ |
|  | (0.0061) | (0.0109) | (0.0046) | (0.0036) | (0.0033) | (0.0033) |
| F-test (KP statistics) | 109.1187 | 230.9991 | 9.2849 | 24.8898 | 37.085 | 152.425 |
| Observations | 8,095 | 4,830 | 5,826 | 8,037 | 10,335 | 6,927 |

*Notes:* This table presents estimated coefficients for the effects of O_3_ (measured using mean MDA8), PM_2.5_, and high temperatures on agricultural TFP across regions in China. We used the TL-CPF model for TFP calculations and an instrumental variable approach for the estimates. Following the guidelines in the “Sustainable Agricultural Development Planning” from China’s Ministry of Agriculture and Rural Affairs (MARA), we divided the sample counties into four agricultural divisions, including: (1) the Northeast and North China Region (Beijing, Tianjin, Hebei, Henan, Liaoning, Jilin, Heilongjiang, and Shandong), (2) the Northwest Region (Shanxi, Inner Mongolia, Shanxi, Gansu, and Ningxia), (3) the Southwest Region (Guangxi, Chongqing, Sichuan, Guizhou, and Yunnan), and (4) the South and Yangtze River Region (Shanghai, Zhejiang, Fujian, Jiangxi, Guangdong, Hainan, Hubei, Hunan, Jiangsu, and Anhui). In columns 5 and 6, the regressions were conducted using the list of regions designated as major grain- or livestock- producing regions by the MARA. All regressions include the number of days with daily temperatures falling into specific bins at a width of 5°C, as well as linear and quadratic terms of cumulative precipitation, sunshine duration, average relative humidity, air pressure and wind speed as weather controls. The symbol “$\geq$35°C” denotes the number of days with daily temperatures exceeding 35°C. All regressions include county fixed effects and year fixed effects. Standard errors (in parentheses) are clustered at county level. Significance: *** p<0.01, ** p<0.05, * p<0.1.

**Table S14. The effects of W126, PM_2.5_, and high temperatures on crop and livestock yields**

|  | (1) | (2) | (3) | (4) | (5) | (6) |
| --- | --- | --- | --- | --- | --- | --- |
|  | Maize  yield | Soybean  yield | Single-season rice  yield | Wheat  yield | Tuberous  yield | Milk  per Cow |
| Growing-season  O_3_ measure | -0.0051^***^ | 0.0015 | -0.0036^*^ | -0.0033^**^ | -0.0062^**^ | 0.0019 |
|  | (0.0010) | (0.0018) | (0.0018) | (0.0016) | (0.0025) | (0.0031) |
| PM_2.5_ | 0.0028 | -0.0045 | 0.0031 | -0.0052 | 0.0077 | -0.0078 |
|  | (0.0024) | (0.0034) | (0.0034) | (0.0038) | (0.0049) | (0.0068) |
| ≥35°C | 0.0004 | 0.0016 | -0.0092^***^ | -0.0108^***^ | -0.0072 | 0.0064 |
|  | (0.0025) | (0.0029) | (0.0026) | (0.0036) | (0.0045) | (0.0055) |
| F-test (KP statistics) | 11.8983 | 12.5399 | 27.9687 | 35.1795 | 8.5134 | 10.5098 |
| Observations | 20,892 | 21,833 | 9,291 | 16,095 | 22,868 | 18,683 |

*Notes:* This table presents IV estimates for the effects of O_3_ (measured using W126), PM_2.5_ pollution, and high temperatures during the growing seasons on crop and livestock yields. Columns 1-6 report the results using the natural logarithms of yields for five major crops and milk in China as the dependent variables. The growing seasons for each product are defined as follows: (1) Maize: March – October; (2) Soybeans: May – October; (3) Single-season rice: April – October; (4) Wheat: September (previous year)– August; (5) Tubers: March – November; (6) Milk: the entire year. All regressions include the number of days with daily temperatures falling into specific bins at a width of 5°C, as well as linear and quadratic terms of cumulative precipitation, sunshine duration, average relative humidity, air pressure and wind speed as weather controls. The symbol “$\geq$35°C” denotes the number of days with daily temperatures exceeding 35°C. All regressions include county fixed effects and year fixed effects. Standard errors (in parentheses) are clustered at county level. Significance: *** p<0.01, ** p<0.05, * p<0.1.

**Table S15: The effects of AOT40, PM_2.5_, and high temperatures on crop and livestock yields**

|  | (1) | (2) | (3) | (4) | (5) | (6) |
| --- | --- | --- | --- | --- | --- | --- |
|  | Maize  yield | Soybean  yield | Single-season rice  yield | Wheat  yield | Tuberous yield | Milk  per Cow |
| Growing-season  O_3_ measure | -0.0099^***^ | 0.0033 | -0.0070^**^ | -0.0061^**^ | -0.0082^*^ | 0.0021 |
|  | (0.0019) | (0.0033) | (0.0034) | (0.0030) | (0.0044) | (0.0057) |
| PM_2.5_ | 0.0027 | -0.0044 | 0.0030 | -0.0051 | 0.0075 | -0.0083 |
|  | (0.0024) | (0.0034) | (0.0034) | (0.0038) | (0.0049) | (0.0069) |
| ≥35°C | 0.0012 | 0.0016 | -0.0093^***^ | -0.0102^***^ | -0.0052 | 0.0057 |
|  | (0.0025) | (0.0029) | (0.0026) | (0.0036) | (0.0043) | (0.0053) |
| F-test (KP statistics) | 13.1443 | 13.3689 | 30.3991 | 37.2780 | 8.7585 | 11.7872 |
| Observations | 20,892 | 21,833 | 9,291 | 16,095 | 22,868 | 18,683 |

*Notes:* This table presents the IV estimates for the effects of O_3_ (measured using AOT40), PM_2.5_ pollution, and high temperatures during the growing seasons on crop and livestock yields. Columns 1-6 report the results using the natural logarithms of yields for five major crops and milk in China as the dependent variables. The growing seasons for each product are defined as follows: (1) Maize: March – October; (2) Soybeans: May – October; (3) Single-season rice: April – October; (4) Wheat: September (previous year)– August; (5) Tubers: March – November; (6) Milk: the entire year. All regressions include the number of days with daily temperatures falling into specific bins at a width of 5°C, as well as linear and quadratic terms of cumulative precipitation, sunshine duration, average relative humidity, air pressure and wind speed as weather controls. The symbol “$\geq$35°C” denotes the number of days with daily temperatures exceeding 35°C. All regressions include county fixed effects and year fixed effects. Standard errors (in parentheses) are clustered at county level. Significance: *** p<0.01, ** p<0.05, * p<0.1.

**Table S16. The effects of SUM06, PM_2.5_, and high temperatures on crop and livestock yields**

|  | (1) | (2) | (3) | (4) | (5) | (6) |
| --- | --- | --- | --- | --- | --- | --- |
|  | Maize  yield | Soybean  yield | Single-season rice  yield | Wheat  yield | Tuberous  yield | Milk  per Cow |
| Growing-season  O_3_ measure | -0.0028^***^ | 0.0005 | -0.0017^*^ | -0.0003 | -0.0043^***^ | 0.0009 |
|  | (0.0007) | (0.0011) | (0.0009) | (0.0010) | (0.0014) | (0.0021) |
| PM_2.5_ | 0.0035 | -0.0048 | 0.0038 | -0.0022 | 0.0082^*^ | -0.0081 |
|  | (0.0024) | (0.0033) | (0.0034) | (0.0037) | (0.0049) | (0.0068) |
| ≥35°C | 0.0014 | 0.0012 | -0.0091^***^ | -0.0093^**^ | -0.0078^*^ | 0.0061 |
|  | (0.0026) | (0.0029) | (0.0026) | (0.0036) | (0.0044) | (0.0056) |
| F-test (KP statistics) | 11.6646 | 13.2820 | 32.3320 | 36.6609 | 9.4835 | 9.1636 |
| Observations | 20,892 | 21,833 | 9,291 | 16,095 | 22,868 | 18,683 |

*Notes:* This table presents the IV estimates for the effects of O_3_ (measured using SUM06), PM_2.5_ pollution, and high temperatures during the growing seasons on crop and livestock yields. Columns 1-6 report the results using the natural logarithms of yields for five major crops and milk in China as the dependent variables. The growing seasons for each product are defined as follows: (1) Maize: March – October; (2) Soybeans: May – October; (3) Single-season rice: April – October; (4) Wheat: September (previous year)– August; (5) Tubers: March – November; (6) Milk: the entire year. All regressions include the number of days with daily temperatures falling into specific bins at a width of 5°C, as well as linear and quadratic terms of cumulative precipitation, sunshine duration, average relative humidity, air pressure and wind speed as weather controls. The symbol “$\geq$35°C” denotes the number of days with daily temperatures exceeding 35°C. All regressions include county fixed effects and year fixed effects. Standard errors (in parentheses) are clustered at county level. Significance: *** p<0.01, ** p<0.05, * p<0.1.

**Table S17. Projected changes in agricultural TFP from hypothetical pollution reductions and a scenario of 2℃ warming**

| Scenarios | (1) | (2) | (3) | (4) |
| --- | --- | --- | --- | --- |
| *Panel A: Using TFP derived from four different models* | | | | |
|  | TL-CPF | TL-CPF-w/CRS | CD-CPF | CD-SFA-w/CRS |
| O_3_ meeting the WHO guideline | 60.0% (25.2%, 104.4%) | 54.2% (20.7%, 96.8%) | 49.6% (18.0%, 89.7%) | 55.0% (22.3%, 96.3%) |
| PM_2.5_ reduced to 35 μg/m^3^ | 21.5% (6.1%, 39.0%) | 24.0% (8.8%, 41.4%) | 20.2% (5.8%, 36.5%) | 20.2% (6.0%, 36.3%) |
| 2℃ warming | -2.0% (-3.5%, -0.4%) | -2.1% (-3.6%, -0.6%) | -2.1% (-3.6%, -0.6%) | -2.2% (-3.7%, -0.7%) |
| *Panel B: Using O_3_ predicted from four uncertainty scenarios* | | | | |
|  | A | B | C | D |
| O_3_ meeting the WHO guideline | 49.4% (15.2%, 93.7%) | 35.9% (10.7%, 66.9%) | 69.7% (29.4%,122.5%) | 52.4% (18.1%, 96.8%) |
| PM_2.5_ reduced to 35 μg/m^3^ | 19.8% (5.0%, 36.7%) | 19.7% (5.0%, 36.6%) | 20.9% (5.9%, 38.1%) | 21.7% (6.3%, 39.3%) |
| 2℃ warming | -1.8% (-3.3%, -0.3%) | -2.1% (-3.6%, -0.5%) | -2.4% (-4.0%, -0.9%) | -1.9% (-3.4%, -0.4%) |

*Notes:* The values in parentheses are 95% confidence intervals. In Panel A, the projected changes are based on the regressions with TFP estimated from different models. In Panel B, the projected changes are based on the regressions using O_3_ estimates generated from four uncertainty scenarios.

**Table S18. The effects of extreme O_3_ pollution on agricultural productivity**

| Dependent variable | Log (Agricultural TFP) | | |
| --- | --- | --- | --- |
|  | (1) | (2) | (3) |
|  | AOT40 | AOT60 | AOT80 |
| Non-winter O_3_ measure | -0.0089^***^ | -0.0080^*^ | -0.0357^**^ |
|  | (0.0024) | (0.0041) | (0.0166) |
| PM_2.5_ | -0.0100^***^ | -0.0091^***^ | -0.0101^***^ |
|  | (0.0032) | (0.0034) | (0.0035) |
| ≥35°C | -0.0057^***^ | -0.0045^**^ | -0.0026 |
|  | (0.0020) | (0.0021) | (0.0019) |
| F-test (KP statistics) | 13.4449 | 11.5723 | 12.6816 |
| Observations | 26,788 | 26,788 | 26,788 |

*Notes:* This table shows IV estimates for the effects of pollution and high temperatures above 35°C on agricultural TFP. Various measures of O_3_, including AOT40 (column 1), AOT60 (column 2), and AOT80 (column 3), were used in the analysis. In all columns, we used the TL-CPF model for TFP calculations and an instrumental variable approach for the estimates. All regressions include the number of days with daily temperatures falling into specific bins at a width of 5°C, as well as linear and quadratic terms of cumulative precipitation, sunshine duration, average relative humidity, air pressure and wind speed as weather controls. The symbol “$\geq$35°C” denotes the number of days with daily temperatures exceeding 35°C. All regressions include county fixed effects and year fixed effects. Standard errors (in parentheses) are clustered at county level. Significance: *** p<0.01, ** p<0.05, * p<0.1.

**Table S19. Replication of Chen and Gong (2021) using sample from 2002-2015**

|  | (1) | (2) | (3) | (4) | (5) |
| --- | --- | --- | --- | --- | --- |
|  | CD-SFA-w/CRS | TL-SFA-w/CRS | CD-SFA-w/oCRS | CD-CPF-w/CRS | Yield |
| GDD below 33℃ | 0.0000 | 0.0000 | 0.0000 | 0.0000 | -0.0001 |
|  | (0.0000) | (0.0000) | (0.0000) | (0.0000) | (0.0001) |
| GDD above 33℃ | -0.0011^***^ | -0.0012^***^ | -0.0011^***^ | -0.0010^**^ | -0.0011^**^ |
|  | (0.0003) | (0.0003) | (0.0003) | (0.0004) | (0.0005) |
| Observations | 29,334 | 29,334 | 29,334 | 29,334 | 29,334 |
| R^2^ | 0.8910 | 0.8963 | 0.8856 | 0.7770 | 0.8484 |

*Notes:* This table shows coefficient estimates of temperature variables. The dependent variables are the natural log of agricultural TFP derived from different models. The specifications are the same as that in Chen and Gong (3), including growing degree days (GDD) below and above 33℃, linear and quadratic terms of cumulative precipitation, sunshine duration, average relative humidity, air pressure and wind speed as the weather controls. Both county fixed effects and year fixed effects are included in all regressions. Standard errors (in parentheses) are clustered at county level. Significance: *** p<0.01, ** p<0.05, * p<0.1.

**Table S20. Cumulative indices of O_3_ computed using observed and estimated hourly data**

| (1) | (2) | (3) | (4) | (5) |
| --- | --- | --- | --- | --- |
| Cumulative indices of O_3_ | Unit | Sample means based on observed data | Sample means based on estimated data | Percentage  difference |
| AOT40 | ppm h | 33.4893 | 31.9072 | 4.72% |
| SUM06 | ppm h | 47.0116 | 45.0876 | 4.09% |
| W126 | ppm h | 41.3198 | 36.7376 | 11.09% |

*Notes:* Due to the absence of hourly O_3_ estimates that are essential to accurately calculate cumulative indices of O_3_, we made two simplifying assumptions to compute cumulative indices of O_3_ over the 2002-2015 period. First, we assumed that the hourly O_3_ concentrations during the peak eight hours (or during the non-peak hours) each day in a month are the same, and the O_3_ concentrations during the peak eight hours are equal to the predicted monthly mean MDA8 O_3_ concentrations during this period. Second, we assumed that the ratio of mean O_3_ concentrations during the peak eight hours to that during the non-peak hours, though differing by month and by region, remained stable over the 2002-2019 period. We computed this ratio using the observed hourly data over the 2013-2019 period, and then estimated the mean hourly O_3_ concentrations during the non-peak hours for the years 2002-2015. Column 3 reports the cumulative O_3_ indices computed using ground-recorded hourly data in 2013-2019. Column 4 reports the corresponding estimates using the estimated hourly O_3_ concentrations based on the above assumptions. Column 5 reports the percentage differences between columns 3 and 4. A total number of 479 grid sites were involved in our calculations, and these sites contained ground monitoring stations active over the 2013-2019 period.

**Table S21. Pollutants-pairwise correlation**

|  | O_3_ | PM_2.5_ | PM_10_ | SO_2_ | NO_2_ |
| --- | --- | --- | --- | --- | --- |
| O_3_ | 1 |  |  |  |  |
| PM_2.5_ | -0.2377 | 1 |  |  |  |
| PM_10_ | -0.1708 | 0.8683 | 1 |  |  |
| SO_2_ | -0.2254 | 0.5771 | 0.5943 | 1 |  |
| NO_2_ | -0.2075 | 0.6171 | 0.5801 | 0.4253 | 1 |

*Notes*: This table provides pairwise correlation coefficients between O_3_, PM_2.5_, PM_10_, SO_2_, and NO_2_ in the period of 2013-2019.

**Table S22. Cross validation performance of machine learning models in predicting SO_2_ from 2013 to 2019 at the monthly level**

| Model | R^2^ | RMSE | MAPE |
| --- | --- | --- | --- |
| LightGBM | 0.87 | 6.52 | 22.39% |
| XGBoost | 0.86 | 6.74 | 22.14% |
| Super Learner | 0.88 | 6.44 | 21.59% |

*Notes:* This table shows the overall model performance in predicting SO_2_ employing three machine learning algorithms. RMSE stands for root-mean-squared prediction error. MAPE stands for mean absolute percentage prediction error.

**Table S23. Cross validation performance of machine learning models in predicting NO_2_ from 2013 to 2019 at the monthly level**

| Model | R^2^ | RMSE | MAPE |
| --- | --- | --- | --- |
| LightGBM | 0.88 | 4.61 | 15.12% |
| XGBoost | 0.88 | 4.63 | 15.23% |
| Super Learner | 0.89 | 4.52 | 14.72% |

*Notes:* This table shows the overall model performance in predicting NO_2_ employing three machine learning algorithms. RMSE stands for root-mean-squared prediction error. MAPE stands for mean absolute percentage prediction error.

**Table S24. IV Estimates of the effects of O_3_, PM_2.5_ and high temperatures on agricultural productivity when controlling for other pollutants**

| Dependent variable | Log (Agricultural TFP) | | |
| --- | --- | --- | --- |
|  | (1) | (2) | (3) |
| Annual MDA8 O_3_ | -0.0224^***^ | -0.0359^***^ | -0.0373^***^ |
|  | (0.0070) | (0.0085) | (0.0092) |
| PM_2.5_ | -0.0092^***^ | -0.0078^**^ | -0.0083^**^ |
|  | (0.0033) | (0.0033) | (0.0034) |
| ≥35°C | -0.0050^**^ | -0.0067^***^ | -0.0064^***^ |
|  | (0.0020) | (0.0021) | (0.0021) |
| SO_2_ |  | -0.0110^***^ | -0.0115^***^ |
|  |  | (0.0032) | (0.0033) |
| NO_2_ |  |  | 0.0046 |
|  |  |  | (0.0071) |
| F-test (KP statistics) | 12.4088 | 10.8451 | 9.2180 |
| Observations | 26,788 | 26,788 | 26,788 |

*Notes:* This table presents the IV estimates for the effects of O_3_ (measured using annual MDA8), PM_2.5_ pollution, other air pollutants, and high temperatures on agricultural productivity between 2002 and 2015. Column 1 reports the baseline results. Columns 2 and 3 subsequently add SO_2_ and NO_2_, which are instrumented by wind direction as well. All regressions include the number of days with daily temperatures falling into specific bins at a width of 5°C, as well as linear and quadratic terms of cumulative precipitation, sunshine duration, average relative humidity, air pressure and wind speed as weather controls. The symbol “$\geq$35°C” denotes the number of days with daily temperatures exceeding 35°C. All regressions include county fixed effects and year fixed effects. Standard errors (in parentheses) are clustered at county level. Significance: *** p<0.01, ** p<0.05, * p<0.1.

**References:**

1. Staiger D, Stock JH (1997) Instrumental Variables Regression with Weak Instruments. *Econometrica* 65(3):557–586.

2. Xu X, et al. (2020) Long-term changes of regional ozone in China: Implications for human health and ecosystem impacts. *Elem Sci Anthr* 8(13). doi:10.1525/ELEMENTA.409.

3. Chen S, Gong B (2021) Response and adaptation of agriculture to climate change: Evidence from China. *J Dev Econ* 148:102557.
